# Ferrier Glycosylation Mediated by the TEMPO Oxoammonium Cation

**DOI:** 10.1021/acs.joc.4c00978

**Published:** 2024-08-05

**Authors:** Luis F. Porras-Santos, Jacinto Sandoval-Lira, Julio M. Hernández-Pérez, Leticia Quintero, Pedro López-Mendoza, Fernando Sartillo-Piscil

**Affiliations:** † Centro de Investigación de la Facultad de Ciencias Químicas, Benemérita Universidad Autónoma de Puebla (BUAP), 14 Sur Esq. San Claudio, Col. San Manuel 72570, Puebla, Mexico; ‡ Departamento de Ciencias Básicas, TecNM campus Instituto Tecnológico Superior de San Martín Texmelucan, Camino a la Barranca de Pesos, San Martín Texmelucan 74120, Puebla, Mexico

## Abstract

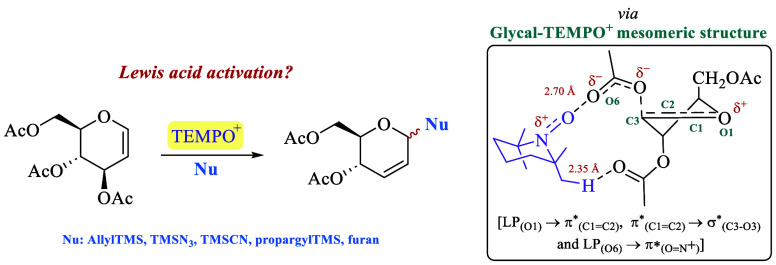

The TEMPO oxoammonium cation has been proven to be both an efficient oxidizing reagent and an electrophilic substrate frequently found in organic reactions. Here, we report that this versatile chemical reagent can also be used as an efficient promoter for C- and N-glycosylation reactions through a Ferrier rearrangement with moderate to high yields. This unprecedented reactivity is explained in terms of a Lewis acid activation of glycal by TEMPO^+^ forming a type of glycal–TEMPO^+^ mesomeric structure, which occurs through an extended vinylogous hyperconjugation toward the π*_(O=N^+^__)_ orbital [LP_(O1)_ → π*_(C1=C2)_, π*_(C1=C2)_ → σ*_(C3–O3)_, and LP_(O6)_ → π*_(O=N_^+^_)_]. This enables the formation of the respective Ferrier glycosyl cation, which is trapped by various nucleophiles. The extended hyperconjugation (or double hyperconjugation) toward the π*_(O=N^+^__)_ orbital, which confers the Lewis acid character of the TEMPO cation, was supported by natural bond orbital analysis at the M06-2X/6-311+G** level of theory.

## Introduction

**Scheme 1:**
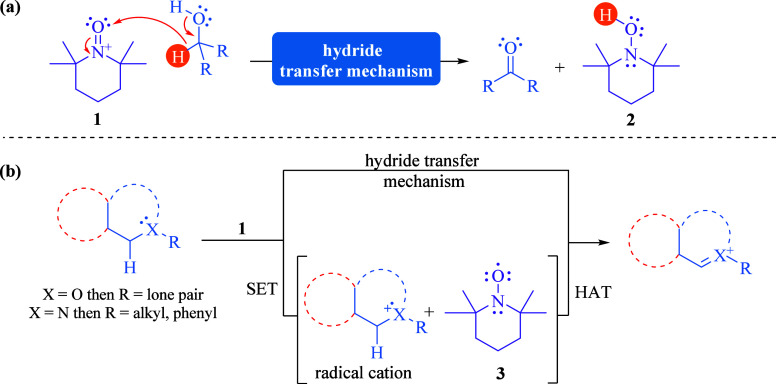
Mechanistic Paths for the TEMPO^+^-Mediated Oxidative Reaction: (a) Hydride Transfer Mechanism for Alcohols and (b) Hydride Transfer and SET/HAT Mechanisms for C–H Functionalization

Since the publication of Golubev’s pioneering work more than 50 years ago,^1^ the 2,2,6,6-tetramethylpiperidine-derived *N*-oxoammonium cation (TEMPO^+^, **1**) has been widely used as a sustainable oxidizing reagent^2^ to convert alcohols into carbonyl compounds.^3^ More recently, this versatile reagent has been employed not only for mono-C–H functionalization at the α position of N- and O-heterocycles^4a^ but also for the selective multiple-C–H functionalization of N-heterocycles.^4b^ Another attractive feature of **1** is the fact that most chemical transformations are performed under mild and operationally simple reaction conditions in both catalytic^5^ and stoichiometric^6^ fashions. Due to the close chemical relationship among the corresponding 2,2,6,6-tetramethylpiperidine-1-oxyl (TEMPO, **3**), hydroxylamine (TEMPOH, **2**), and **1** (formed through a redox process),^7^ considerable attention has been devoted to mechanistic studies, especially for analyzing the oxidation of alcohols.^8−10^ With a few mechanistic exceptions,^8,9^ several computational studies agree about the ability of the TEMPO^+^ oxygen atom to accept a hydride ion during the oxidative process.^10^ Therefore, over the past few years, this hydride transfer model has gained significance and has become the prevalent mechanistic proposal (Scheme 1a).^10b,11^

Conversely, for oxoammonium-mediated dehydrogenative functionalization, experimental evidence is reported not only for the hydride transfer pathway^12^ but also for a mechanism involving a single-electron transfer (SET) from the substrate to **1** followed by a hydrogen atom transfer (HAT) (Scheme 1b).^13^ These findings have allowed further exploration of chemical transformations involving radical processes.^14^ However, recent reports describing **1** as an oxygen atom transfer reagent continue to rely on the initial addition of nucleophiles to the oxygen atom, highlighting the prevalence of the electrophilicity of **1**.^15^

**Scheme 2:**
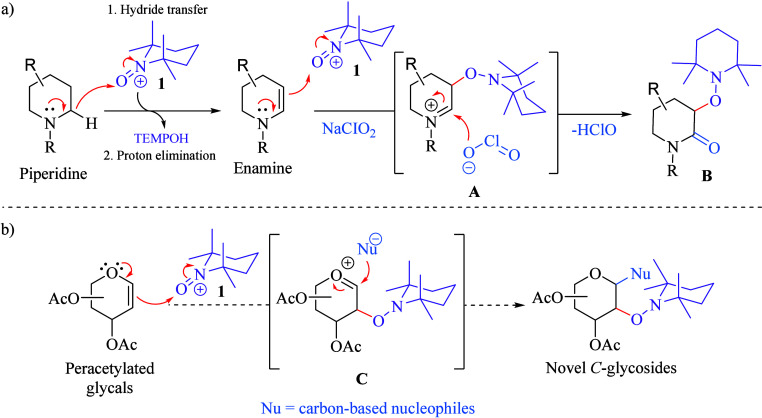
(a) Duality of TEMPO^+^ Reactivity on Saturated N-Heterocycles and (b) Proposed Double Functionalization of Peracetyl Glycals by TEMPO^+^

In this context, over the past eight years,^16^ our research group has been interested in expanding the use and mechanistic findings of TEMPO^+^**1** beyond the C–H oxidation of hydroxyl groups. An important feature of the chemistry of **1** is that it readily reacts with piperidines to form transient enamine intermediates, which are attacked by an additional 1 equiv of **1** to form a key iminium intermediate **A** and finally the corresponding 3-alkoxyaminolactam **B** (Scheme 2a).^16a^

Similarly, the opportunity to expand this dual reactivity of **1** to peracetylated glycals was visualized with the expectation of generating an oxocarbenium ion **C**, which could be attacked by suitable carbon-based nucleophiles to enable the formation of novel *C*-glycosides bearing the alkoxyamino group at position C2, opening thus the possibility for further functionalization (Scheme 2b).

## Results and Discussion

Both 3,4,6-tri-*O*-acetyl-d-glucal **4** and allyltrimethylsilane (ATMS) were selected for the initial experiments. When **4** was allowed to react with 5.0 equiv of ATMS and 2.0 equiv of **1**·BF_4_^–^ in 1,2-dichloroethane (1,2-DCE) at room temperature for 2 h, the starting material remained unchanged (Table 1, entry 1). After the temperature was increased to 70 °C for 5 h, half of the starting material was consumed, although the expected product **5** was not observed; instead, a *C*-allylglycoside **6** was obtained as the sole product with a low chemical yield (entry 2). Interested by this unprecedented TEMPO^+^-catalyzed C-glycosylation through a Ferrier rearrangement,^17^ we attempted to optimize this reaction.

**Table 1:**
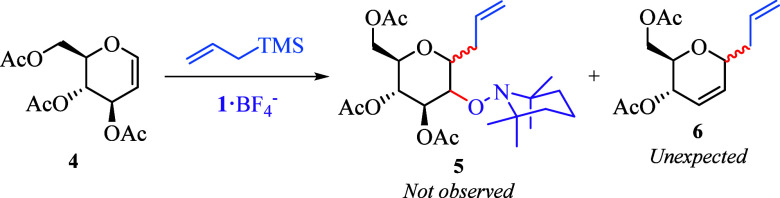
Unexpected Ferrier C-Glycosylation Mediated by TEMPO^+^**1**

entry	solvent^e^	ATMS (equiv)	TEMPO^+^BF_4_^–^ (equiv)	temp (°C)	time (h)	yield (%)^f^
1^a^	1,2-DCE	5.0	2.0	rt	2	no reaction
2^b^	1,2-DCE	5.0	2.0	70	5	28
3^c^	1,2-DCE	5.0	2.0	70	1.5	60
4	1,2-DCE	7.0	1.0	70	1	65
5	1,2-DCE	7.0	1.0	70	1	45
6	DCM	7.0	2.0	50	2	15
7	CH_3_CN	7.0	2.0	70	1	80
8^d^	CH_3_CN	7.0	2.0	70	0.33	80
9	CH_3_CN	7.0	0	70	0.5	no reaction

a Reaction conditions: 0.11 mmol of **4**, 0.55 mmol of **7**, and 0.22 mmol of **1a** in 1,2-DCE at room temperature.

b Oil bath as the energy source.

c Entries 3–9 used microwave irradiation as the energy source.

d Best reaction conditions.

e With 0.5 M glucal.

f Isolated yields.

First, we changed the traditional heating by microwave (MW) irradiation at 70 °C, and the chemical yield of **6** was increased to 60% in just 1.5 h (entry 3). To ensure the consumption of **4**, the number of equivalents of ATMS was increased to 7.0 under MW irradiation, and the chemical yield was improved to 65% in barely 1 h (entry 4). Decreasing the number of equivalents of **1**·BF_4_^–^ to 1.0 decreased the yield to 45% (entry 5). Interestingly, switching the solvent from 1,2-DCE to dichloromethane (DCM) resulted in a dramatic decrease in the yield (entry 6). On the contrary, an attractive 80% yield was obtained by using acetonitrile at 70 °C for 1 h (entry 7), and the same high yield was obtained in only 30 min under the same reaction conditions (entry 8). Finally, control experiments showed that this reaction requires the use of **1**·BF_4_^–^ (entry 9).

After establishing the optimal conditions, we explored the scope of this unprecedented TEMPO^+^-promoted glycosylation reaction, using other nucleophiles and peracetylated glycals (Table 2). While glycosylation of **4** with trimethylsilyl cyanide (TMSCN) produced glycosyl cyanide **7** in an excellent yield at a 3:2 α:β ratio (entry 1), trimethylsilyl azide (TMSN_3_) gave a regioisomeric mixture of glycosyl azides **8a** and **8b** in a combined 96% yield of 1:1 and 7:3 α:β ratios, respectively (entry 2). Unlike the C-glycosylation of **4** with TMSCN, where heating at 70 °C was required, the reaction proceeded efficiently with only 2.0 equiv of TMSN_3_ at 50 °C. The use of trimethyl(propargyl)silane as a nucleophile enabled the corresponding anomeric α-allene **9** in a modest 40% yield (entry 3). Furthermore, when furan was employed as a nucleophile, *C*-glycosyl regioisomers **10a** (only α) and **10b** (only α) was obtained in good yield (entry 4). Distinctly, due to the inherent aromatic stability of furans, the complete consumption of the starting material took longer. Interestingly, with regard to 3,4,6-tri-*O*-acetyl-d-galactal **11**, C-glycosylation with ATMS gave a high yield and an α diastereoselectivity of expected C-allylated product **12** (entry 5). Whereas glycosyl cyanide **13** was obtained in low yield and modest stereoselectivity (entry 6), N-glycosylation proceeded in high yield but moderate regioselectivity to give glycosyl azides **14a** and **14b** (entry 7). In turn, 3,4-di-*O*-acetyl-d-xylal **15** behaved like **4** and **11**, yielding products **16** and **17**, as well as a regioisomeric mixture of **18a** and **18b** (entries 8–10, respectively), when treated with the appropriate nucleophiles. Moreover, it is worth noting that, whereas 2.0 equiv of **1** was needed to achieve satisfactory C- and N-glycosylation of **4** and **11**, only 0.5 equiv was used to obtain very similar results with **15** (discussed in Mechanistic Studies).

**Table 2:**
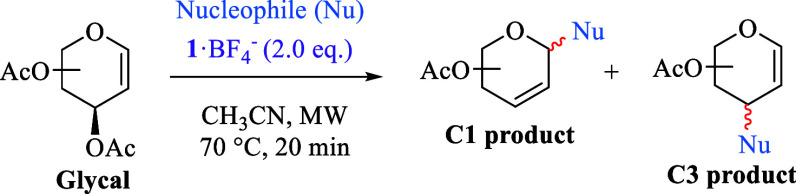

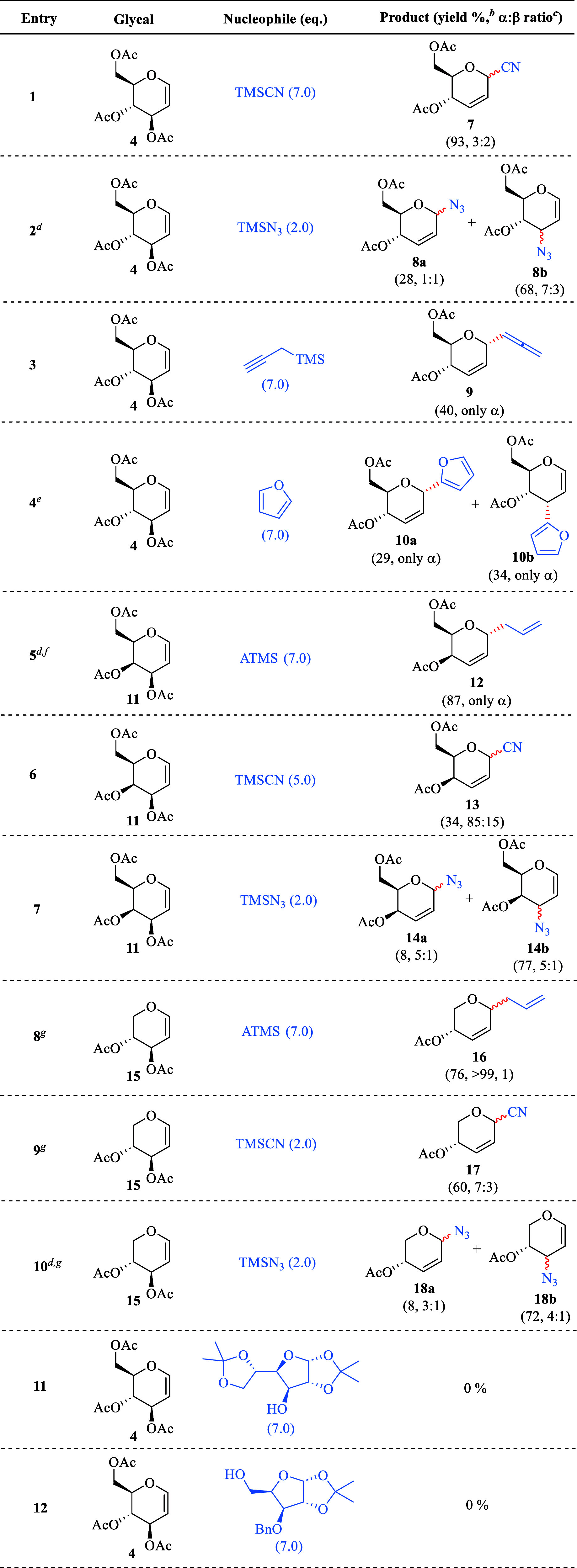
Scope of the TEMPO^+^-Mediated C- and N-Glycosylations^a^

a Reaction condition: glycal (0.11 mmol, 1.0 equiv), **1**·BF_4_^–^ (0.22 mmol, 2.0 equiv), and CH_3_CN (0.5 M) at 70 °C for 20 min unless otherwise noted.

b Isolated and optimized yields.

c Determined by ^1^H NMR spectroscopy of relative integration of anomeric or separable protons.

d At 50 °C.

e For 30 min.

f For 10 min.

g Only 0.5 equiv of **1**·BF_4_^–^ salt.

In addition, diacetone d-glucose and *O*-benzylxylofuranose were tested to obtain the corresponding *O*-glycosides from **4**, but only the decomposition of the alcohols was observed (entries 11 and 12). Finally, to demonstrate the practical utility of this TEMPO^+^-mediated Ferrier glycosylation, an experiment at a 2.5 mmol scale using compound **4** was performed, from which *C*-glycoside **6** was obtained with an almost identical yield (see the Supporting Information).

## Mechanistic Studies

### Experimental Studies

**Scheme 3:**
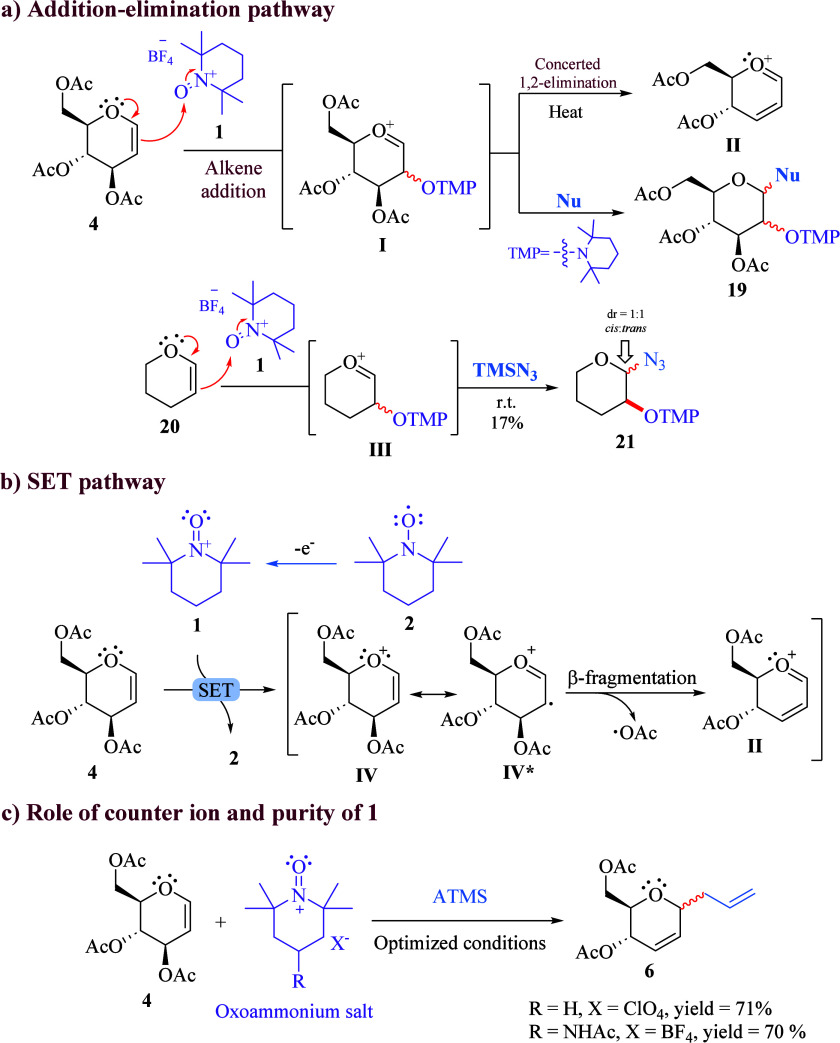
Plausible Pathways for TEMPO^+^-Mediated Glycosylation Reactions

Intrigued by this unprecedented reactivity of **1**, we explored several possible paths to explain our results. First, an addition–elimination pathway (Scheme 3a) was considered, based on the well-known electrophilicity of the R_2_N^+^=O bond in **1**.^15,16^ Accordingly, the electron-rich double bond of glycal **4** might be attacked by **1** to initially form 3-alkoxyamine oxocarbenium cation **I**, followed by a concerted 1,2-elimination^18^ to give key vinylic oxocarbenium intermediate **II**, which could be further attacked by nucleophiles. However, if this mechanism were to operate, **I** could also undergo nucleophilic attacks to form **19**. In this regard, after several detailed experiments and examination of the crude reaction mixtures, we were unable to detect **19** in any of the glycosylation reactions, even when **4** was treated with TMSN_3_ at 40 °C (the lowest temperature at which the reaction proceeds). In contrast, when a similar system such as 3,4-dihydro-2*H*-pyran **20** reacted with **1** and TMSN_3_, 1,2-substituted tetrahydropyrans **21** were obtained. To further exclude the 1,2-elimination process, compounds **21** were heated in acetonitrile at 70 °C; the starting material remained unchanged. Therefore, we concluded that vinylic oxocarbenium **II** does not come from putative intermediate **I**. Subsequently, a plausible SET mechanism was also investigated (Scheme 3b).^13,19^ For this, a SET process was envisioned to occur between glycal **4** and TEMPO^+^, which could generate both TEMPO and radical cation **IV**, and together with its resonance contributor IV* would produce vinyl oxocarbenium cation **II** after a β-fragmentation. Although this process could be related to that proposed for CAN-mediated C-glycosylation,^19^ the substoichiometric amounts of the TEMPO^+^ salt (0.5 equiv) required for glycosylation reactions of 3,4-di-*O*-acetyl-d-xylal **15** suggest that both reagents operate under two different reaction mechanisms. Furthermore, radical trapping experiments were performed with 2,6-di-*tert*-butyl-4-methylphenol (BHT), and although a significant yield reduction was observed, it was not possible to trap any radical intermediate. Indeed, the TEMPO^+^ salt decomposed in the presence of BHT. Moreover, recent computational and experimental calculations of electrochemical potentials suggest that this process is unlikely due to the high redox potential of cyclic vinyl ethers.^20^

Another concern arose regarding the activating role of **1**. Could BF_3_ and F^–^ be responsible of the Ferrier glycosylation because both might be formed from thermal decomposition of **1**?^21^ To address this question, it was necessary to use analogue salt **1**·ClO_4_^–^ as a different Ferrier rearrangement promoter. The reaction proceeded as expected to give a similar chemical yield of **6** [71% (Scheme 3c)]. Finally, considering the reports on the use of both HBF_4_ and HClO_4_ supported over SiO_2_ for catalyzed Ferrier glycosylation^22^ and the probable presence of traces of TEMPOH-HBF^4^ that accumulated during the preparation of **1**·BF_4_^–^,^23^ it was necessary to perform the glycosylation using a highly pure TEMPO^+^ source. To this end, Bobbitt’s salt was purchased directly from Sigma-Aldrich (purity of >96.5%, verified by HPLC) and subjected to reaction with glycal **4** under the optimized reaction conditions. The experiment provided compound **6** in practically the same chemical yield [70% (Scheme 3c)] as the yields of those performed by **1**·BF_4_^–^, demonstrating that the glycosylation reactions are mediated by **1** in an unprecedented activation mode.

### Conformational Study

**Scheme 4:**
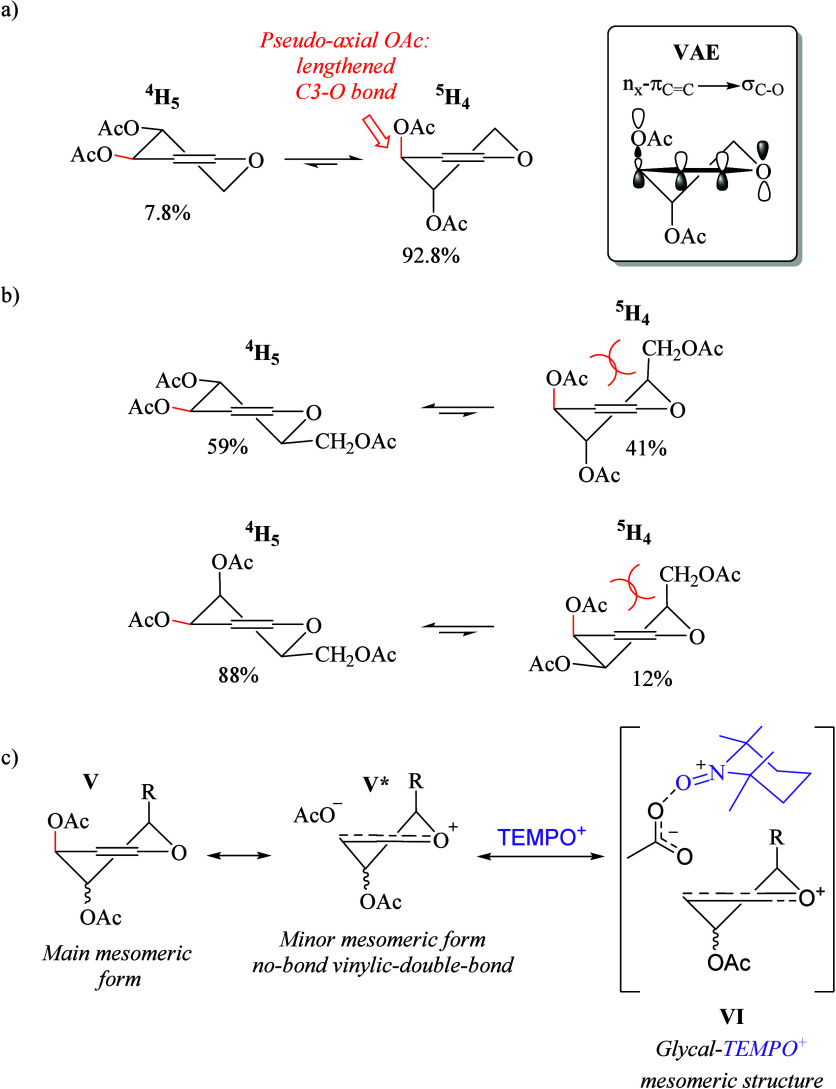
(a) Vinylogous Anomeric Effect on C3–OAc Glycals, (b) Vinylogous Anomeric Effect on C3–OAc 5-Substituted Glycals, and (c) Mesomeric Forms on C3–OAc Glycals and Extended Hyperconjugation to TEMPO^+^**1**

It is well-known that the conformational population of peracetylated glycals dictates their reactivity in the Ferrier rearrangement.^24a,24b^ This is largely because the endocyclic double bond between C1 and C2 leads to two possible half-chair conformations, namely, ^4^H_5_ and ^5^H_4_, whose equilibrium is mainly mediated by the vinylogous anomeric effect (VAE), which consists of the hyperconjugation between the lone electron pair of the endocyclic ring oxygen and the C3–O2 antibonding orbital, through the π_C1=C2_ bonding orbital [LP_(O1)_ → π_(C1=C2)_ → σ*_(C3–O2)_].^24a,24b^ These orbital interactions not only favor the preferred ^5^H_4_ conformation directing the OAc at C3 pseudoaxially (Scheme 4a) but also weaken the C3–O bond of glycals, increasing its reactivity.^24c,24d^ A clear manifestation of the VAE is observed in 3,4-di-*O*-acetyl-d-xylal **15**, in which the ^5^H_4_ conformation is highly favored relative to the ^4^H_5_ conformation (Scheme 4a),^25a^ resulting in a remarkable increase in reactivity, up to the point that only 0.5 equiv of **1** suffices to perform the complete glycosylation reaction. In contrast, the presence of the C5 substituent in 3,4,6-tri-*O*-acetyl-d-glucal **4** and 3,4,6-tri-*O*-acetyl-d-galactal **11** destabilizes their ^5^H_4_ conformations due to the existence of 1,3-diaxial interactions between the pseudoaxial C3–OAc and C5 substituents (Scheme 4b).^25b,25c^ Evidently, these interactions compromise the VAE, and consequently, both **4** and **5** are less reactive toward **1** (i.e., 2.0 equiv of **1** is required for the glycosylation of **4** and **5**). Therefore, the inherent reactivity of glycals is better depicted by considering their no-bond vinylic double-bond mesomeric form (e.g., V*).^26^ As the natural VAE is not enough to promote the formation of the vinylic oxocarbenium cation from **V** ↔ V* to eventually be attacked by nucleophiles, we hypothesized that TEMPO^+^**1** activates the minor mesomeric form no-bond vinylic double-bond V*, extending the VAE toward the π*_O=N^+^_ orbital of **1** forming a highly reactive glycal–TEMPO^+^ mesomeric structure **VI** (Scheme 4c). To the best of our knowledge, the reactivity of cation **1** as a Lewis acid activator has not yet been proposed. Probably the closest precedent is found in the work of Song and co-workers,^27a^ in which **1** is employed as an activator of haleniums for the selective halogenation of olefins. Additionally, although the Ferrier rearrangement mechanism mediated by TEMPO^+^**1** is quite different from that proposed for the tertiary allylic alcohol rearrangements, we cannot exclude the possibility that TEMPO^+^**1** could act as a masked tertiary carbocation for OAc activation.^27b^

### Computational Studies

**Figure 1:**
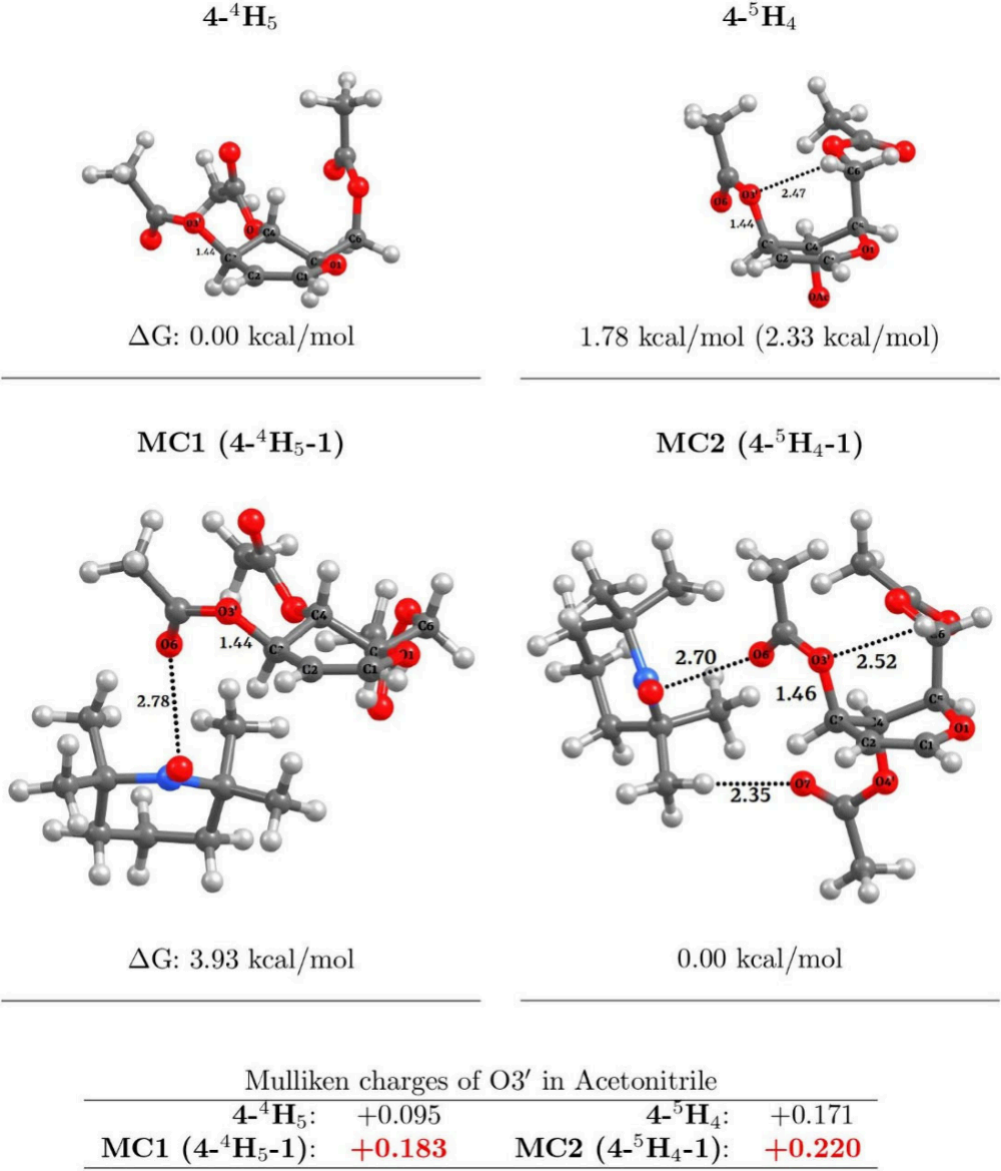
Optimized geometries and relative energies calculated at the M06-2X/6-311+G** level of theory in a vacuum. The value in parentheses is for acetonitrile. Top: **4**-^4^H_5_ and **4**-^5^H_4_. Bottom: **4**-^4^H_5_-**1** and **4**-^5^H_4_-**1**.

To provide insights into the role of **1** in the Ferrier C-glycosylation reaction, we performed molecular orbital calculations to evaluate the VAE of molecular complexes (MCs) formed from the interaction between **4** and **1**. First, the optimized geometry of major conformers of **4** (**4**-^4^H_5_ and **4**-^5^H_4_) was obtained; subsequently, molecular complexes **MC1** and **MC2** (**4**-^4^H_5_-**1** and **4**-^5^H_4_-**1**, respectively) were calculated (see Figure 1).

The free energy (Δ*G*) at the M06-2X/6-311+G** level of theory showed that conformer **4**-^4^H_5_ is more stable than **4**-^5^H_4_ by 1.78 kcal/mol in a vacuum and by 2.33 kcal/mol in acetonitrile. On the contrary, the relative free energy (Δ*G*) comparison between **MC1** and **MC2**, which are the proposed glycal–TEMPO^+^ mesomeric structures, showcases that **MC2** is more stable by 3.93 kcal/mol in a vacuum. In acetonitrile, **MC1** and **MC2** are isoenergetic (see Tables S7 and S8). In addition, whereas **MC1** and **MC2** present an O6···O=N interaction with distances of 2.78 and 2.70 Å, respectively, only **MC2** shows weak C–H···O intermolecular interactions. In particular, these interactions between a C–H bond from a methyl group of **1** and an O7=C bond from an acetyl group at C3 of **4** with a distance of 2.35 Å and between C–H6 and O3 (2.52 Å) are believed to be the responsible for the greater stability of **MC2**. Similar C–H hydrogen bonding interactions from a methyl group of **1** have been computationally observed in the selective C–H functionalization of *N*-benzyl piperidines by **1**.^16d^

The proposed interactions were validated by calculating the complexation energies of **MC1** and **MC2** at the M06-2X/6-311+G** level of theory, considering the basis set superposition error (BSSE) correction in a vacuum and acetonitrile. It was shown that the complexation energy without BSSE is negative (−20.20 kcal/mol for **MC1** and −20.44 kcal/mol for **MC2** in a vacuum), thus confirming the stability of the proposed molecular complexes. Introducing the BSSE correction increases the complexation energy, compared to that calculated without the BSSE correction. The complexation energies were found to be −18.44 kcal/mol for **MC1** and −19.03 kcal/mol for **MC2** in a vacuum. The complexation energies in acetonitrile with and without BSSE are −16.20 and −14.38 kcal/mol, respectively, for **MC1** and −18.96 and −17.61 kcal/mol, respectively, for **MC2** (see Table S9). The complexation electronic energy values with ZPE for **MC1** and **MC2** are −5.00 and −4.43 kcal/mol, respectively. These values are in excellent agreement with recent reports of TEMPO^+^ (and its analogues) being an electrophilic activator of either the carbonyl group or the bromine atom.^27a^ The atomic charges were calculated using Mulliken population analysis. The calculated atomic charges show that the positive charge on O3′ in complexes **MC1** (+0.1855) and **MC2** (+0.220) is larger than that of isolated **4**-^4^H_5_ (+0.095) and **4**-^5^H_4_ (+0.171) (see Figure 1). These results suggest that TEMPO^+^ activates **4** as a Lewis acid catalyst through formation of the **MC1** and **MC2** complexes, where the O3′ atom increases its electrophilicity, favoring the fragmentation of the C3–O3′ bond to promote the glycal–TEMPO^+^ mesomeric structure (see Scheme 4c).

**Figure 2:**
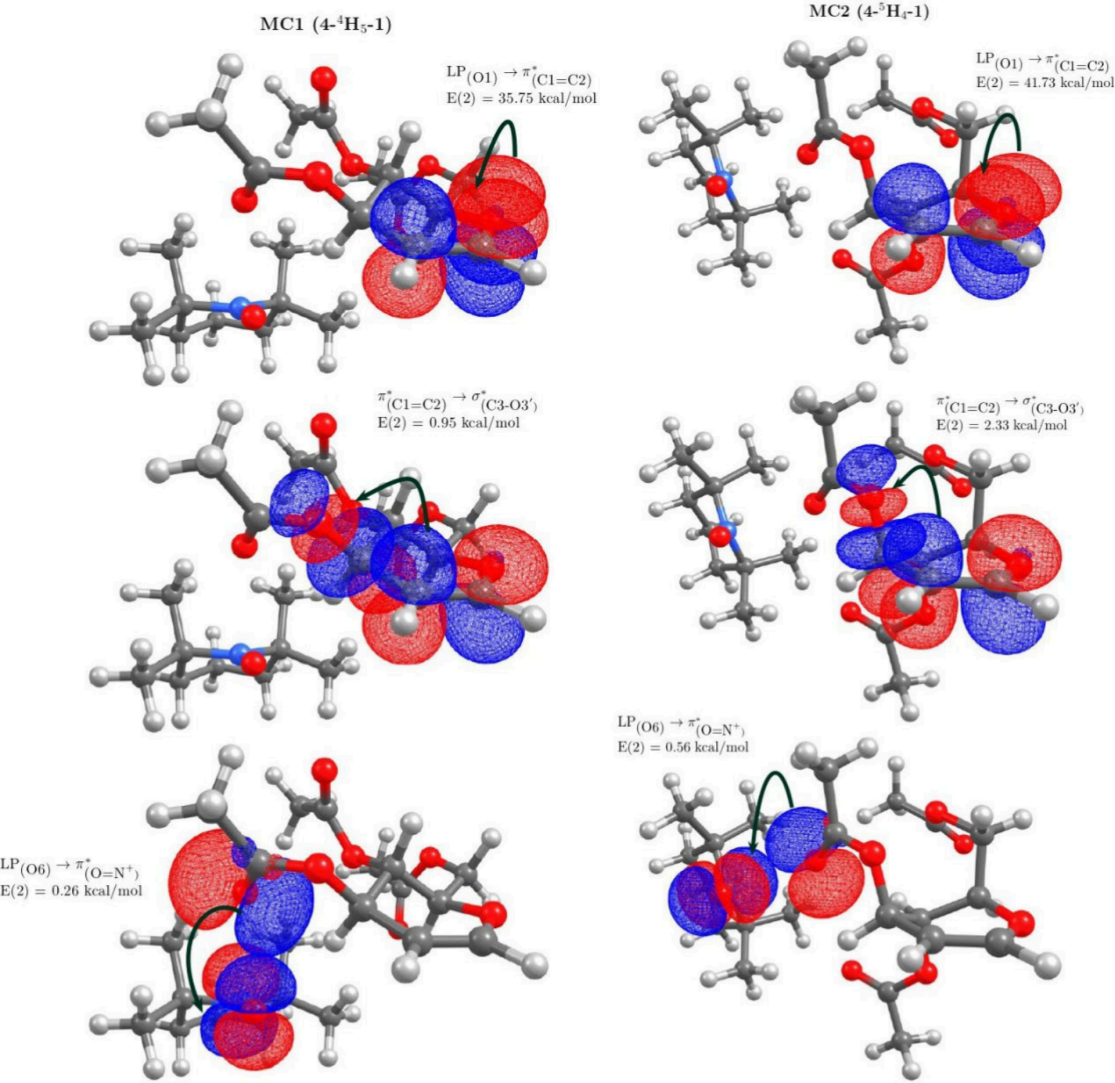
NBO interactions of MC1 (**4**-^4^H_5_-**1**) and MC2 (**4**-^5^H_4_-**1**). All plots in which NBOs are depicted were rendered using an isovalue of 0.03 au.

Interestingly, the natural bond orbital (NBO) analysis of **MC1** and **MC2** (Figure 2) confirmed our proposal on the extended VAE of **4** toward the π*_(O=N^+^__)_ orbital of **1** [LP_(O1)_ → π*_(C1=C2)_, π*_(C1=C2)_ → σ*_(C3–O3)_, and LP_(O6)_ → π*_(O=N^+^__)_]. Accordingly, for **MC1**, the energy value [*E*(2)] for the first hyperconjugation, LP_(O1)_ → π*_(C1=C2)_, is 35.76 kcal/mol, that for the second hyperconjugation, π*_(C1=C2)_ → σ*_(C3–O3)_, is close to 1.0 kcal/mol, and an extended hyperconjugation is barely detected [LP_(O6)_ → π*_(O=N^+^__)_, 0.26 kcal/mol] (Figure 2). However, **MC2**, which orients the C3-acetyl group pseudoaxially, presents a much more efficient extended hyperconjugation, which suggests a greater reactivity toward nucleophiles. Indeed, **MC2** is ∼2 times more efficient than **MC1**, especially for the second and extended hyperconjugations: *E*(2) = 2.33 kcal/mol for π*_(C1=C2)_ → σ*_(C3–O3)_, and *E*(2) = 0.56 kcal/mol for LP_(O6)_ → π*_(O=N^+^__)_. The π*_(C1=C2)_ → σ*_(C3–O3)_ interaction in **MC2** is a measure of the uninterrupted electronic delocalization of the LP_(O1)_ orbital; through the π*_(C1=C2)_ nonbonding orbital, these orbital interactions can be understood as through-bond interaction or double hyperconjugation^24b^ that favors the fragmentation of the C3–O3 bond promoted by the LP_(O6)_ → π*_(O=N^+^)_ interaction that gives rise to the glycal–TEMPO^+^ mesomeric structure shown in Scheme 4c.

## Conclusions

In summary, we have demonstrated that TEMPO^+^ efficiently promotes the Ferrier rearrangement of glycals for the C- and N-glycosylation reactions. Theoretical calculations at the M06-2X/6-311+G** level of theory suggest that TEMPO^+^ acts as a Lewis acid, enabling the formation of the key vinylic oxocarbenium intermediate. The unprecedented reactivity of TEMPO^+^ originated from the extended hyperconjugation of the inherent vinylogous anomeric effect of C3-acetylated glycals to the π*_(O=N^+^__)_ orbital of TEMPO^+^, which in turn generate a highly reactive glycal–TEMPO^+^ mesomeric structure. This computational rationale provides further evidence of the influence of the double hyperconjugation or through-bond interaction on chemical reactivity. In addition, the experimental results open the door to the exploration of novel synthetic applications of TEMPO^+^ salts as an air and moisture stable Lewis acid beyond their classical use as oxidizing and electrophilic reagents. Accordingly, we continue our research, and further studies will be reported in due course.

## Computational Methods

All calculations were performed using Gaussian 09,^28^ and all structures were visualized using Chemcraft 1.6.^29^ We carried out the complete set of calculations using the M06-2X functional^30^ with the 6-311+G** basis set. All minimum structures were validated by subsequent frequency calculations at the same level of theory. The minimum structures have a set of positive second derivatives. All calculations included SMD as an implicit solvation model (acetonitrile). The complexation energies of **MC1** and **MC2** were calculated for the optimized geometries in a vacuum and acetonitrile utilizing the counterpoise method.^31^ Electronic structures were studied by using NBO analysis, and the stabilizing energies were calculated by second-order perturbation theory analysis.^32,33^ Isosurfaces with value of 0.03 au were used to depict NBOs.

## Experimental Section

All reactions were carried out using microwave irradiation in a sealed tube under an inert nitrogen atmosphere with dry solvents, unless otherwise specified. Commercially available reagents were purchased from Sigma-Aldrich and used without further purification. Acetonitrile, dichloromethane (DCM), and 1,2-dichloromethane (1,2-DCE) were used as reactive grade reagents, dried using standard techniques, and freshly distilled prior to use. Column chromatography (CC) was performed using silica gel 230–400 mesh as the stationary phase and a mixture of solvents as the mobile phase. Reactions were monitored by thin-layer chromatography on 0.25 mm Merk silica gel 60-F254 plates using ultraviolet (UV) light, anisaldehyde, potassium permanganate, or ammonium molybdate stain as the visualizing agent. Microwave experiments were performed in a CEM Discover System (model 908005) microwave reactor in sealed tubes. High-resolution mass spectra (HRMS) were recorded in fast atom bombardment (FAB) mode by using a QMS mass analyzer. Nuclear magnetic resonance (NMR) spectra were recorded on a Bruker-500 (500 MHz) spectrometer using as a reference TMS (0.0 ppm for ^1^H) and the residual solvent peak of CDCl_3_ (7.26 ppm for ^1^H NMR and 77.16 ppm for ^13^C). Chemical shifts (δ) are stated in parts per million, and coupling constants (*J*) are in hertz. The following abbreviations (or combinations thereof) were used to explain the multiplicities: s, singlet; d, doublet; t, triplet; q, quartet; m, multiplet; br, broadened.

### General Procedure for TEMPO^+^-Mediated Glycosylation of Glycals

TEMPO^+^BF_4_^–^ (0.5–2.0 equiv) and glycal were dissolved in anhydrous CH_3_CN (0.5 M) under a nitrogen atmosphere in a flame-dried sealed tube. Afterward, the corresponding nucleophile (2.0–7.0 equiv) was added to the solution at room temperature. The reaction mixture was stirred and heated in a microwave reactor at a specified temperature (*T*) for a predetermined time (*t*) using a power of 70 W. The values of *T* and *t* for each glycal–nucleophile pair are listed in Tables 1 and 2. Upon completion of the reaction, the solvent was removed under vacuum, and the residue was purified via flash column chromatography on silica gel using a hexane/EtOAc eluent system.

#### [(2*R*,3*S*)-3-Acetoxy-6-allyl-3,6-dihydro-2*H*-pyran-2-yl]methyl Acetate (**6**)

Compound **6** was prepared following the general procedure using compound **4** (30 mg, 0.110 mmol, 1.0 equiv) as the starting material, ATMS as the nucleophile (0.12 mL, 0.771 mmol, 7.0 equiv), and **1**·BF_4_ (53.5 mg, 0.220 mmol, 2.0 equiv). The crude reaction mixture was purified by flash column chromatography (9:1 hexanes/EtOAc) to give **6** (22.4 mg, 80% yield) as a colorless oil. Major α isomer (76.2% yield): ^1^H NMR (500 MHz, CDCl_3_) δ 5.91 (d, *J* = 10.5 Hz, 1H), 5.87–5.75 (m, 2H), 5.15–5.06 (m, 3H), 4.30–4.24 (m, 1H), 4.24–4.18 (m, 1H), 4.13 (dt, *J* = 12.1, 3.0 Hz, 1H), 3.98–3.91 (m, 1H), 2.45 (dt, *J* = 14.9, 7.5 Hz, 1H), 2.30 (dt, *J* = 13.9, 6.5 Hz, 1H), 2.07 (s, 6H); ^13^C{^1^H} NMR (125 MHz, CDCl_3_) δ 170.9, 170.5, 134.1, 132.9, 123.8, 117.7, 69.8, 65.1, 63.0, 38.0, 21.2, 20.9. Minor β isomer (3.8% yield): ^1^H NMR (500 MHz, CDCl_3_) δ 5.88–5.75 (m, 3H), 5.25 (d, *J* = 9.4 Hz, 1H), 5.15–5.09 (m, 2H), 4.27–4.21 (m, 3H), 4.15 (dd, *J* = 12.1, 6.2 Hz, 1H), 3.73 (d, *J* = 7.3 Hz, 1H), 2.37 (d, *J* = 7.5 Hz, 1H), 2.29 (dt, *J* = 14.0, 6.9 Hz, 1H), 2.10 (s, 3H), 2.08 (s, 3H); ^13^C{^1^H} NMR (125 MHz, CDCl_3_) δ 170.9, 170.4, 135.5, 132.5, 125.2, 117.8, 74.4, 74.4, 65.8, 63.8, 39.5, 21.2, 21.0. The spectroscopic data agree with the values reported in ref (34).

#### [(2*R*,3*S*)-3-Acetoxy-6-cyano-3,6-dihydro-2*H*-pyran-2-yl]methyl Acetate (**7**)

Compound **7** was prepared following the general procedure using compound **4** (30 mg, 0.110 mmol, 1.0 equiv) as the starting material, TMSCN as the nucleophile (0.09 mL, 0.771 mmol, 7.0 equiv), and **1**·BF_4_ (53.5 mg, 0.220 mmol, 2.0 equiv). The crude reaction mixture was purified by flash column chromatography (9:1 hexanes/EtOAc) to give **7** (24.5 mg, 93% yield) as a colorless oil. Major α isomer (55.8% yield): ^1^H NMR (500 MHz, CDCl_3_) δ 6.03 (dt, *J* = 10.2, 1.9 Hz, 1H), 5.89 (ddd, *J* = 10.3, 3.7, 1.9 Hz, 1H), 5.36–5.30 (m, 1H), 5.07 (dd, *J* = 3.7, 1.9 Hz, 1H), 4.26 (d, *J* = 3.9 Hz, 2H), 4.06–4.00 (m, 1H), 2.11 (s, 3H), 2.10 (s, 3H); ^13^C{^1^H} NMR (126 MHz, CDCl_3_) δ 170.8, 170.2, 129.8, 123.7, 115.7, 72.2, 63.9, 62.8, 62.4, 21.0, 20.8. Minor β isomer (37.2% yield): ^1^H NMR (500 MHz, CDCl_3_) δ 6.03 (dt, *J* = 10.3, 2.4 Hz, 1H), 5.91 (dt, *J* = 10.1, 2.1 Hz, 1H), 5.29 (dt, *J* = 6.4, 2.5 Hz, 1H), 5.12 (t, *J* = 2.4 Hz, 1H), 4.30–4.23 (m, 1H), 4.19 (dd, *J* = 12.5, 6.0 Hz, 1H), 3.81 (ddd, *J* = 8.3, 5.7, 2.5 Hz, 1H), 2.11 (s, 2H), 2.09 (s, 2H); ^1^^3^C{^1^H} NMR (125 MHz, CDCl_3_) δ 170.8, 170.1, 128.9, 124.3, 115.8, 74.5, 63.6, 63.1, 62.6, 21.0, 20.9. The spectroscopic data agree with the values reported in ref (34).

#### [(2*R*,3*S*)-3-Acetoxy-6-azido-3,6-dihydro-2*H*-pyran-2-yl]methyl Acetate (**8a**) and [(2*R*,3*S*)-3-Acetoxy-4-azido-3,4-dihydro-2*H*-pyran-2-yl]methyl Acetate (**8b**)

Compound **8** was prepared following the general procedure using compound **4** (30 mg, 0.110 mmol, 1.0 equiv) as the starting material, TMSN_3_ as the nucleophile (0.03 mL, 0.220 mmol, 2.0 equiv), and **1**·BF_4_ (53.5 mg, 0.220 mmol, 2.0 equiv). The crude reaction mixture was purified by flash column chromatography (9:1 hexanes/EtOAc) to give **8** (27.0 mg, 96% yield) as a colorless oil: ^1^H NMR (500 MHz, CDCl_3_) δ 6.52 (d, *J* = 6.0 Hz, 1.62H, α-C3 isomer), 6.49 (d, *J* = 6.6 Hz, 1.44H, β-C3 isomer), 6.03 (d, *J* = 10.4 Hz, 0.38H, β-C1 isomer), 5.94 (d, *J* = 10.2 Hz, 1.01H, α-C1 isomer), 5.85 (d, *J* = 10.3 Hz, 0.39H, β-C1 isomer), 5.77 (d, *J* = 10.3 Hz, 1.02H, α-C1 isomer), 5.56 (s, 1.00H, α-C1 isomer), 5.32 (s, 0.92H, α-C1 isomer), 5.30 (s, 0.41H, β-C1 isomer), 5.25 (d, *J* = 6.7 Hz, 0.41H, β-C1 isomer), 5.18–5.13 (m, 1.47H, β-C3 isomer), 5.08 (dd, *J* = 9.4, 4.7 Hz, 1.68H, α-C3 isomer), 4.88 (q, *J* = 5.1 Hz, 1.66H, α-C3 isomer), 4.82–4.77 (m, 1.46H, β-C3 isomer), 4.44–3.94 (m, 18.43H, four isomers), 2.16–2.02 (m, 28.96H, four isomers); ^1^^3^C{^1^H} NMR (125 MHz, CDCl_3_) δ 170.9, 170.8, 170.7, 170.3, 169.8, 169.5, 147.1 (α-C3 isomer), 145.9 (β-C3 isomer), 129.8 (α-C1 isomer), 128.7 (β-C1 isomer), 128.1 (β-C1 isomer), 126.4 (α-C1 isomer), 98.2 (β-C3 isomer), 96.6 (α-C3 isomer), 84.5 (β-C1 isomer), 84.4 (α-C1 isomer), 74.4 (β-C3 isomer), 74.0 (β-C1 isomer), 70.7 (α-C3 isomer), 68.9 (α-C1 isomer), 68.1 (α-C3 isomer), 67.6 (β-C3 isomer), 64.6 (α-C1 isomer), 64.0 (β-C1 isomer), 63.0 (β-C1 isomer), 62.6 (α-C1 isomer), 61.9 (α-C3 isomer), 61.6 (β-C3 isomer), 57.8 (β-C3 isomer), 53.4 (α-C3 isomer), 21.0, 20.9, 20.8, 20.8 20.7. The spectroscopic data agree with the values reported in ref (34).

#### [(2*R*,3*S*)-3-Acetoxy-6-(propa-1,2-dien-1-yl)-3,6-dihydro-2*H*-pyran-2-yl]methyl Acetate (**9**)

Compound **9** was prepared following the general procedure using compound **4** (30 mg, 0.110 mmol, 1.0 equiv) as the starting material, trimethyl(propargyl)silane as the nucleophile (0.11 mL, 0.771 mmol, 7.0 equiv), and **1**·BF_4_ (53.5 mg, 0.220 mmol, 2.0 equiv). The crude reaction mixture was purified by flash column chromatography (9:1 hexanes/EtOAc) to give **9** (11.12 mg, 40% yield) as a colorless oil: ^1^H NMR (500 MHz, CDCl_3_) δ 5.90 (d, *J* = 9.3 Hz, 1H), 5.81 (d, *J* = 10.5 Hz, 1H), 5.31–5.29 (m, 1H), 5.26 (d, *J* = 8.6 Hz, 1H), 4.90–4.83 (m, 3H), 4.23–4.16 (m, 2H), 3.95–3.88 (m, 1H), 2.10 (s, 3H), 2.09 (s, 3H); ^1^^3^C{^1^H} NMR (125 MHz, CDCl_3_) δ 209.3, 171.1, 170.5, 130.9, 125.3, 89.7, 77.4, 70.7, 69.0, 65.2, 63.3, 21.2, 21.0.^35^

#### [(2*R*,3*S*)-3-Acetoxy-6-(furan-2-yl)-3,6-dihydro-2*H*-pyran-2-yl]methyl Acetate (**10a**) and [(2*R*,3*S*,4*S*)-3-Acetoxy-4-(furan-2-yl)-3,4-dihydro-2*H*-pyran-2-yl]methyl Acetate (**10b**)

Compound **10** was prepared following the general procedure using compound **4** (30 mg, 0.110 mmol, 1.0 equiv) as the starting material, furan as the nucleophile (0.05 mL, 0.771 mmol, 7.0 equiv), and **1**·BF_4_ (53.5 mg, 0.220 mmol, 2.0 equiv). The crude reaction mixture was purified by flash column chromatography (95:5 hexanes/EtOAc) to give **10** (19.4 mg, 63% yield) as a yellowish oil. Only **10a** (29% yield): ^1^H NMR (500 MHz, CDCl_3_) δ 7.45 (s, 1H), 6.36 (d, *J* = 3.1 Hz, 1H), 6.34 (d, *J* = 2.7 Hz, 1H), 6.04 (s, 1H), 5.96 (d, *J* = 10.4 Hz, 1H), 5.37–5.32 (m, 2H), 4.24–4.17 (m, 1H), 4.11 (d, *J* = 12.0 Hz, 1H), 3.84 (td, *J* = 6.1, 3.3 Hz, 1H), 2.09 (s, 3H), 2.06 (s, 3H); ^1^^3^C{^1^H} NMR (125 MHz, CDCl_3_) δ 171.1, 170.5, 151.3, 143.5, 128.3, 127.2, 110.3, 110.1, 68.9, 68.5, 65.0, 63.1, 21.2, 20.9. Only **10b** (34% yield): ^1^H NMR (500 MHz, CDCl_3_) δ 7.37 (d, *J* = 1.8 Hz, 1H), 6.52 (dd, *J* = 6.0, 1.6 Hz, 1H), 6.33 (dd, *J* = 3.2, 1.8 Hz, 1H), 6.13 (d, *J* = 3.2 Hz, 1H), 5.05 (dd, *J* = 9.9, 6.1 Hz, 1H), 4.81 (t, *J* = 5.6 Hz, 1H), 4.35–4.25 (m, 2H), 4.14 (ddd, *J* = 9.9, 4.5, 2.8 Hz, 1H), 4.05–3.99 (m, 1H), 2.09 (s, 3H), 1.98 (s, 3H); ^1^^3^C{^1^H} NMR (125 MHz, CDCl_3_) δ 170.9, 170.1, 153.5, 144.1, 142.6, 110.4, 109.2, 98.5, 70.9, 67.8, 62.7, 33.9, 20.9, 20.9. The spectroscopic data agree with the values reported in ref (36).

#### [(2*R*,3*R*)-3-Acetoxy-6-allyl-3,6-dihydro-2*H*-pyran-2-yl]methyl Acetate (**12**)

Compound **12** was prepared following the general procedure using compound **11** (30 mg, 0.110 mmol, 1.0 equiv) as the starting material, ATMS as the nucleophile (0.12 mL, 0.771 mmol, 7.0 equiv), and **1**·BF_4_ (53.5 mg, 0.220 mmol, 2.0 equiv). The crude reaction mixture was purified by flash column chromatography (9:1 hexanes/EtOAc) to give **12** (24.3 mg, 87% yield) as a colorless oil. Only α isomer: ^1^H NMR (500 MHz, CDCl_3_) δ 6.03 (d, *J* = 10.5 Hz, 1H), 6.00–5.93 (m, 1H), 5.88–5.77 (m, 1H), 5.17–5.00 (m, 3H), 4.34 (tt, *J* = 5.9, 3.0 Hz, 1H), 4.24–4.15 (m, 2H), 4.15–4.07 (m, 1H), 2.42 (dt, *J* = 15.8, 7.9 Hz, 1H), 2.27 (dt, *J* = 14.7, 6.8 Hz, 1H), 2.06 (s, 3H), 2.05 (d, *J* = 2.7 Hz, 3H); ^1^^3^C NMR (125 MHz, CDCl_3_) δ 170.9, 170.7, 134.9, 134.0, 122.1, 117.8, 72.4, 68.1, 63.9, 63.1, 36.9, 21.0, 20.9. The spectroscopic data agree with the values reported in ref (34).

#### [(2*R*,3*R*)-3-Acetoxy-6-cyano-3,6-dihydro-2*H*-pyran-2-yl]methyl Acetate (**13**)

Compound **13** was prepared following the general procedure using compound **11** (30 mg, 0.110 mmol, 1.0 equiv) as the starting material, TMSCN as the nucleophile (0.07 mL, 0.550 mmol, 5.0 equiv), and **1**·BF_4_ (53.5 mg, 0.220 mmol, 2.0 equiv). The crude reaction mixture was purified by flash column chromatography (9:1 hexanes/EtOAc) to give **13** (9 mg, 34% yield). Major α isomer: yellowish solid; mp 114–115 °C; 28.9% yield; ^1^H NMR (500 MHz, CDCl_3_) δ 6.27 (ddd, *J* = 9.6, 6.3, 2.8 Hz, 1H), 6.05 (dt, *J* = 10.2, 3.4 Hz, 1H), 5.17–5.12 (m, 2H), 4.29–4.21 (m, 3H), 2.09 (d, *J* = 2.3 Hz, 3H), 2.08 (d, *J* = 2.7 Hz, 3H); ^1^^3^C{^1^H} NMR (125 MHz, CDCl_3_) δ 170.8, 170.2, 126.6, 126.5, 115.4, 72.0, 62.7, 62.3, 62.3, 20.9, 20.8. Minor β isomer: yellow oil; 5.1% yield; ^1^H NMR (500 MHz, CDCl_3_) δ 6.23 (ddd, *J* = 10.1, 5.5, 2.5 Hz, 1H), 6.03 (dd, *J* = 10.1, 1.7 Hz, 1H), 5.08 (d, *J* = 2.8 Hz, 1H), 5.06 (t, *J* = 2.2 Hz, 1H), 4.24–4.19 (m, 2H), 3.93 (ddd, *J* = 7.5, 5.7, 2.4 Hz, 1H), 2.09 (s, 3H), 2.07 (s, 3H); ^1^^3^C{^1^H} NMR (125 MHz, CDCl_3_) δ 170.7, 170.4, 126.9, 126.2, 115.7, 74.0, 63.5, 62.5, 62.2, 20.8. The spectroscopic data agree with the values reported in refs (34) and (37).

#### [(2*R*,3*R*)-3-Acetoxy-6-azido-3,6-dihydro-2*H*-pyran-2-yl]methyl Acetate (**14a**) and [(2*R*,3*R*)-3-Acetoxy-4-azido-3,4-dihydro-2*H*-pyran-2-yl]methyl Acetate (**14b**)

Compound **14** was prepared following the general procedure using compound **11** (30 mg, 0.110 mmol, 1.0 equiv) as the starting material, TMSN_3_ as the nucleophile (0.03 mL, 0.220 mmol, 2.0 equiv), and **1**·BF_4_ (53.5 mg, 0.220 mmol, 2.0 equiv). The crude reaction mixture was purified by flash column chromatography (9:1 hexanes/EtOAc) to give **14** (23.9 mg, 85% yield) as a colorless oil: ^1^H NMR (500 MHz, CDCl_3_) δ 6.71–6.66 (m, 1H, α-C3 isomer), 6.53 (dd, *J* = 6.2, 2.0 Hz, 17H, β-C3 isomer), 6.22 (ddd, *J* = 10.1, 5.3, 1.6 Hz, 0.07H, β-C1 isomer), 6.17 (ddd, *J* = 10.0, 5.6, 1.4 Hz, 0.35H, α-C1 isomer), 5.95 (dd, *J* = 10.0, 3.2 Hz, 0.35H, α-C1 isomer), 5.93–5.90 (m, 0.08H, β-C1 isomer), 5.61 (dd, *J* = 3.3, 1.4 Hz, 0.35H, α-C1 isomer), 5.42 (dt, *J* = 4.7, 1.6 Hz, 0.18H, β-C3 isomer), 5.20 (d, *J* = 1.6 Hz, 0.08H, β-C1 isomer), 5.09 (dt, *J* = 4.6, 2.1 Hz, 0.09H, β-C1 isomer), 5.04 (dd, *J* = 5.6, 2.5 Hz, 0.36H, α-C1 isomer), 4.94–4.88 (m, 2H, α-C3 isomer), 4.79 (ddd, *J* = 6.3, 2.4, 1.6 Hz, 0.18H, β-C3 isomer), 4.34 (ddd, *J* = 7.4, 5.3, 2.5 Hz, 0.38H, α-C1 isomer), 4.27–4.24 (m, 0.17H), 4.24–4.19 (m, 2.36H, four isomers), 4.17–4.13 (m, 1H, α-C3 isomer), 4.11–4.09 (m, 0.17H, β-C3 isomer), 4.06 (d, *J* = 2.7 Hz, 0.08H, β-C1 isomer), 3.81 (dd, *J* = 4.9, 2.5 Hz, 1H, α-C3 isomer), 2.11–2.04 (m, 8.76H, four isomers); ^13^C{^1^H} NMR (125 MHz, CDCl_3_) δ 170.6, 170.4, 170.1, 170.0, 169.7 (four isomers), 147.5, 146.1 (β-C3 isomer), 130.3 (β-C1 isomer), 129.1 (α-C1 isomer), 126.9 (β-C1 isomer), 125.6 (α-C1 isomer), 97.5 (β-C3 isomer), 94.9 (α-C3 isomer), 84.8, 84.0 (α-C1 isomer), 73.2 (β-C3 isomer), 72.5, 70.2 (α-C3 isomer), 68.7, 67.2, 67.1, 65.2 (β-C3 isomer), 62.9 (β-C1 isomer), 62.3 (α-C1 isomer), 62.2 (α-C3 isomer), 62.1 (β-C3 isomer and α-C1 isomer), 53.4, 52.6 (α-C3 isomer), 20.6 (four isomers). The spectroscopic data agree with the values reported in refs (19) and (36d).

#### (3*S*)-6-Allyl-3,6-dihydro-2*H*-pyran-3-yl Acetate (**16**)

Compound **16** was prepared following the general procedure using compound **15** (30 mg, 0.150 mmol, 1.0 equiv) as the starting material, ATMS as the nucleophile (0.16 mL, 1.050 mmol, 7.0 equiv), and **1**·BF_4_ (18.2 mg, 0.075 mmol, 0.5 equiv). The crude reaction mixture was purified by flash column chromatography (95:5 hexanes/EtOAc) to give **16** (20.7 mg, 76% yield) as a colorless oil. Major α isomer: ^1^H NMR (500 MHz, CDCl_3_) δ 5.90 (d, *J* = 10.5 Hz, 1H), 5.87–5.75 (m, 2H), 5.26–5.20 (m, 1H), 5.17–5.04 (m, 2H), 4.21–4.14 (m, 1H), 4.14–4.07 (m, 1H), 3.57–3.49 (m, 1H), 2.39–2.23 (m, 2H), 2.06 (s, 3H); ^1^^3^C{^1^H} NMR (125 MHz, CDCl_3_) δ 170.7, 134.0, 133.6, 124.5, 117.7, 73.2, 65.2, 65.0, 38.7, 21.2. Minor β isomer (traces): ^1^H NMR (500 MHz, CDCl_3_) δ 6.01 (d, *J* = 10.3 Hz, 1H), 5.94 (d, *J* = 10.9 Hz, 1H), 5.90–5.80 (m, 1H), 5.19–5.08 (m, 2H), 5.01–4.98 (m, 1H), 4.14–4.04 (m, 2H), 3.76 (dd, *J* = 13.0, 3.0 Hz, 1H), 2.43 (dt, *J* = 14.2, 7.1 Hz, 1H), 2.33 (dt, *J* = 14.0, 6.5 Hz, 1H), 2.09 (s, 3H). The spectroscopic data agree with the values reported in refs (34) and (36d).

#### (3*S*)-6-Cyano-3,6-dihydro-2*H*-pyran-3-yl Acetate (**17**)

Compound **17** was prepared following the general procedure using compound **15** (30 mg, 0.150 mmol, 1.0 equiv) as the starting material, ATMCN as the nucleophile (0.04 mL, 0.300 mmol, 2.0 equiv), and **1**·BF_4_ (18.2 mg, 0.075 mmol, 0.5 equiv). The crude reaction mixture was purified by flash column chromatography (95:5 hexanes/EtOAc) to give **17** (15.0 mg, 60% yield) as a colorless oil: ^1^H NMR (500 MHz, CDCl_3_) δ 6.19 (dddd, *J* = 10.2, 5.3, 2.1, 1.0 Hz, 1H, α isomer), 6.11–6.07 (m, 0.41H, β isomer), 6.03 (ddd, *J* = 10.0, 3.9, 0.9 Hz, 1H, α isomer), 5.92 (ddd, *J* = 10.2, 3.0, 1.6 Hz, 0.42H, β isomer), 5.29–5.21 (m, 0.42H, β isomer), 5.08–5.01 (m, 2H, α isomer), 4.97–4.91 (m, 0.41H, β isomer), 4.08–4.05 (m, 2H, α isomer), 4.03 (dd, *J* = 5.1, 0.8 Hz, 0.42H, β isomer), 3.80 (ddd, *J* = 11.8, 6.7, 0.5 Hz, 0.43H, β isomer), 2.08 (s, 1.2H, β isomer), 2.08 (s, 3H, α isomer); ^1^^3^C{^1^H} NMR (125 MHz, CDCl_3_) δ 170.5 (both isomers), 128.8 (β isomer), 126.7 (α isomer), 126.2 (α isomer), 125.1 (β isomer), 116.0 (β isomer), 115.6 (α isomer), 65.7 (α isomer), 64.9 (β isomer), 63.3 (β isomer), 62.6 (α isomer), 62.4 (β isomer), 61.8 (α isomer), 21.1 (α isomer), 21.0 (β isomer). The spectroscopic data agree with the values reported in refs (34) and (36d).

#### (3*S*)-6-Azido-3,6-dihydro-2*H*-pyran-3-yl Acetate (**18a**) and (3*S*)-4-azido-3,4-dihydro-2*H*-pyran-3-yl Acetate (**18b**)

Compound **18** was prepared following the general procedure using compound **15** (30 mg, 0.150 mmol, 1.0 equiv) as the starting material, ATMN_3_ as the nucleophile (0.04 mL, 0.300 mmol, 2.0 equiv), and **1**·BF_4_ (18.2 mg, 0.075 mmol, 0.5 equiv). The crude reaction mixture was purified by flash column chromatography (95:5 hexanes/EtOAc) to give **18** (21.9 mg, 80% yield) as a colorless oil: ^1^H NMR (500 MHz, CDCl_3_) δ 6.72 (d, *J* = 5.9 Hz, 1H, α-C3 isomer), 6.59 (d, *J* = 5.8 Hz, 0.35H, β-C3 isomer), 6.21 (dd, *J* = 10.4, 5.0 Hz, 0.30H, α-C1 isomer), 6.12 (d, *J* = 10.1 Hz, 0.13H, β-C1 isomer), 6.03 (d, *J* = 9.8 Hz, 0.29H, α-C1 isomer), 5.90 (d, *J* = 10.2 Hz, 0.11H, β-C1 isomer), 5.60–5.57 (m, 0.28H, α-C1 isomer), 5.42–5.38 (m, 0.11H, β-C1 isomer), 5.34–5.29 (m, 0.12H, β-C1 isomer), 5.25–5.17 (m, 0.40H, β-C3 isomer), 5.10–5.03 (m, 0.33H, α-C1 isomer), 4.99–4.87 (m, 2.48H), 4.28–4.14 (m, 1.86H), 4.10–3.99 (m, 2.29H), 3.89–3.85 (m, 1.03H, α-C3 isomer), 2.22 (s, 1.29H), 2.16 (s, 3.97H); ^1^^3^C{^1^H} NMR (125 MHz, CDCl_3_) δ 169.4 (β-C1 isomer), 168.9 (β-C3 isomer), 168.8 (α-C3 isomer), 146.9 (α-C3 isomer), 146.5 (β-C3 isomer), 128.3 (α-C1 isomer), 128.0 (β-C1 isomer), 127.0 (β-C1 isomer), 124.7 (α-C1 isomer), 95.3, 94.1 (α-C3 isomer), 83.2 (β-C1 isomer), 82.6 (α-C1 isomer), 66.9, 66.7 (β-C3 isomer), 62.5 (α-C3 isomer), 62.1 (β-C3 isomer), 61.9 (both C1 isomers), 61.6 (α-C1 isomer), 61.5 (β-C1 isomer), 52.0 (β-C3 isomer), 51.7 (α-C3 isomer), 20.0 (β-C1 isomer), 19.9 (β-C3 isomer), 19.7 (α-C1 isomer). The spectroscopic data agree with the values reported in ref (38).

### Synthesis of 1-{[(2*S*,3*R*)-2-Azidotetrahydro-2*H*-pyran-3-yl]oxy}-2,2,6,6-tetramethylpiperidine (*cis*-**21**) and 1-{[(2*S*,3*S*)-2-Azidotetrahydro-2*H*-pyran-3-yl]oxy}-2,2,6,6-tetramethylpiperidine (*trans*-**21**)

3,4-Dihydro-2*H*-pyran (92.8 mg, 0.10 mL, 1.103 mmol, 1.0 equiv) was added to a solution of TEMPO^+^BF_4_^–^ (402.2 mg, 1.654 mmol, 1.5 equiv) in anhydrous CH_3_CN (5.5 mL, 0.20 M) in a flame-dried flask under a nitrogen atmosphere. Subsequently, TMSN_3_ (120.4 mg, 0.15 mL, 1.213 mmol, 1.1 equiv) was added dropwise to the solution, and the reaction mixture was stirred overnight at room temperature. Upon completion of the reaction as indicated by TLC, the solvent was removed under a vacuum, and the residue was purified via flash chromatography (9:1 hexane/EtOAc) to give **21** (53 mg, 17%, 1:1 *cis*/*trans* mixture) as a colorless oil. *cis*-**21a**: ^1^H NMR (500 MHz, CDCl_3_) δ 5.35 (d, *J* = 3.1 Hz, 1H), 3.90–3.85 (m, 1H), 3.68 (ddt, *J* = 10.9, 3.8, 2.0 Hz, 1H), 3.60 (dt, *J* = 4.5, 3.0 Hz, 1H), 1.95–1.88 (m, 2H), 1.81–1.74 (m, 1H), 1.49–1.36 (m, 6H), 1.35–1.28 (m, 1H), 1.23 (s, 3H), 1.13 (s, 9H); ^1^^3^C{^1^H} NMR (125 MHz, CDCl_3_) δ 88.7, 76.9, 63.0, 60.5, 60.2, 40.5, 40.4, 34.6, 34.1, 24.4, 21.3, 20.4, 17.2; HRMS-FAB *m*/*z* [M + H]^+^ calcd for C_14_H_27_N_4_O_2_ 283.2134, found 283.2148. *trans*-**21b**: ^1^H NMR (500 MHz, CDCl_3_) δ 5.46 (d, *J* = 3.6 Hz, 1H), 3.87 (dt, *J* = 11.4, 3.9 Hz, 1H), 3.72 (td, *J* = 11.6, 3.1 Hz, 1H), 3.60 (ddt, *J* = 11.2, 4.5, 1.5 Hz, 1H), 2.12–2.05 (m, 1H), 1.75–1.55 (m, 4H), 1.50–1.42 (m, 4H), 1.24–1.28 (m, 1H), 1.20 (s, 3H), 1.14 (s, 6H), 1.10 (s, 3H); ^1^^3^C{^1^H} NMR (125 MHz, CDCl_3_) δ 89.1, 80.2, 61.1, 60.2, 60.0, 40.4, 34.3, 34.1, 25.2, 24.6, 20.3, 17.2; HRMS-FAB *m*/*z* [M + H]^+^ calcd for C_14_H_27_N_4_O_2_ 283.2134, found 283.2132.

## Data Availability

The data underlying this study are available in the published article and its Supporting Information.

## Supporting Information Available

^1^H and ^13^C{^1^H} spectra of new compounds; *XYZ* coordinates for optimized geometries **4**-^4^H_5_, **4**-^5^H_4_, **MC1** (**4**-^4^H_5_-**1**), and **MC2** (**4**-^5^H_4_-**1**) in a vacuum and acetonitrile at the M06-2X/6-311+G** level of theory; and complexation energies of **MC1** and **MC2** (PDF)
