# Supplementary material for: Ferrier Glycosylation Mediated by the TEMPO Oxoammonium Cation
Source: J Org Chem. 2024 Aug 5;89(16):11281–92. doi: 10.1021/acs.joc.4c00978 (PMC11334189; doi:10.1021/acs.joc.4c00978)
Supplement: Supplementary file 1 — jo4c00978_si_001.pdf [file jo4c00978_si_001.pdf]

Supporting information

Ferrier Glycosylation Mediated by TEMPO Oxoammonium Cation

Luis F. Porras-Santos,[1] Jacinto Sandoval-Lira,[2] Julio M. Hernández-Pérez,[1] Leticia Quintero,[1] Pedro López-Mendoza[1,] and Fernando Sartillo-Piscil [1,*]*

[1]Centro de Investigación de la Facultad de Ciencias Químicas, Benemérita Universidad Autónoma de Puebla (BUAP), 14 Sur Esq. San Claudio, Col. San Manuel, 72570, Puebla, México.

[2]Departamento de Ciencias Básicas, TecNM campus Instituto Tecnológico Superior de San Martín Texmelucan, Camino a la Barranca de Pesos, 74120, San Martín Texmelucan, Puebla, México

fernando.sartillo@correo.buap.mx, pedro.lopez@viep.com.mx

Fax: +52222 2454972; Tel: +52 222 2955500 ext. 7391.

Content

1. **Scaling the TEMPO$^+$-mediated *C*-glycosylation.** S4
2. Temperature-Time Profile for Synthesis of the *C*-allylglycosides 6. .... S5
3. **Characterization Data** S6
Figure S3. ^{1}H NMR spectrum of compound **4** (500 MHz, $CDCl_3$). S6
Figure S5. ^{1}H NMR spectrum of compound α-**6** (500 MHz, $CDCl_3$). S8
Figure S6. $^{13}C\{^1H\}$ NMR spectrum of compound α-**6** (125 MHz, $CDCl_3$). S9
Figure S7. ^{1}H NMR spectrum of compound α-**7** (500 MHz, $CDCl_3$). S10
Figure S8. $^{13}C\{^1H\}$ NMR spectrum of compound α-**7** (125 MHz, $CDCl_3$). S11
Figure S9. ^{1}H NMR spectrum of compound β-**7** (500 MHz, $CDCl_3$). S12
Figure S10. $^{13}C\{^1H\}$ NMR spectrum of compound β-**7** (125 MHz, $CDCl_3$). S13
Figure S11. ^{1}H NMR spectrum of mixture **8a** + **8b** (500 MHz, $CDCl_3$). S14
Figure S12. $^{13}C\{^1H\}$ NMR spectrum of mixture **8a** + **8b** (125 MHz, $CDCl_3$). S15
Figure S13. ^{1}H NMR spectrum of compound **9** (500 MHz, $CDCl_3$). S16
Figure S14. $^{13}C\{^1H\}$ NMR spectrum of compound **9** (125 MHz, $CDCl_3$) S17
Figure S15. ^{1}H NMR spectrum of compound **10a** (500 MHz, $CDCl_3$). S18
Figure S16. $^{13}C\{^1H\}$ NMR spectrum of compound **10a** (125 MHz, $CDCl_3$) S19
Figure S17. ^{1}H NMR spectrum of compound **10b** (500 MHz, $CDCl_3$) S20
Figure S18. $^{13}C\{^1H\}$ NMR spectrum of compound **10b** (125 MHz, $CDCl_3$). S21
Figure S19. ^{1}H NMR spectrum of compound **11** (500 MHz, $CDCl_3$). S22
Figure S20. $^{13}C\{^1H\}$ NMR spectrum of compound **11** (125 MHz, $CDCl_3$). S23
Figure S21. ^{1}H NMR spectrum of compound **12** (500 MHz, $CDCl_3$). S24
Figure S22. $^{13}C\{^1H\}$ NMR spectrum of compound **12** (125 MHz, $CDCl_3$) S25
Figure S23. ^{1}H NMR spectrum of compound α-**13** (500 MHz, $CDCl_3$). S26
Figure S24. $^{13}C\{^1H\}$ NMR spectrum of compound α-**13** (125 MHz, $CDCl_3$). S27
Figure S25. ^{1}H NMR spectrum of compound β-**13** (500 MHz, CDCl3). S28
Figure S26. $^{13}C\{^1H\}$ NMR spectrum of compound β-**13** (125 MHz, $CDCl_3$). S29
Figure S27. ^{1}H NMR spectrum of mixture **14a** + **14b** (500 MHz, $CDCl_3$). S30
Figure S28. $^{13}C\{^1H\}$ NMR spectrum of mixture **14a** + **14b** (125 MHz, $CDCl_3$). S31
Figure S29. ^{1}H NMR spectrum of **15** (500 MHz, $CDCl_3$). S32
Figure S30. $^{13}C\{^1H\}$ NMR spectrum of **15** (125 MHz, $CDCl_3$). S33
Figure S31. ^{1}H NMR spectrum of α-**16** (500 MHz, $CDCl_3$). S34

Figure S32. $^{13}C\{^1H\}$ NMR spectrum of α-**16** (125 MHz, $CDCl_3$)....................................S35
Figure S33. ^1H NMR spectrum of mixture α-**17** + β-**17** (500 MHz, $CDCl_3$)....................S36
Figure S34. $^{13}C\{^1H\}$ NMR spectrum of mixture α-**17** + β-**17** (125 MHz, $CDCl_3$)...........S37
Figure S35. ^1H NMR spectrum of mixture **18a** + **18b** (500 MHz, $CDCl_3$).......................S38
Figure S36. $^{13}C\{^1H\}$ NMR spectrum of **18a** + **18b** (125 MHz, $CDCl_3$)..........................S39
Figure S37. ^1H NMR spectrum of compound *cis*-**21** (500 MHz, $CDCl_3$).........................S40
Figure S38. $^{13}C\{^1H\}$ NMR of compound *cis*-**21** (125 MHz, $CDCl_3$). S41
Figure S39. HSQC NMR of compound *cis*-**21** (500 MHz, $CDCl_3$). S42
Figure S40. ^1H NMR spectrum of compound *trans*-**21** (500 MHz, $CDCl_3$). S43
Figure S41. $^{13}C\{^1H\}$ NMR spectrum of compound *trans*-**21** (125 MHz, $CDCl_3$)............S44
Figure S42. HSQC NMR of compound *trans*-**21** (500 MHz, $CDCl_3$). S45
4. Optimized Geometries and Complexation Energies S46
Table S1. XYZ coordinates of structure **4-$^4H_5$** in vacuum. S46
Table S2. XYZ coordinates of structure **4-$^5H_4$** in vacuum. S47
Table S3. XYZ coordinates of structure MC1 (**4-$^4H_5$-1**) in vacuum. S49
Table S4. XYZ coordinates of structure MC2 (**4-$^5H_4$-1**) in vacuum. S51
Table S5. XYZ coordinates of structure **4-$^4H_5$** in acetonitrile. S54
Table S6. XYZ coordinates of structure **4-$^5H_4$** in acetonitrile. S55
Table S7. XYZ coordinates of structure **MC1** (**4-$^4H_5$-1**) in acetonitrile. S57
Table S8. XYZ coordinates of structure **MC2** (**4-$^5H_4$-1**) in acetonitrile. S59
Table S9. Complexation Energy and BSSE Corrected Complexation Energy..................S62

1. Scaling the TEMPO$^+$-mediated *C*-glycosylation.

TEMPO$^+$BF$_4^-$ (1.215 g, 5.0 mmol, 2.0 equivalents) and tri-*O*-acetyl-*D*-glucal (680.6 mg, 2.5 mmol, 1.0 equivalents) were dissolved in anhydrous CH_3CN (5 ml, 0.5 M) under a nitrogen atmosphere in a flame-dried sealed tube. Then, ATMS (2.78 ml, 17.5 mmol, 7.0 equivalents) was added to the solution at room temperature. The reaction mixture was stirred and heated in a microwave reactor at 70 °C for 20 min using 70 W of power. Upon completion of the reaction time as confirmed by TLC, the solvent was removed under vacuum, and the residue was purified via flash column chromatography on silica gel (Hexane:EtOAc, 9:1) to give the diastereomeric mixture of C-allyglicosides 6 (508 mg, 79.9% yield) as a colorless oil.

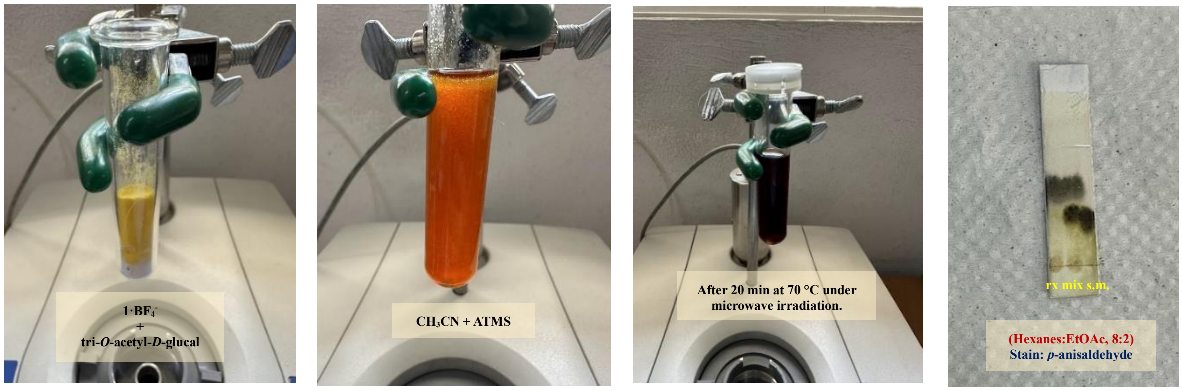

Figure S1. Scale-up process for the TEMPO$^+$-mediated synthesis of the *C*-allylglycoside **6** (from left to right).

2. Temperature-Time Profile for Synthesis of the *C*-allylglycosides 6.

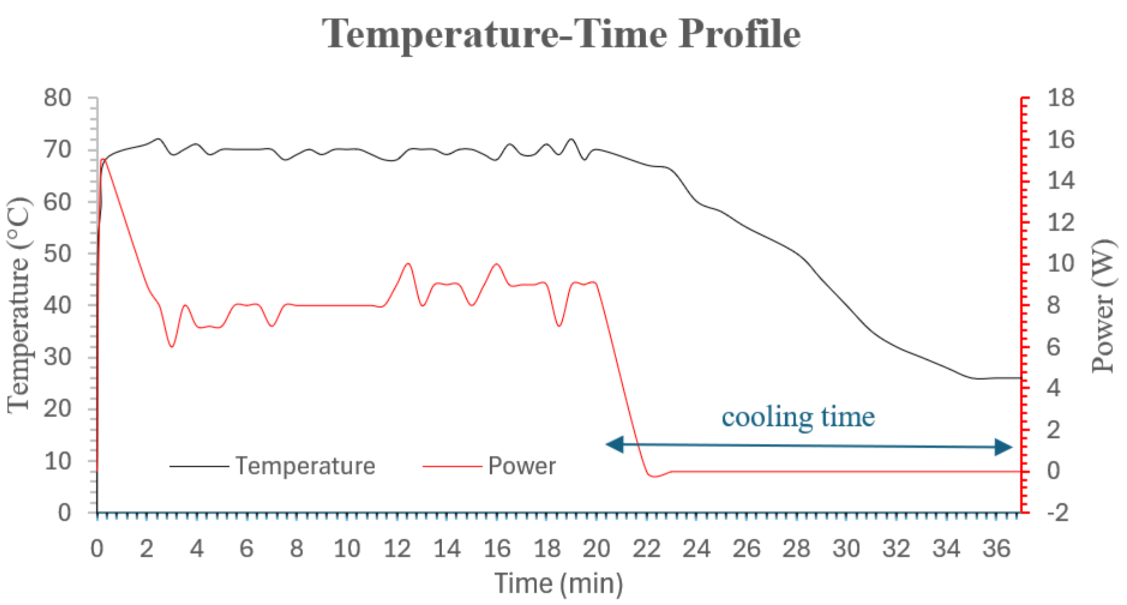

Figure S2. Temperature-Time profile for the synthesis of the *C*-allylglycoside **6**. Target temperature = 70 °C, run time = 30 s, hold Time = 20 min, experiment scale of starting material = 0.11 mmol.

3. Characterization Data

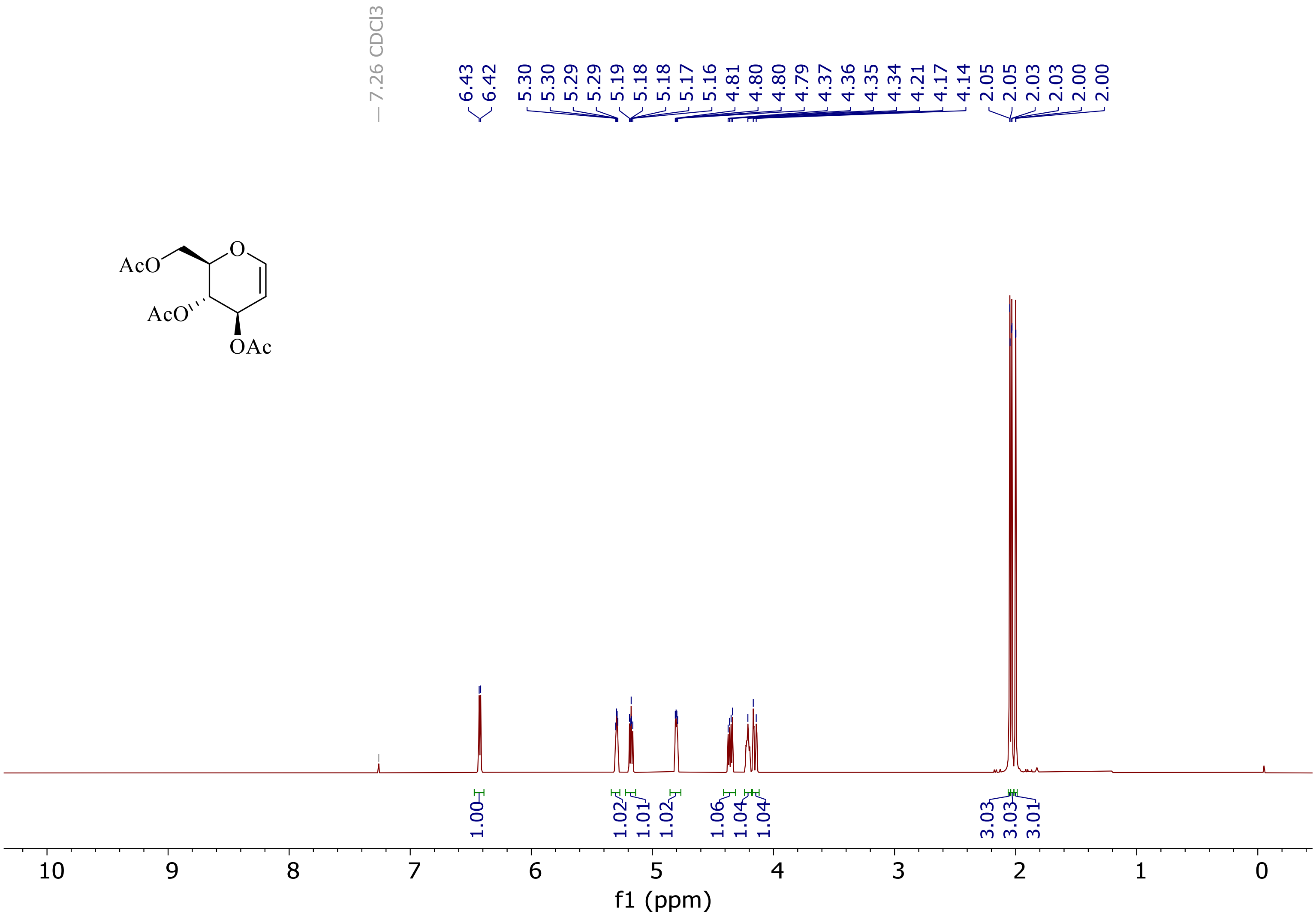

Figure S3. ^{1}H NMR spectrum of compound **4** (500 MHz, $CDCl_3$).

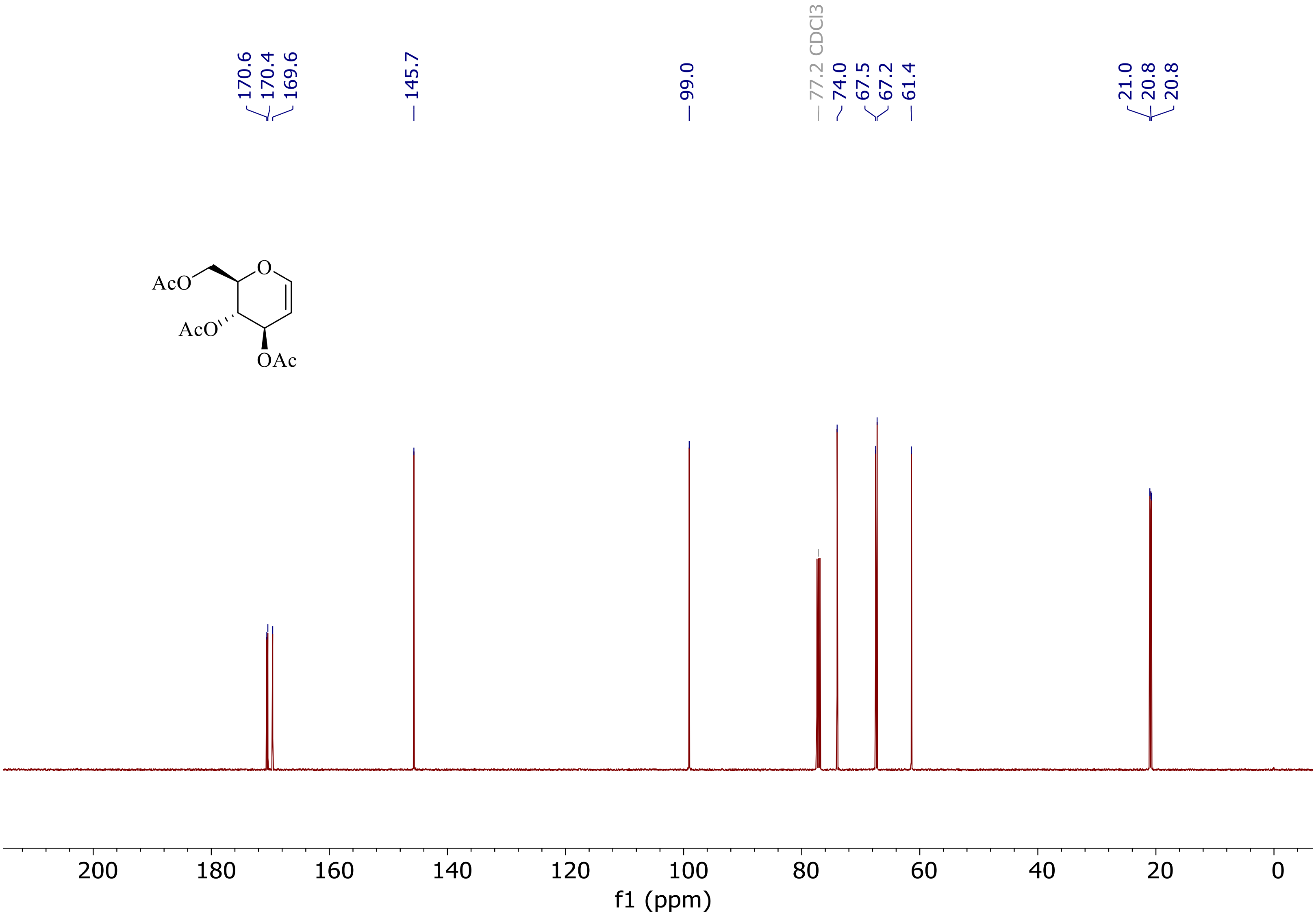

Figure S4. $^{13}C\{^{1}H\}$ NMR spectrum of compound **4** (125 MHz, $CDCl_3$).

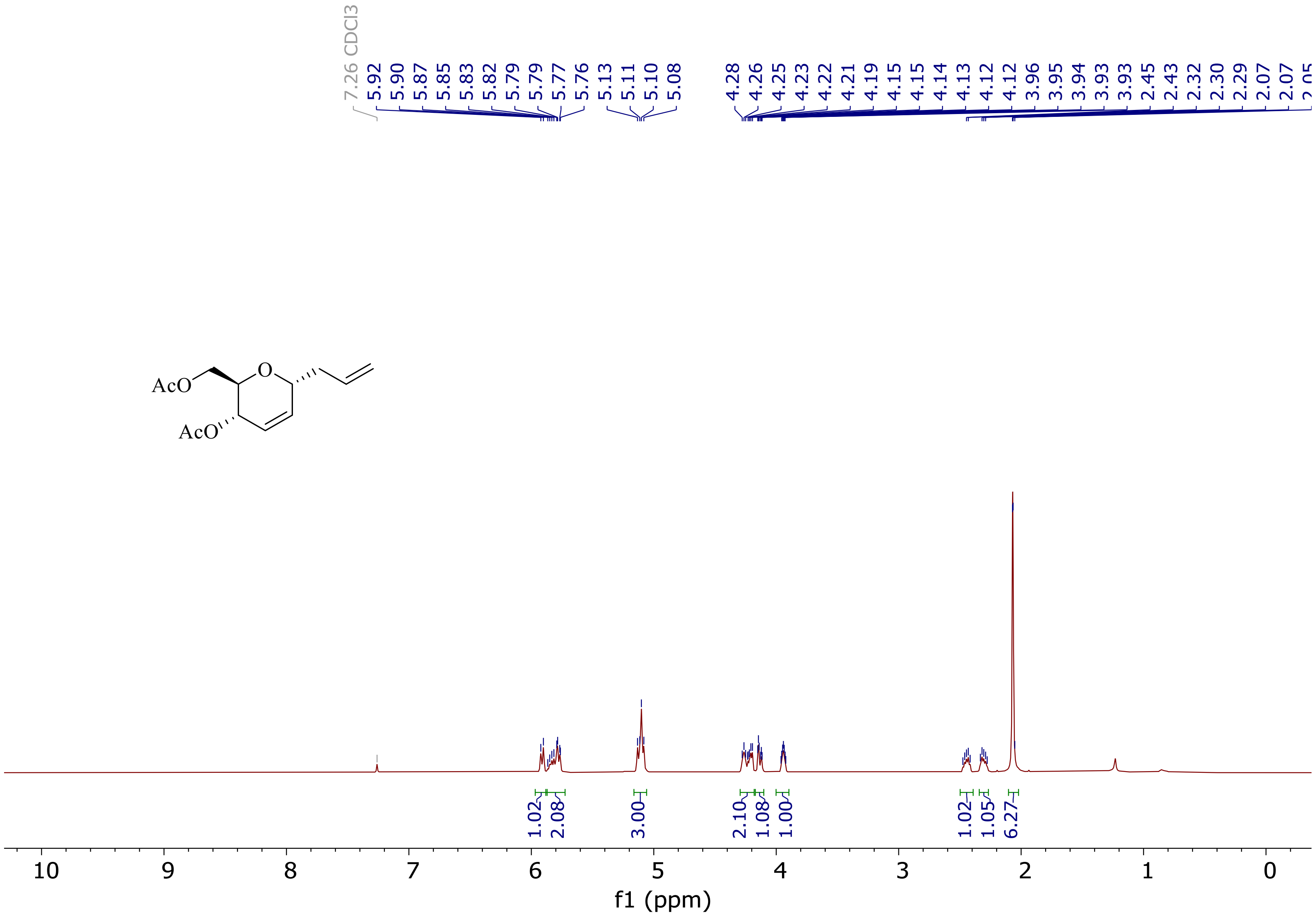

Figure S5. ^{1}H NMR spectrum of compound α-**6** (500 MHz, $CDCl_3$).

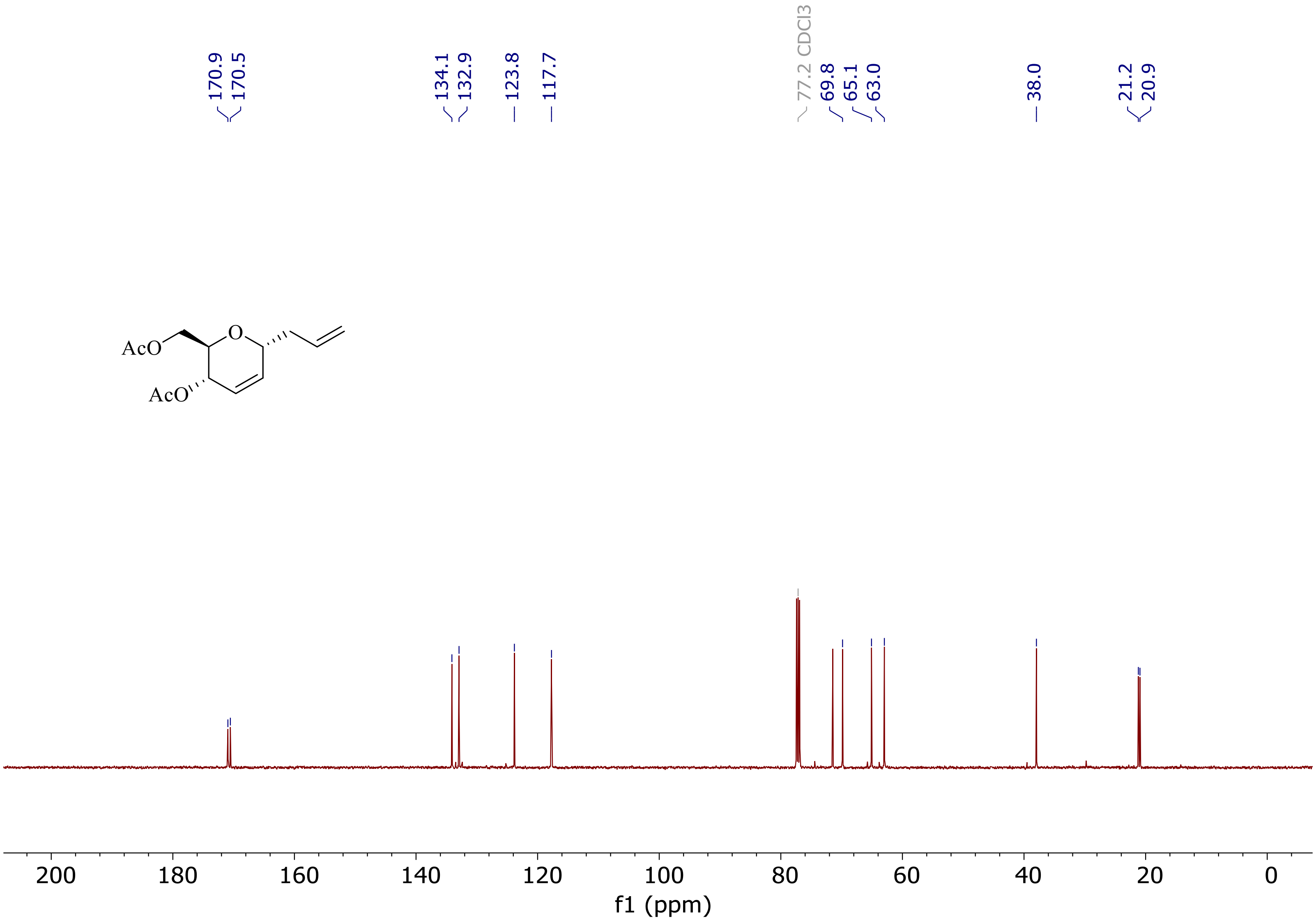

Figure S6. $^{13}C\{^{1}H\}$ NMR spectrum of compound **α-6** (125 MHz, $CDCl_3$).

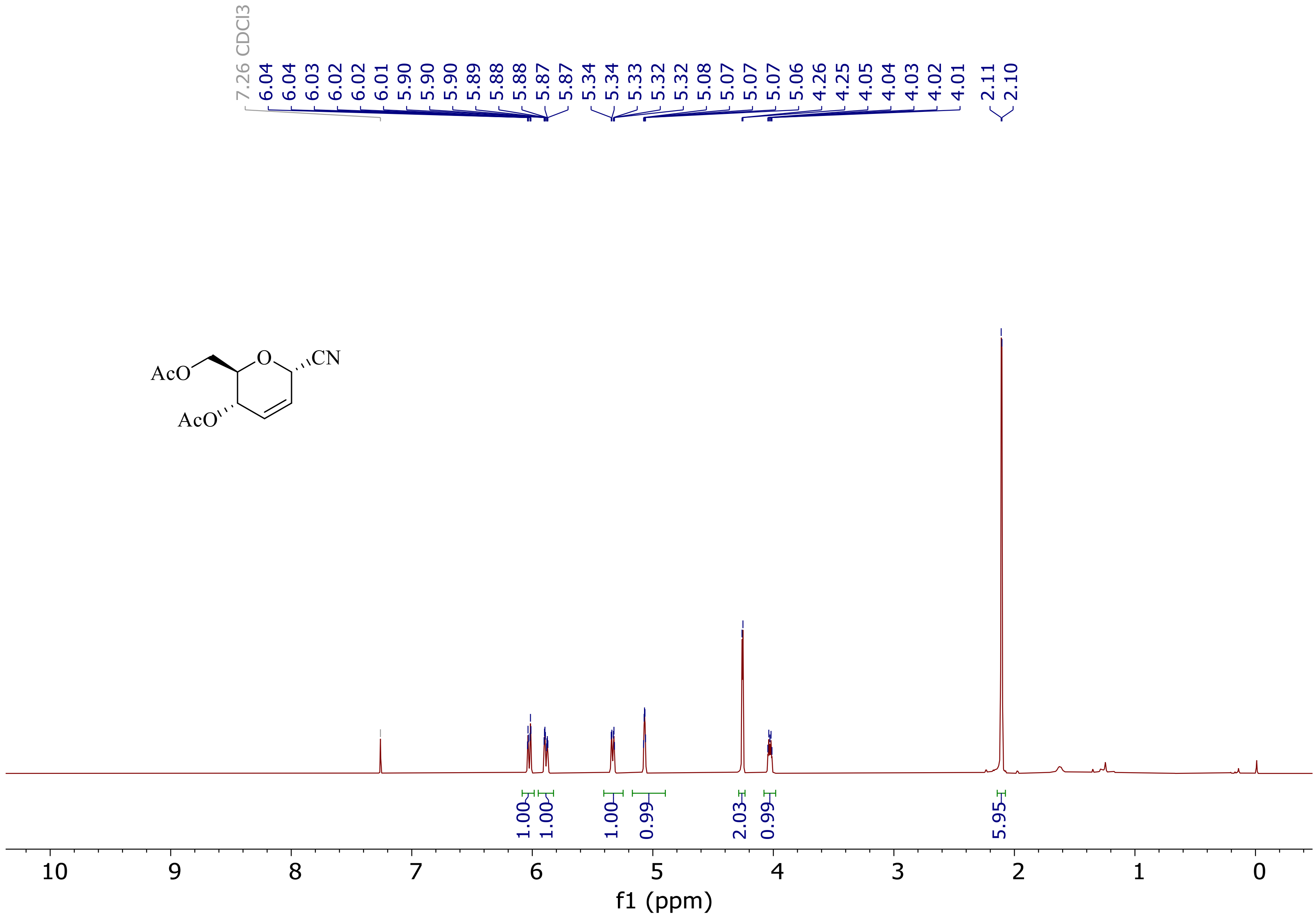

Figure S7. ^{1}H NMR spectrum of compound α-**7** (500 MHz, $CDCl_3$).

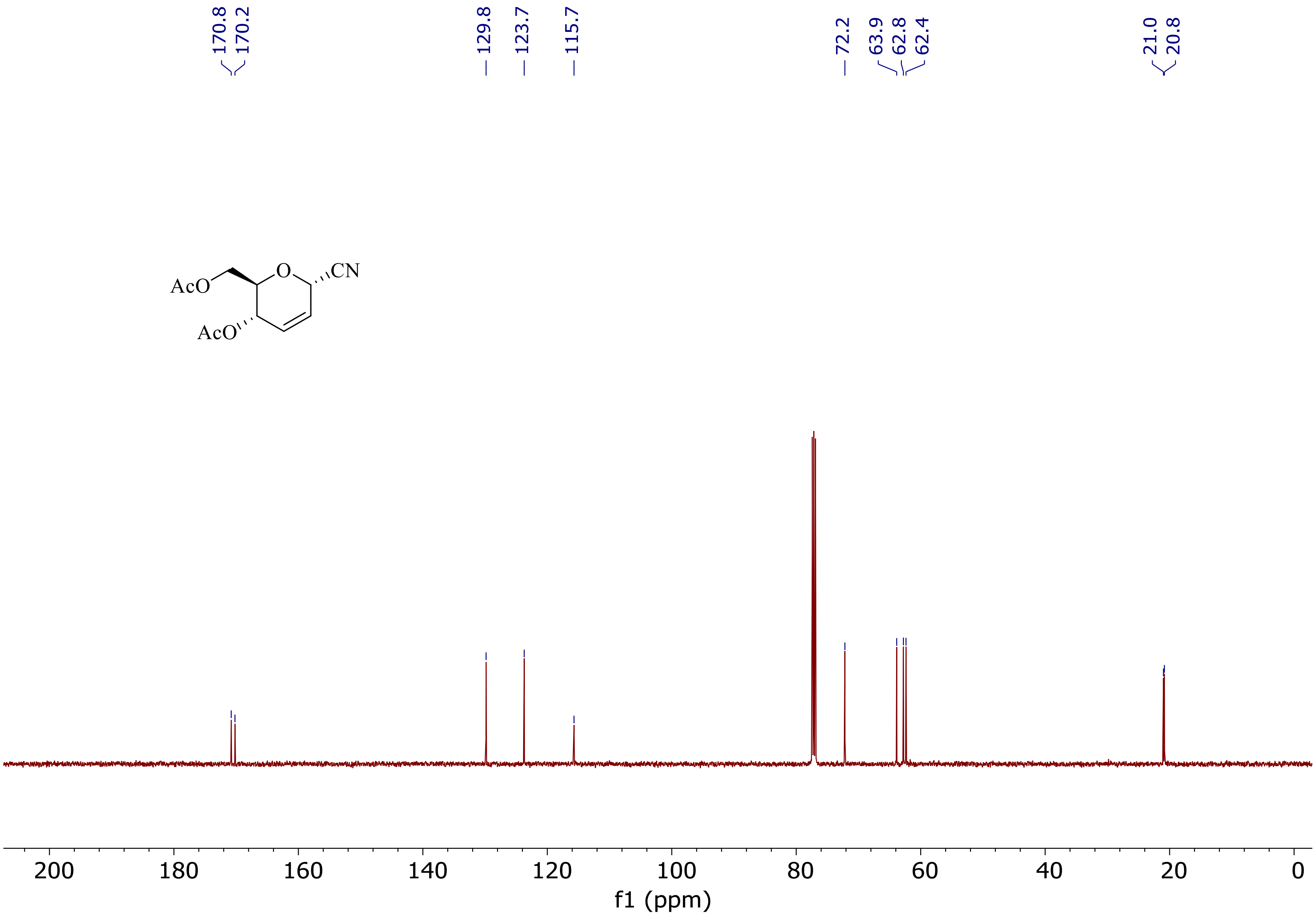

Figure S8. $^{13}C\{^{1}H\}$ NMR spectrum of compound α-**7** (125 MHz, $CDCl_3$).

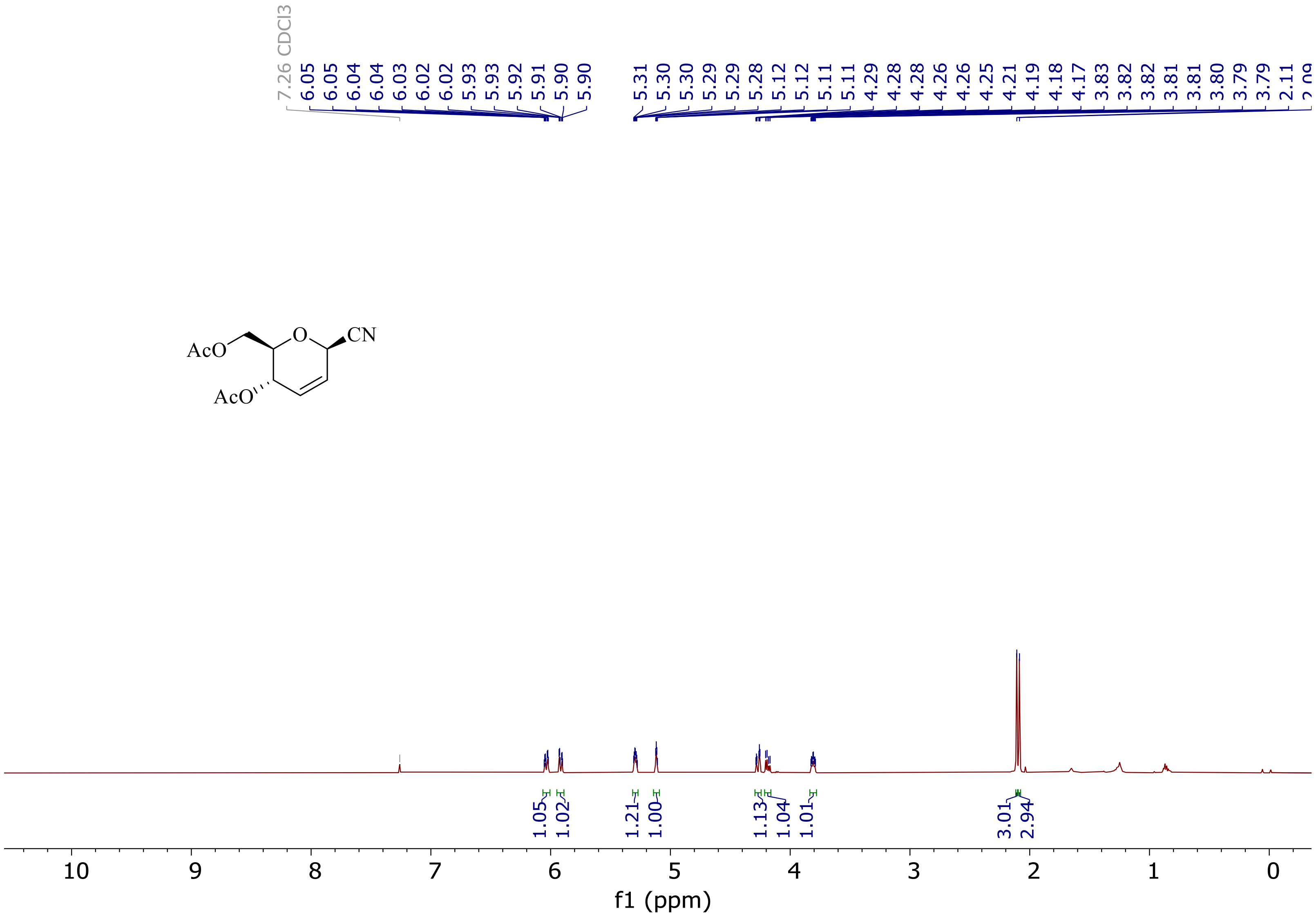

Figure S9. ¹H NMR spectrum of compound β-**7** (500 MHz, $CDCl_3$).

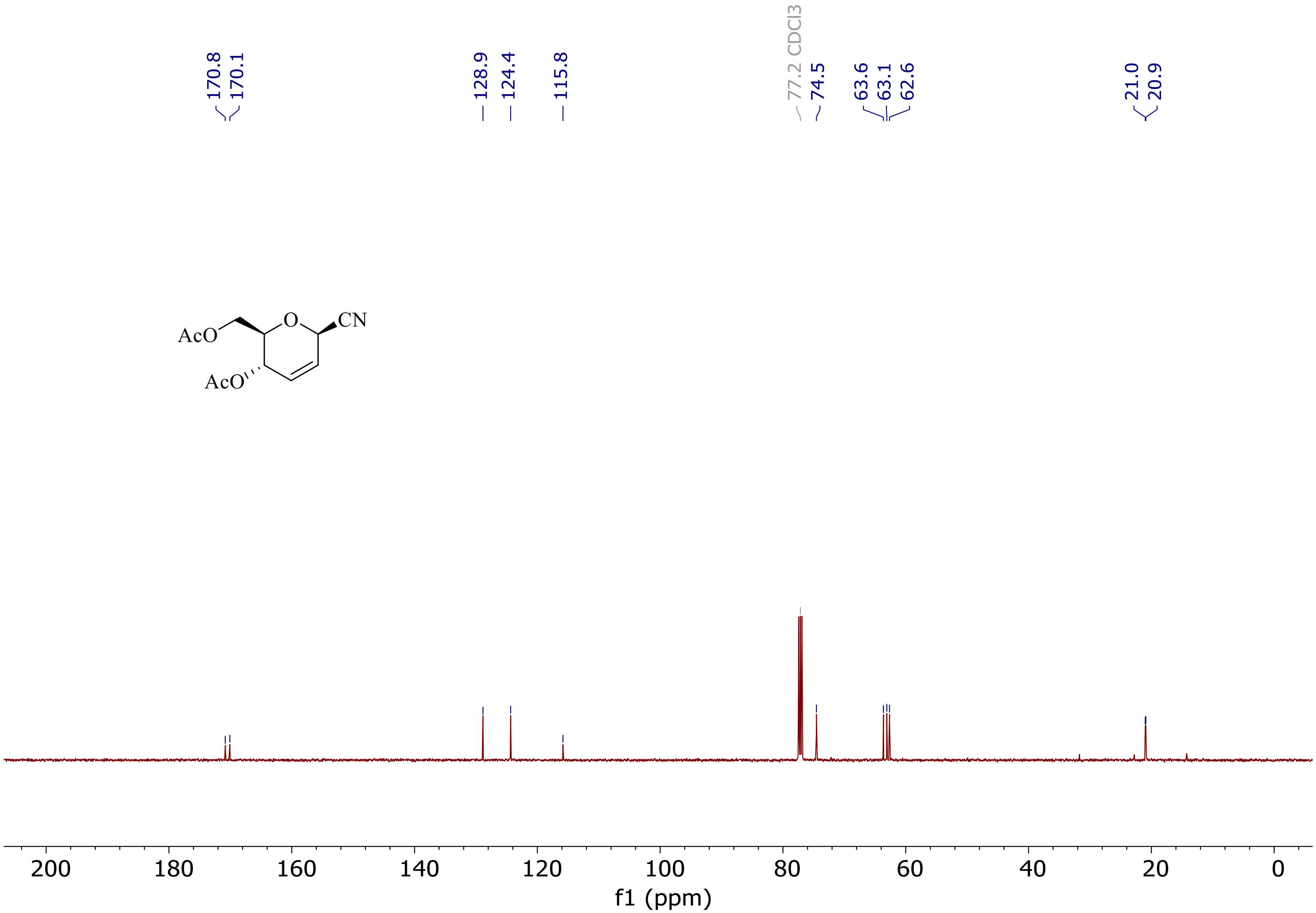

Figure S10. $^{13}C\{^{1}H\}$ NMR spectrum of compound β-**7** (125 MHz, $CDCl_3$).

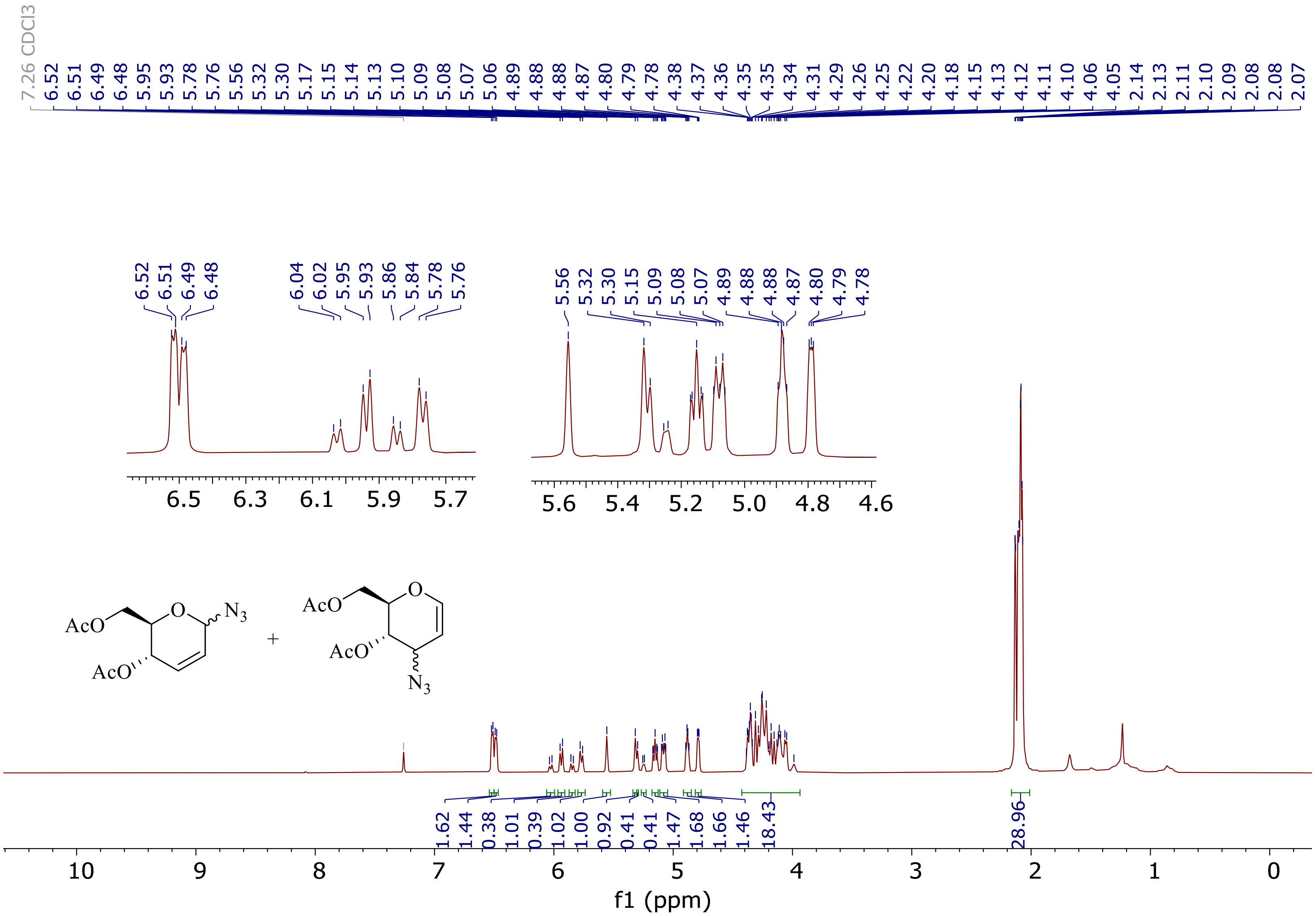

Figure S11. ^{1}H NMR spectrum of mixture **8a** + **8b** (500 MHz, $CDCl_3$).

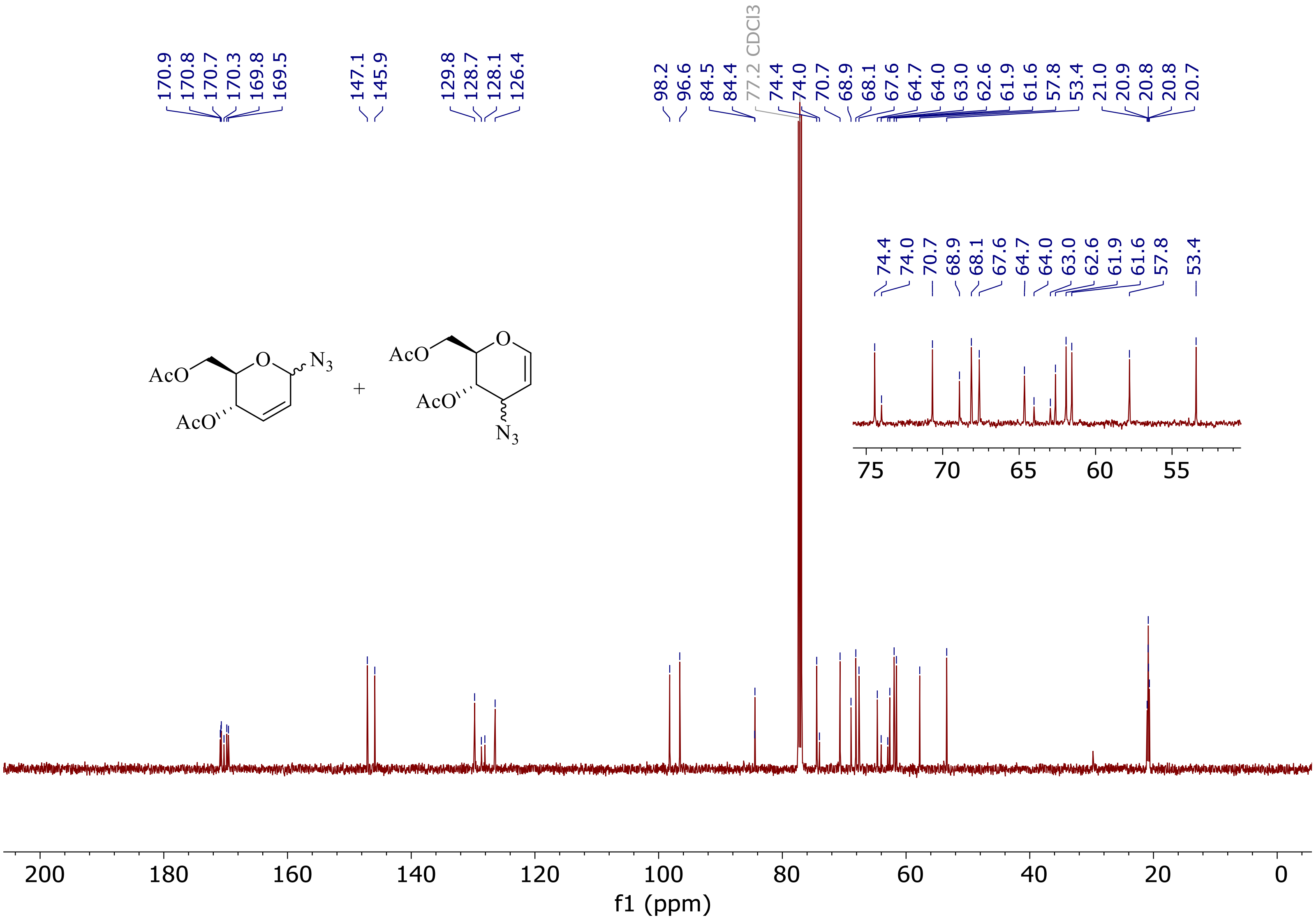

Figure S12. $^{13}C\{^{1}H\}$ NMR spectrum of mixture **8a** + **8b** (125 MHz, $CDCl_3$).

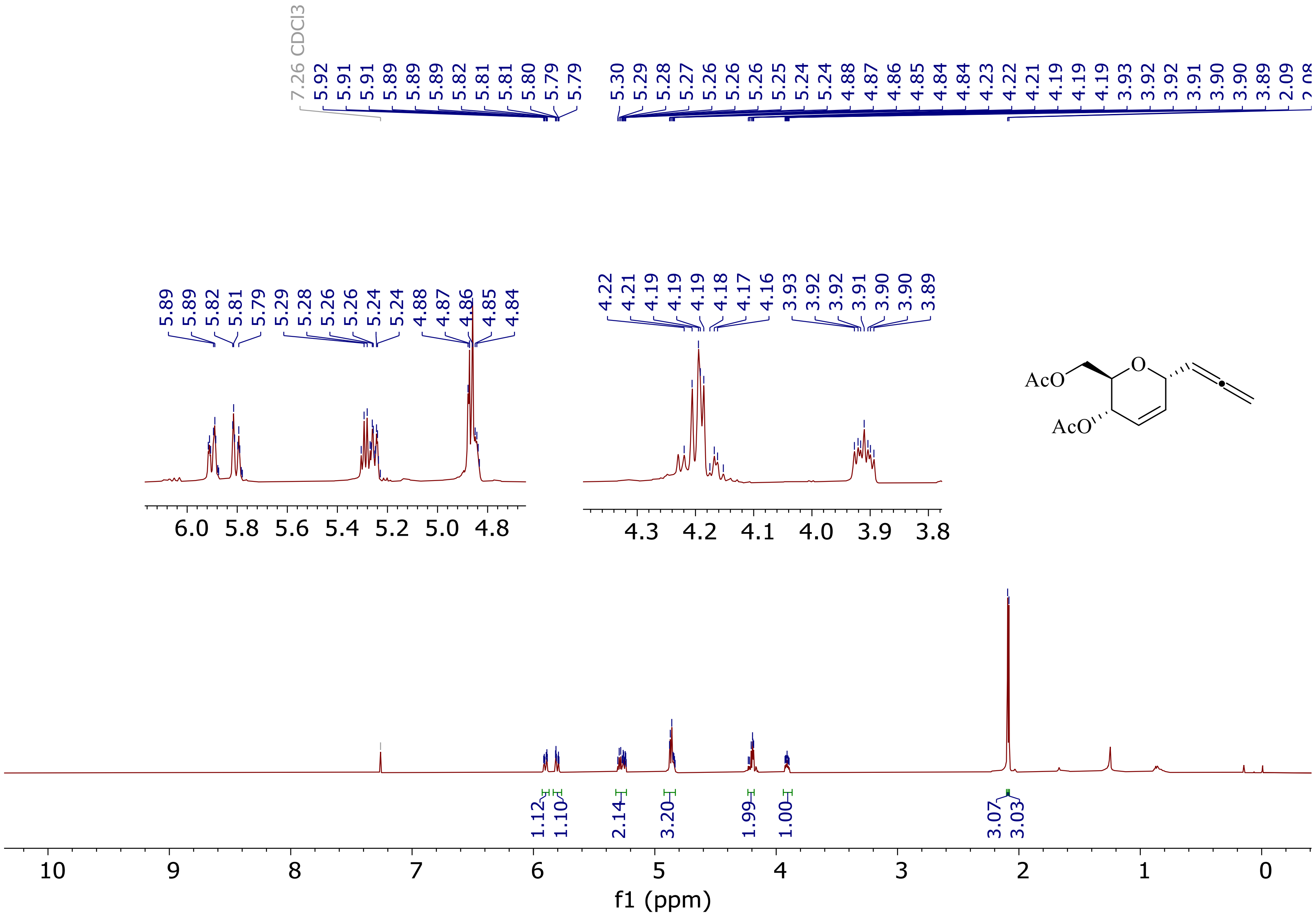

Figure S13. ^{1}H NMR spectrum of compound **9** (500 MHz, $CDCl_3$).

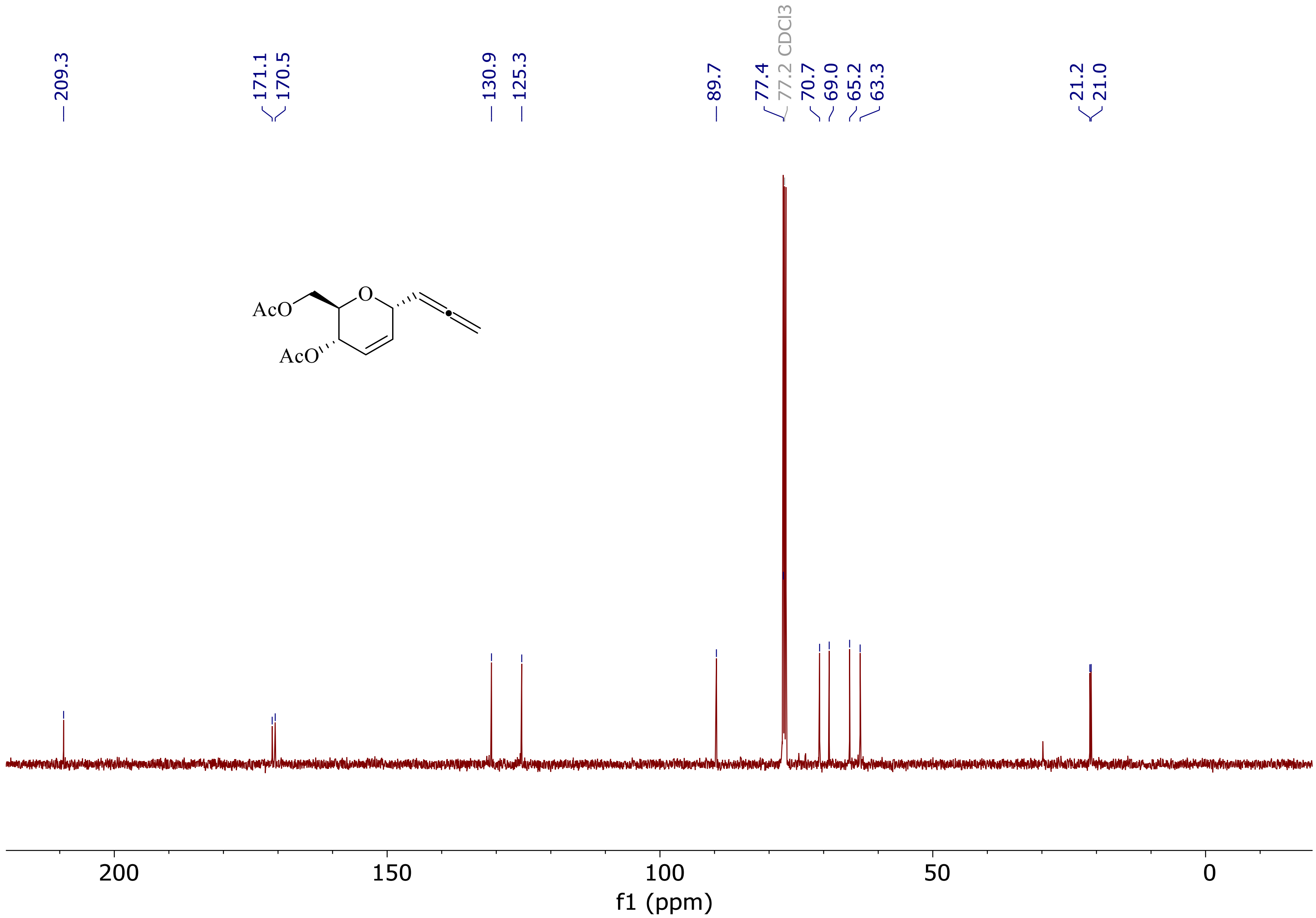

Figure S14. $^{13}C\{^{1}H\}$ NMR spectrum of compound **9** (125 MHz, $CDCl_3$).

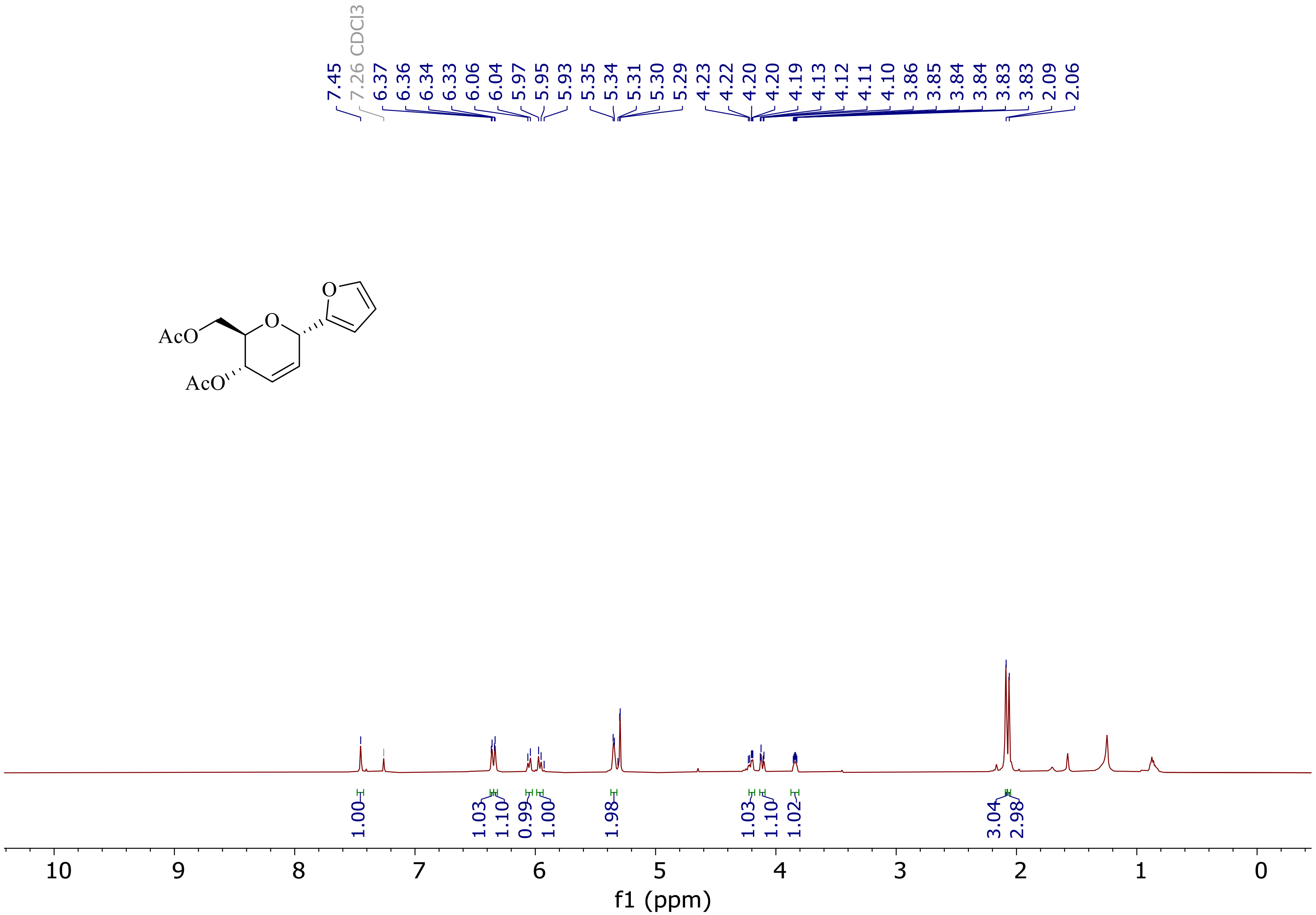

Figure S15. ^{1}H NMR spectrum of compound **10a** (500 MHz, $CDCl_3$).

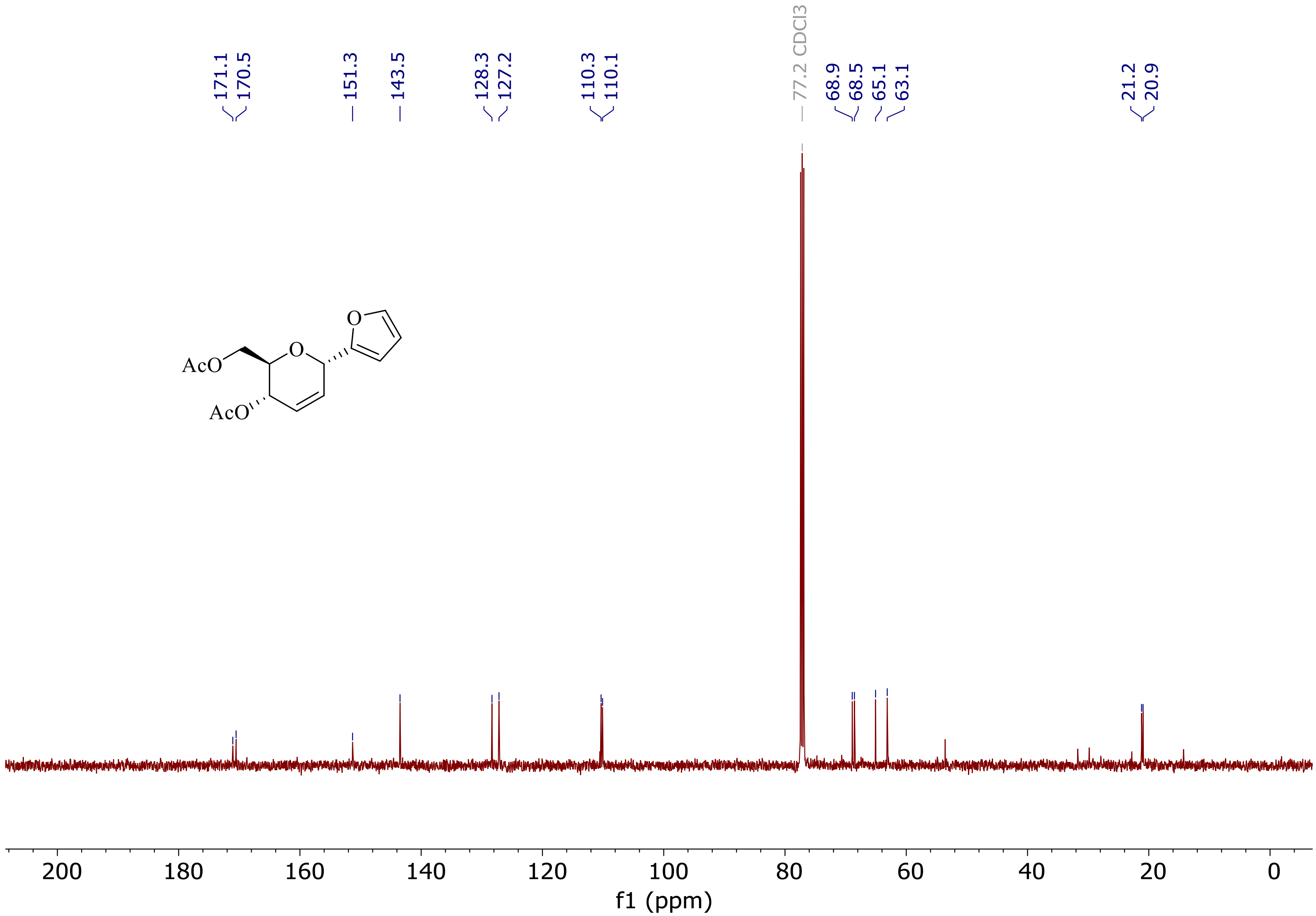

Figure S16. $^{13}C\{^{1}H\}$ NMR spectrum of compound **10a** (125 MHz, $CDCl_3$).

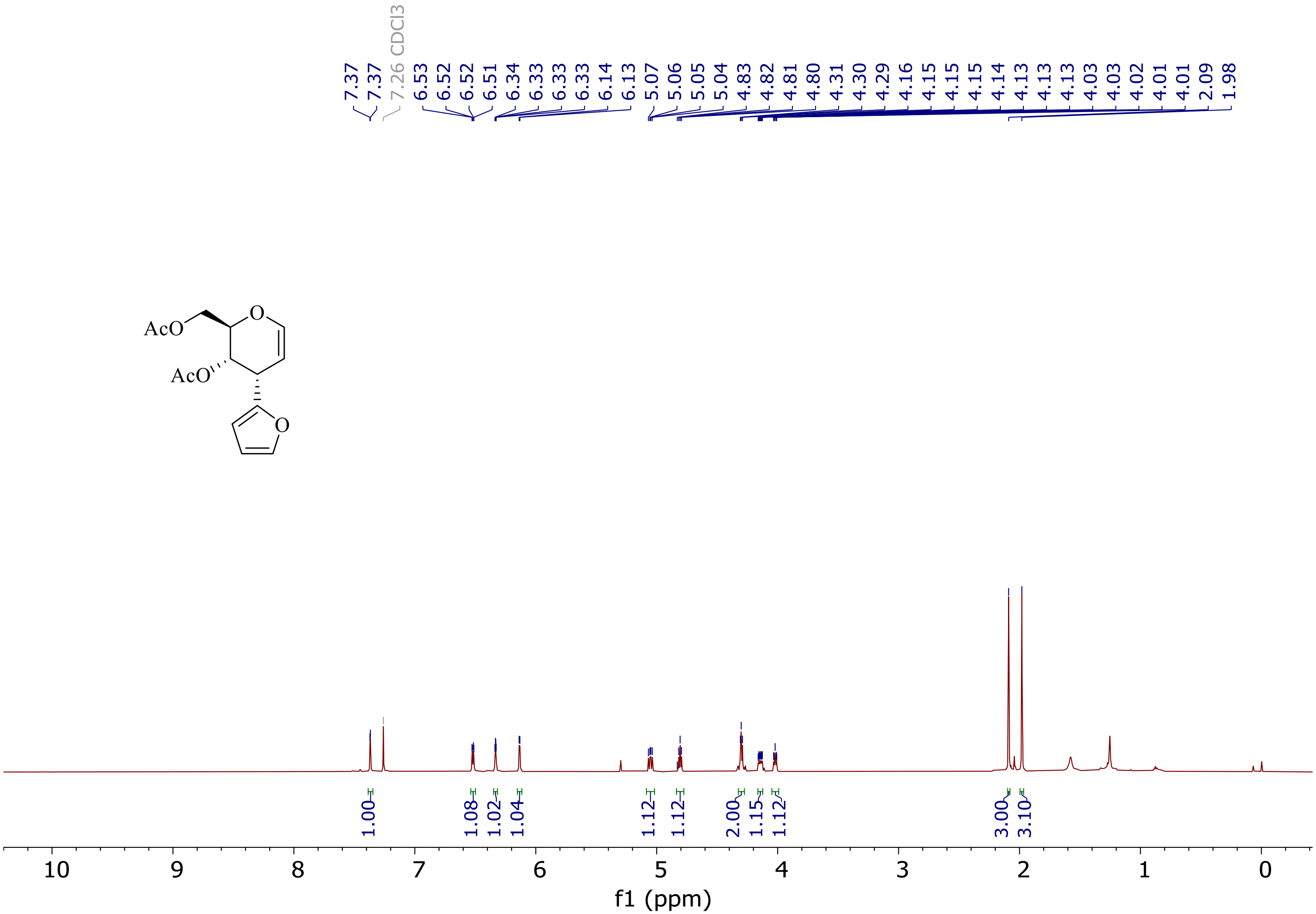

Figure S17. ^{1}H NMR spectrum of compound **10b** (500 MHz, $CDCl_3$).

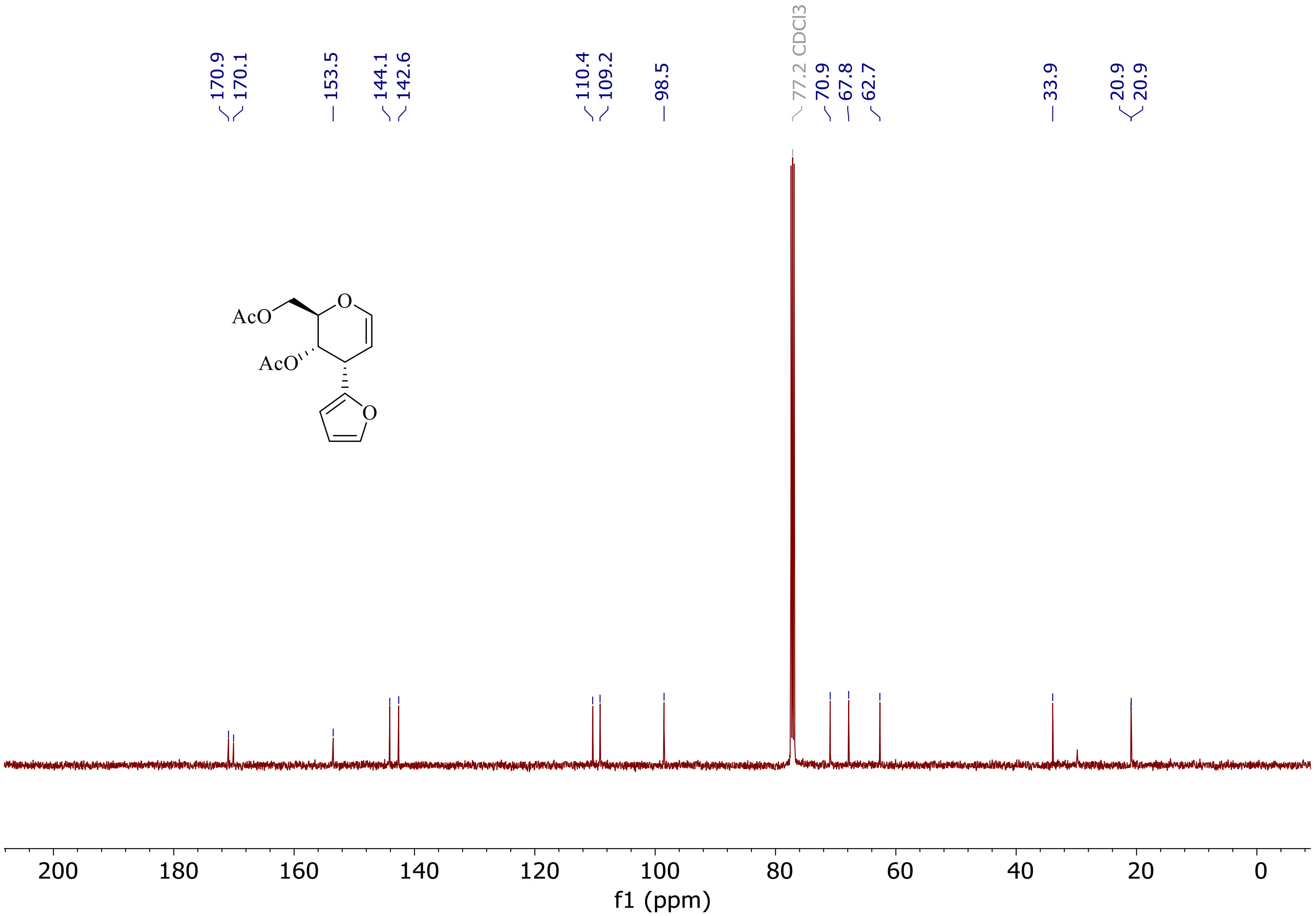

Figure S18. $^{13}C\{^{1}H\}$ NMR spectrum of compound **10b** (125 MHz, $CDCl_3$).

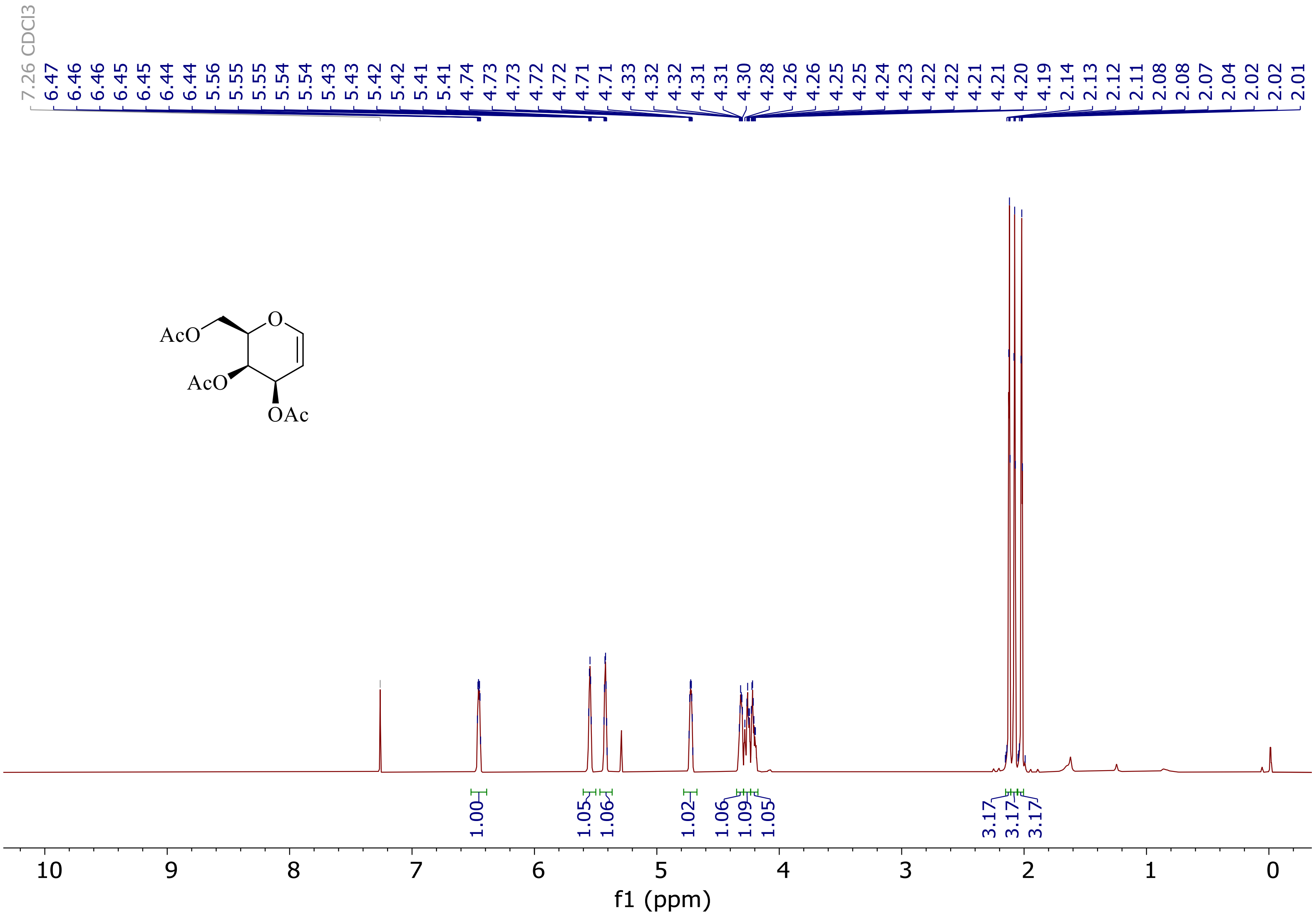

Figure S19. ^{1}H NMR spectrum of compound **11** (500 MHz, $CDCl_3$).

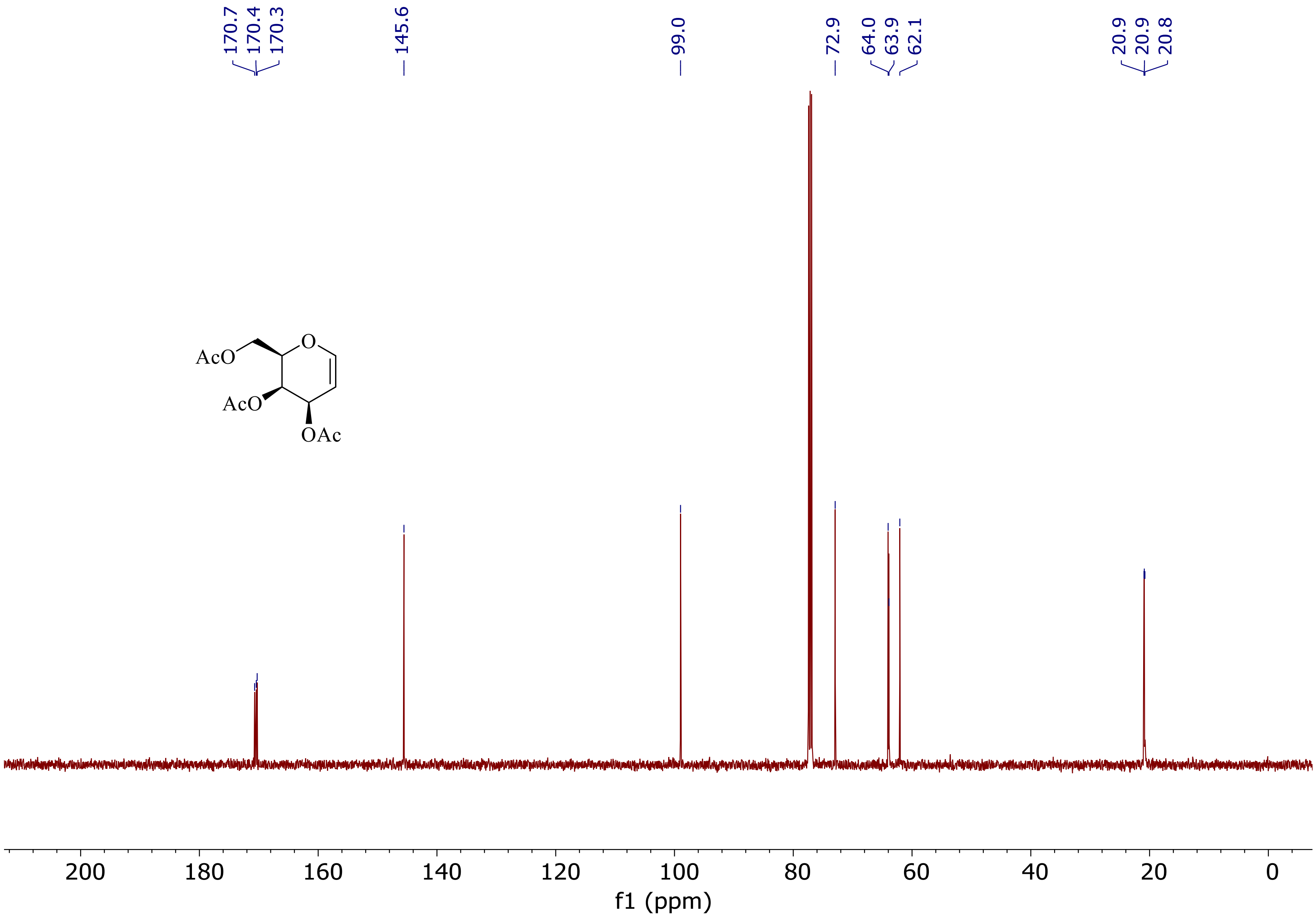

Figure S20. $^{13}C\{^{1}H\}$ NMR spectrum of compound **11** (125 MHz, $CDCl_3$).

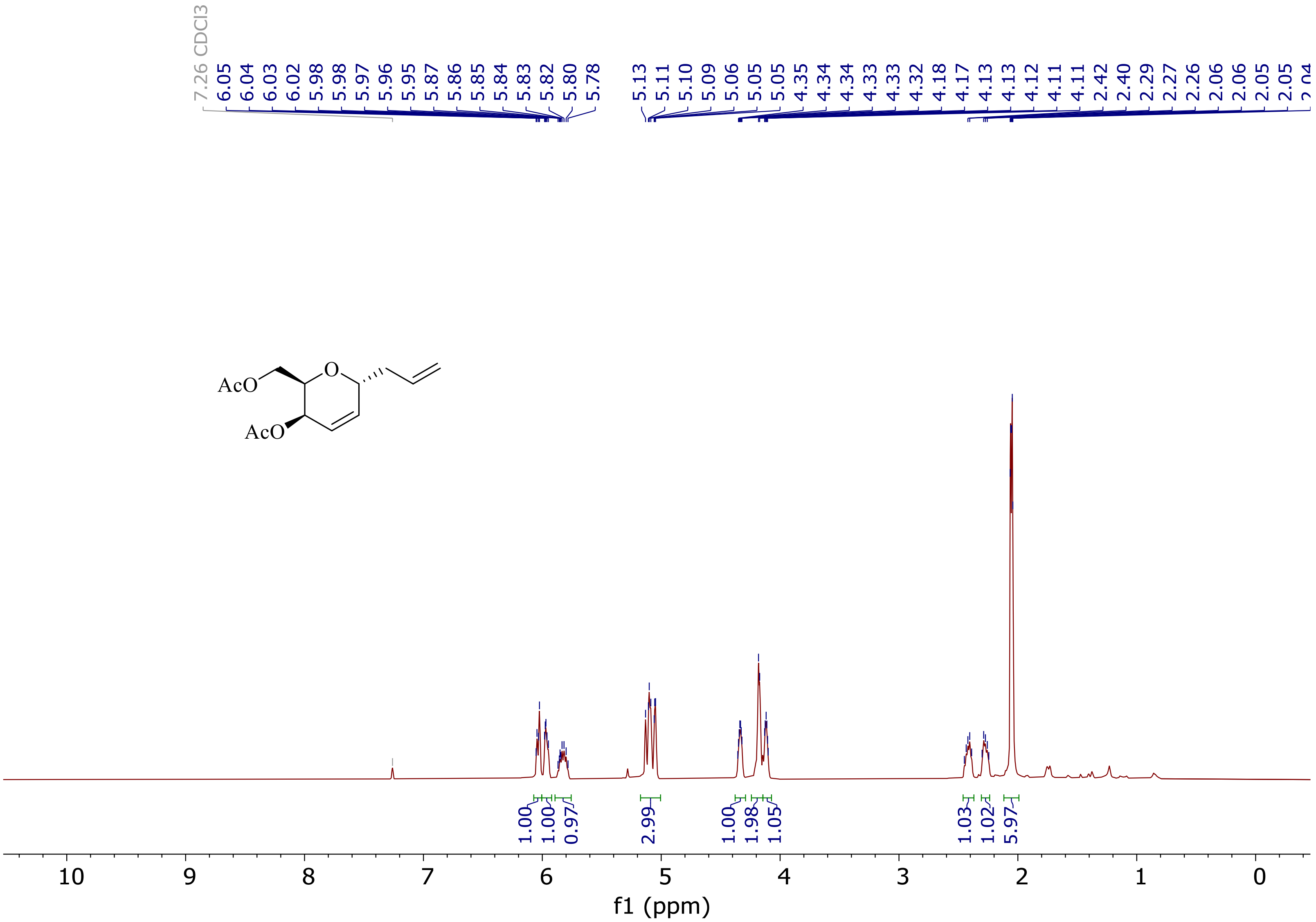

Figure S21. ^{1}H NMR spectrum of compound **12** (500 MHz, $CDCl_3$).

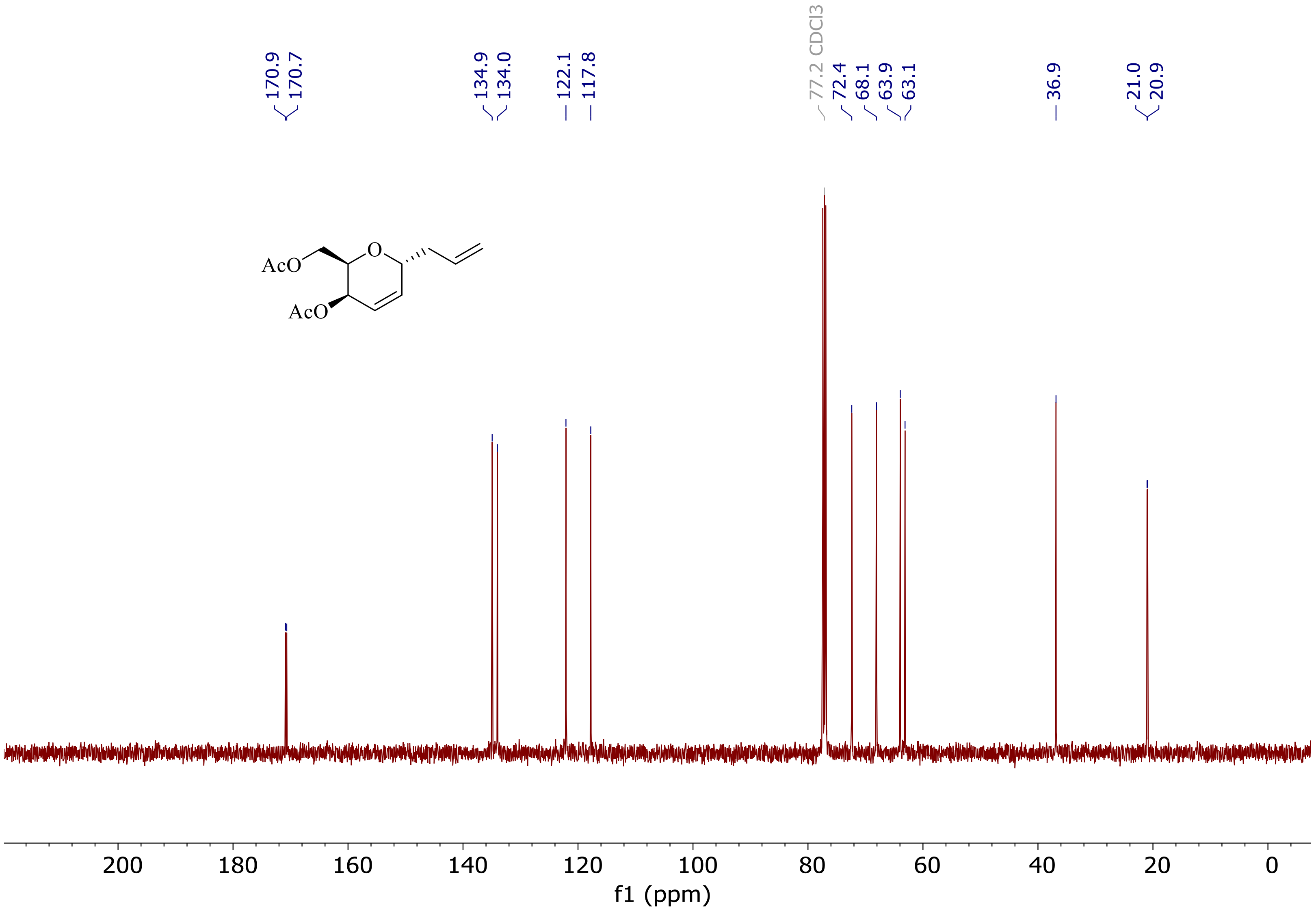

Figure S22. $^{13}C\{^{1}H\}$ NMR spectrum of compound **12** (125 MHz, $CDCl_3$).

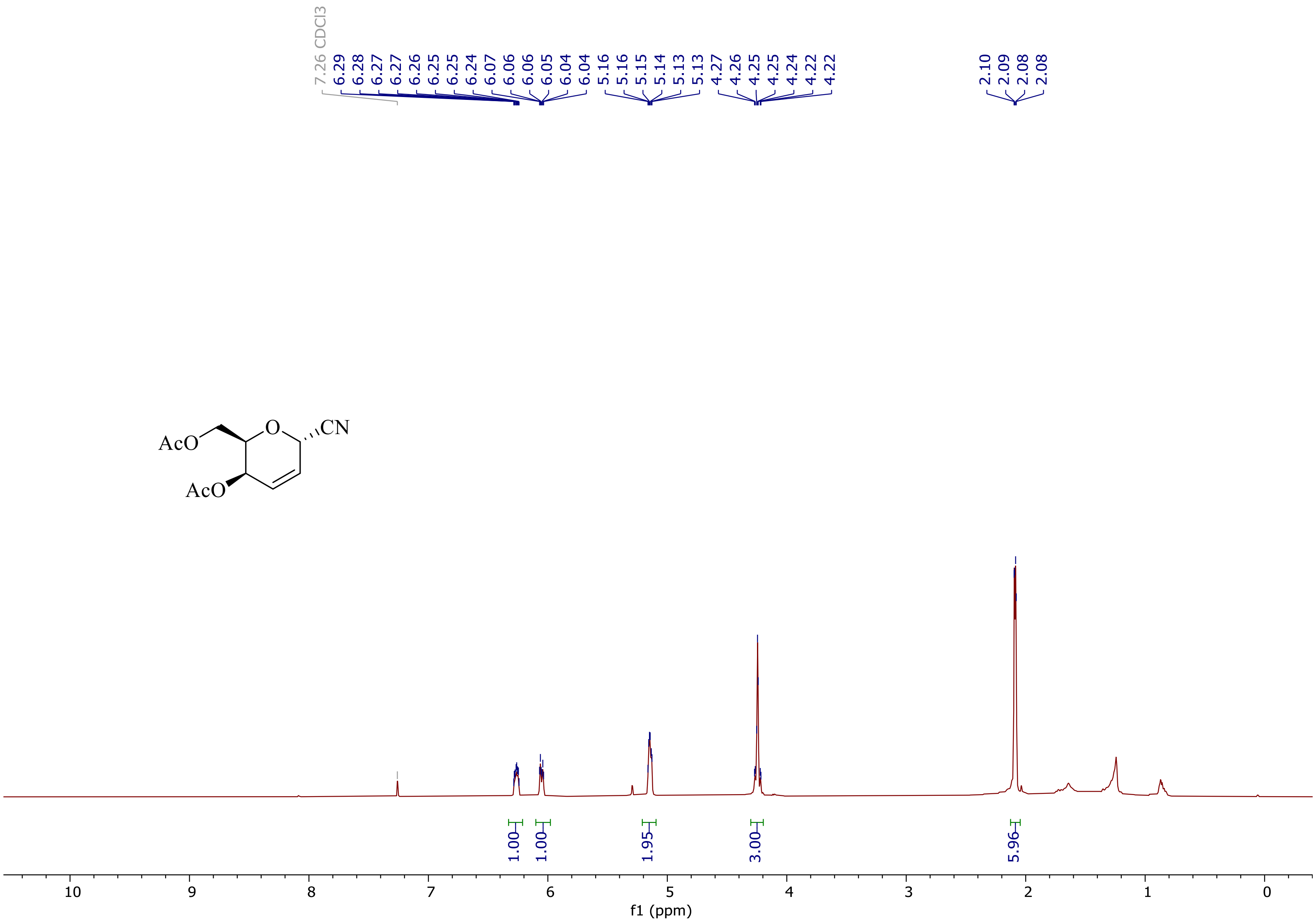

Figure S23. ^{1}H NMR spectrum of compound α-**13** (500 MHz, $CDCl_3$).

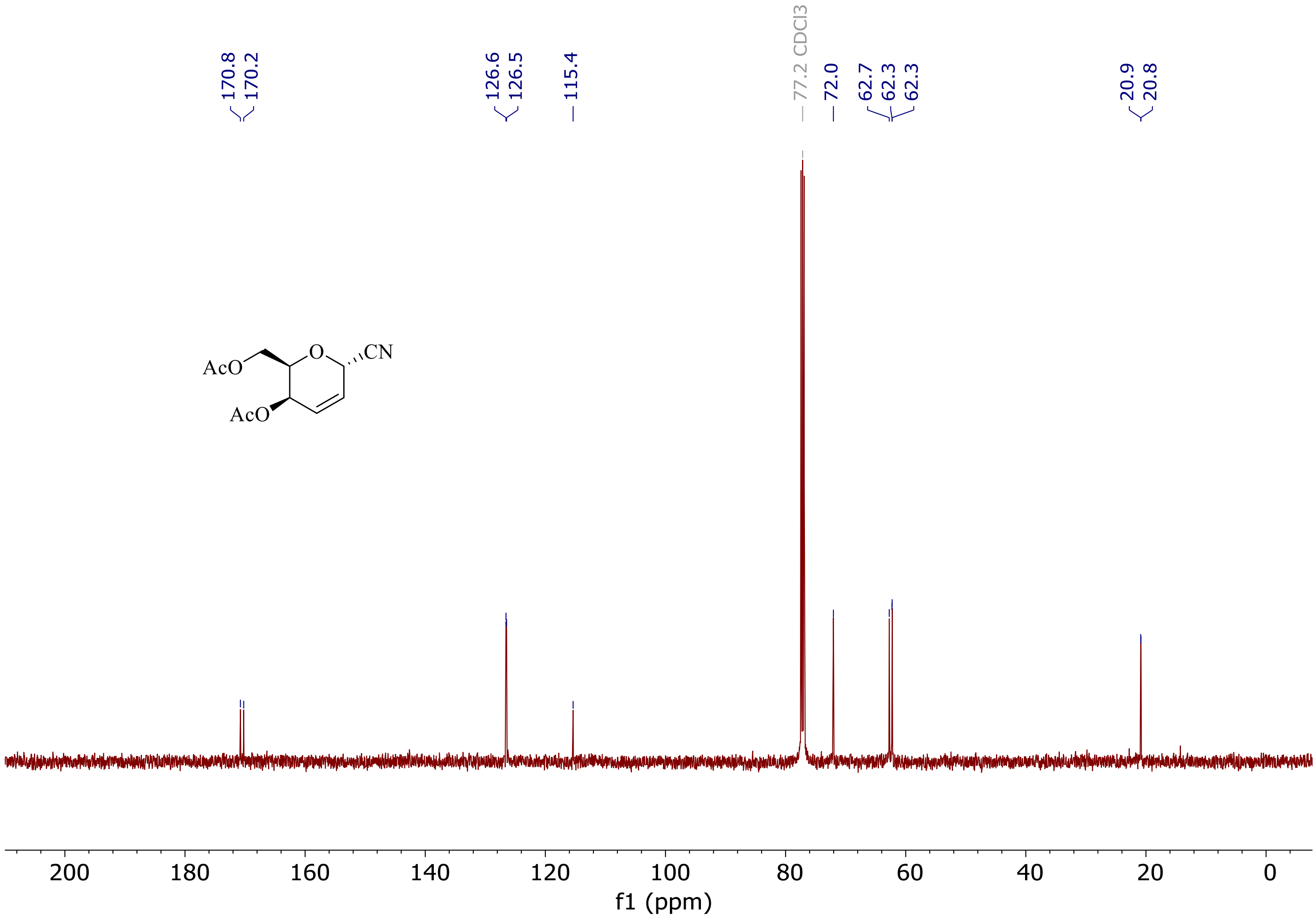

Figure S24. $^{13}C\{^{1}H\}$ NMR spectrum of compound α-**13** (125 MHz, $CDCl_3$).

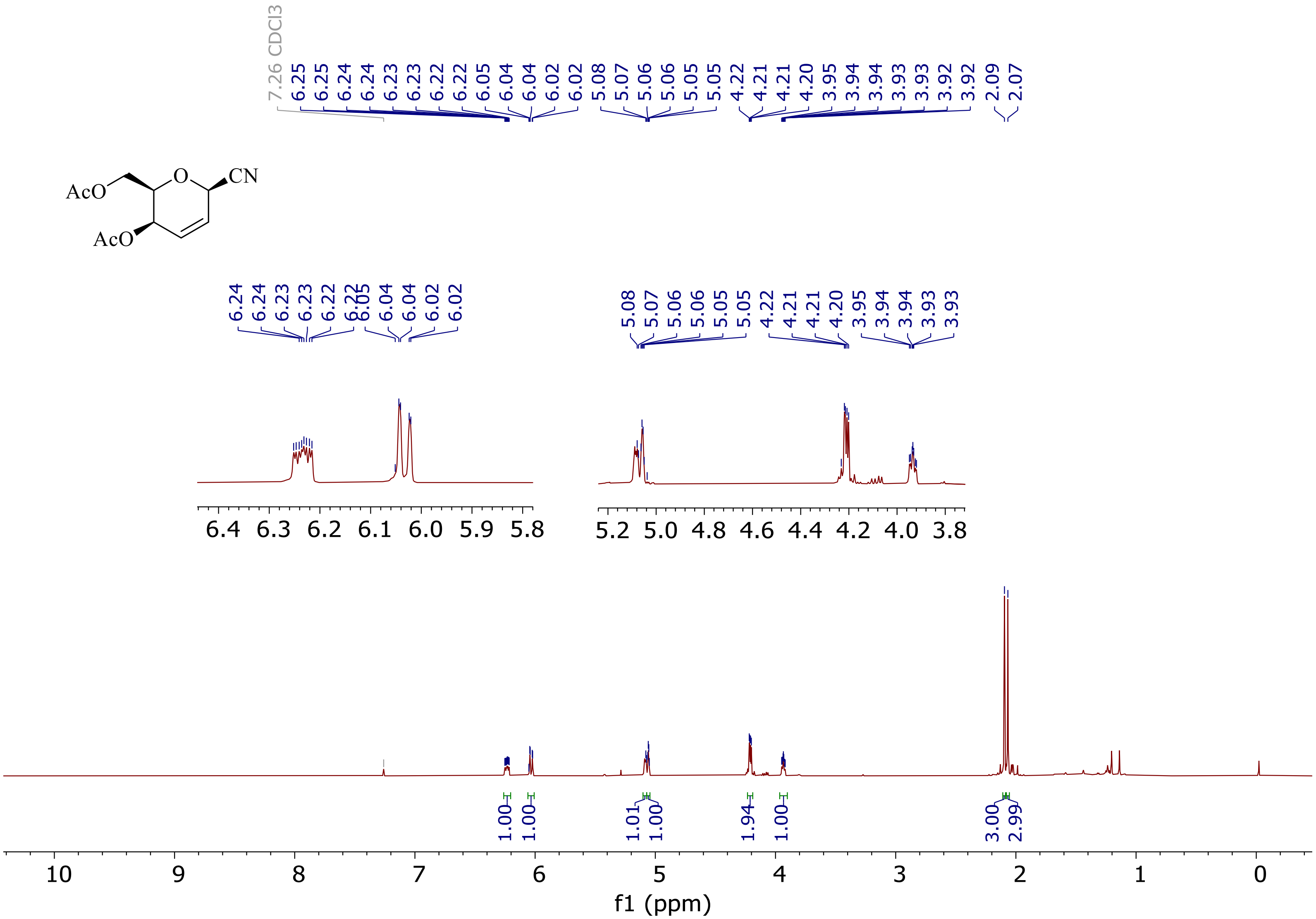

Figure S25. ^{1}H NMR spectrum of compound β-**13** (500 MHz, CDCl3).

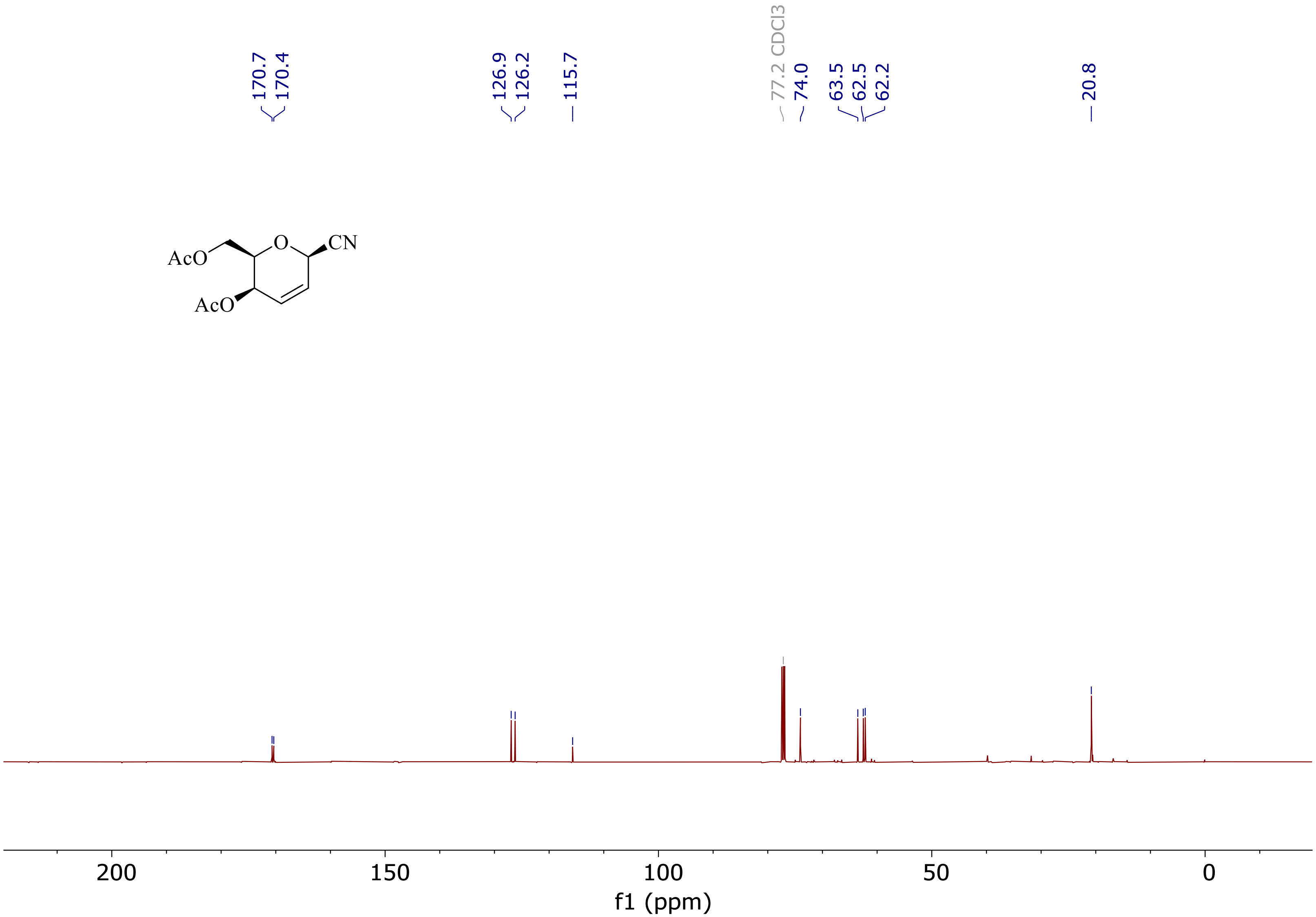

Figure S26. $^{13}C\{^{1}H\}$ NMR spectrum of compound β-**13** (125 MHz, $CDCl_3$).

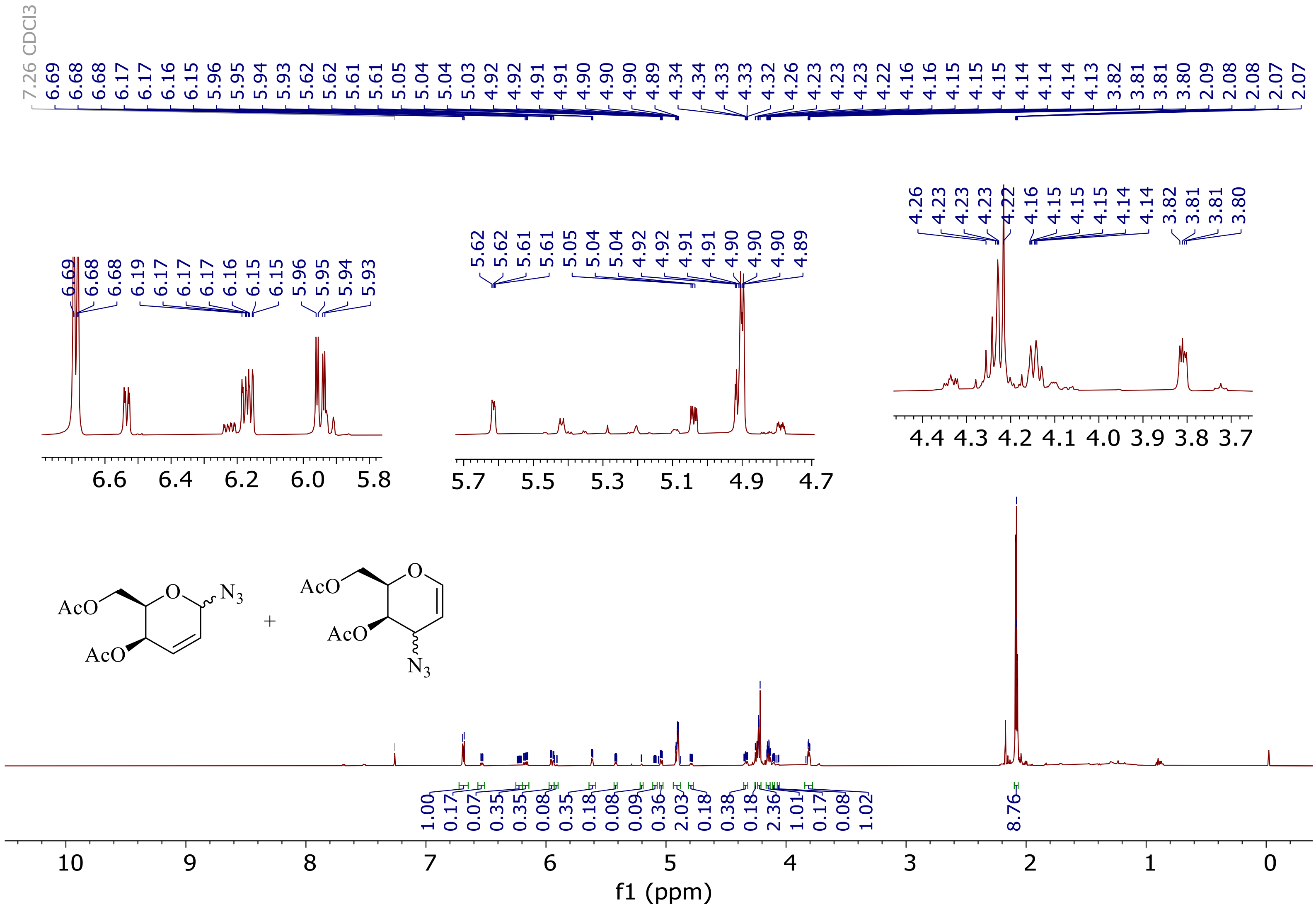

Figure S27. ^{1}H NMR spectrum of mixture **14a** + **14b** (500 MHz, $CDCl_3$).

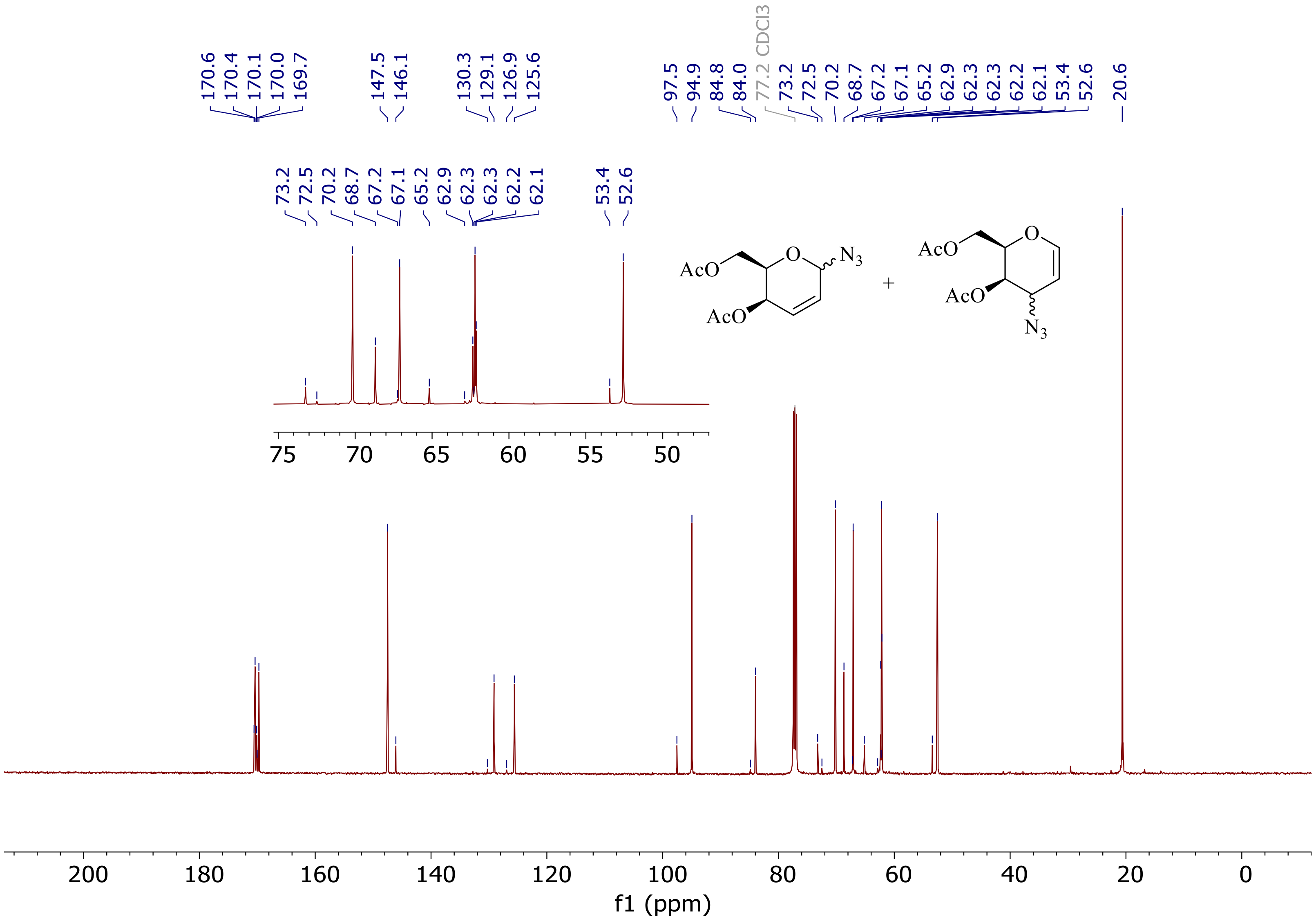

Figure S28. $^{13}C\{^{1}H\}$ NMR spectrum of mixture **14a** + **14b** (125 MHz, $CDCl_3$).

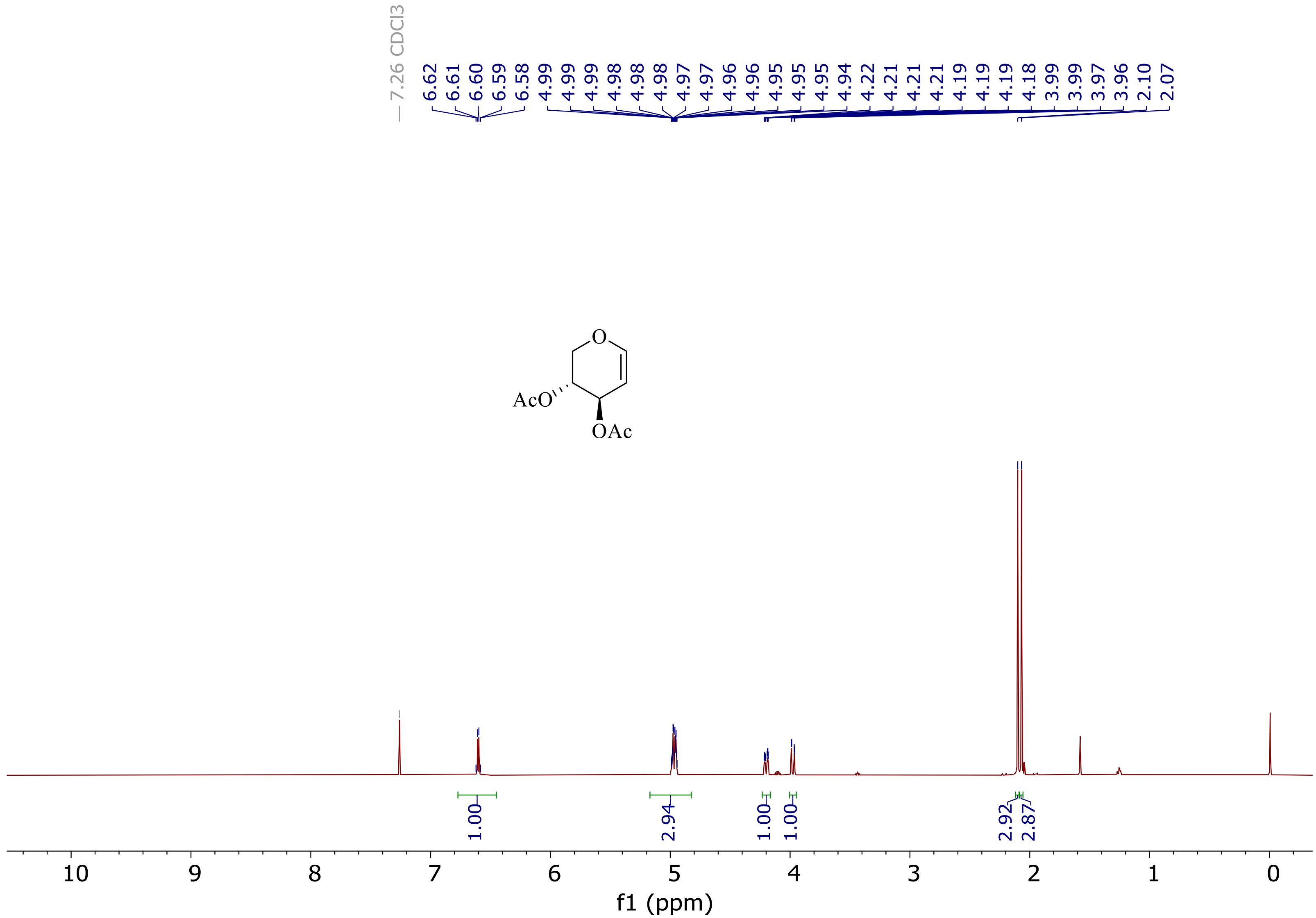

Figure S29. ^{1}H NMR spectrum of **15** (500 MHz, $CDCl_3$).

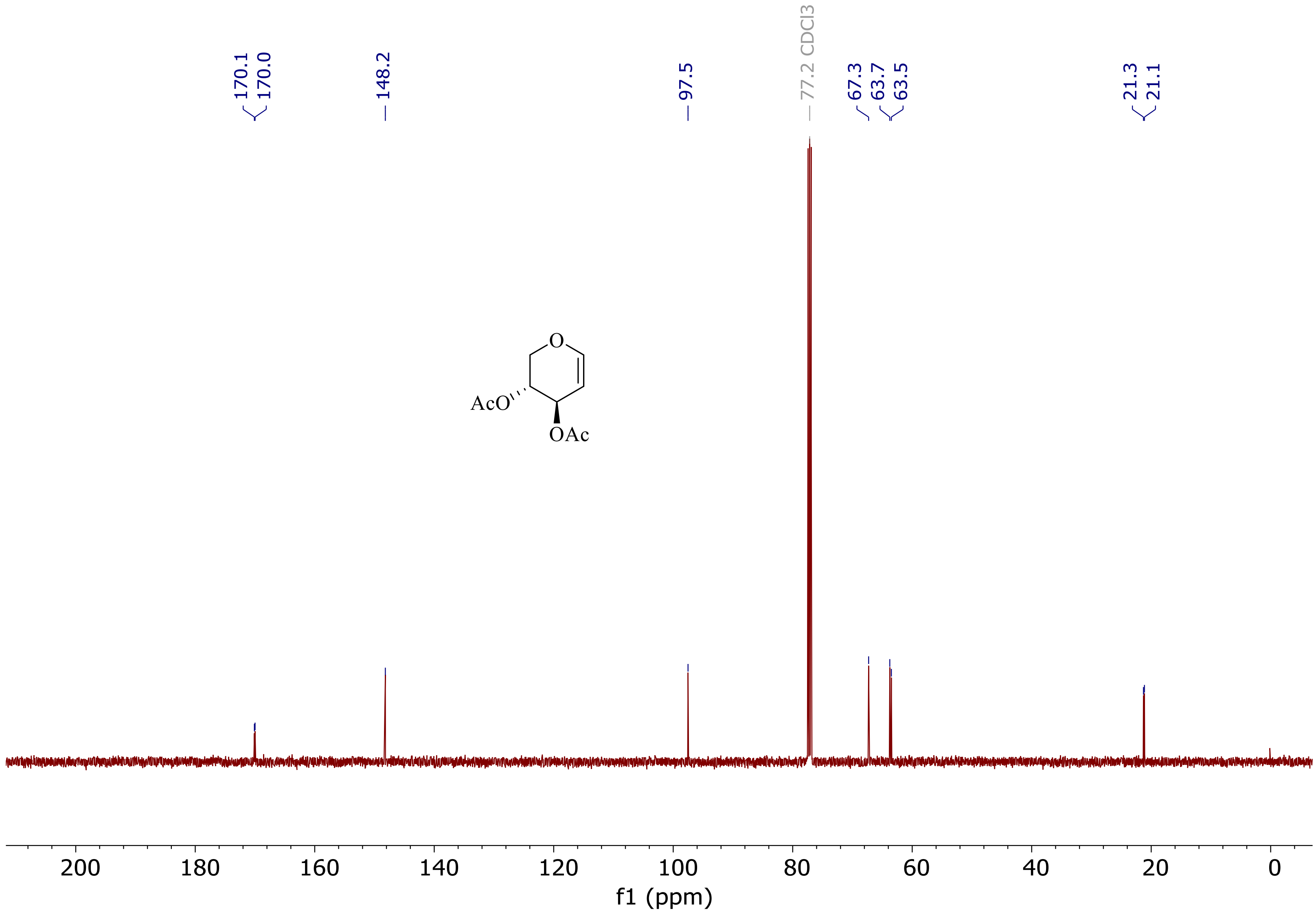

Figure S30. $^{13}C\{^1H\}$ NMR spectrum of **15** (125 MHz, $CDCl_3$).

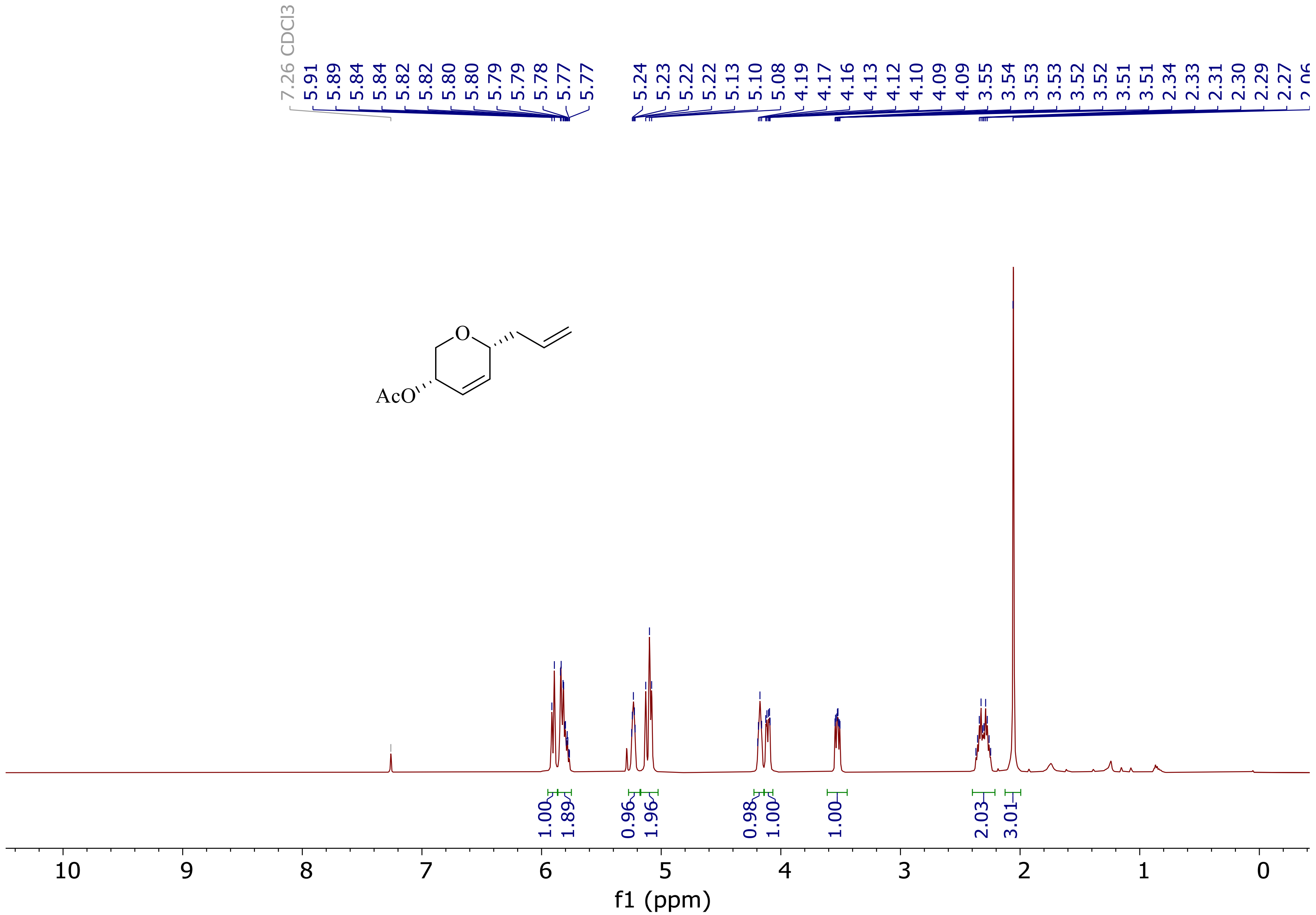

Figure S31. ^{1}H NMR spectrum of α-**16** (500 MHz, $CDCl_3$).

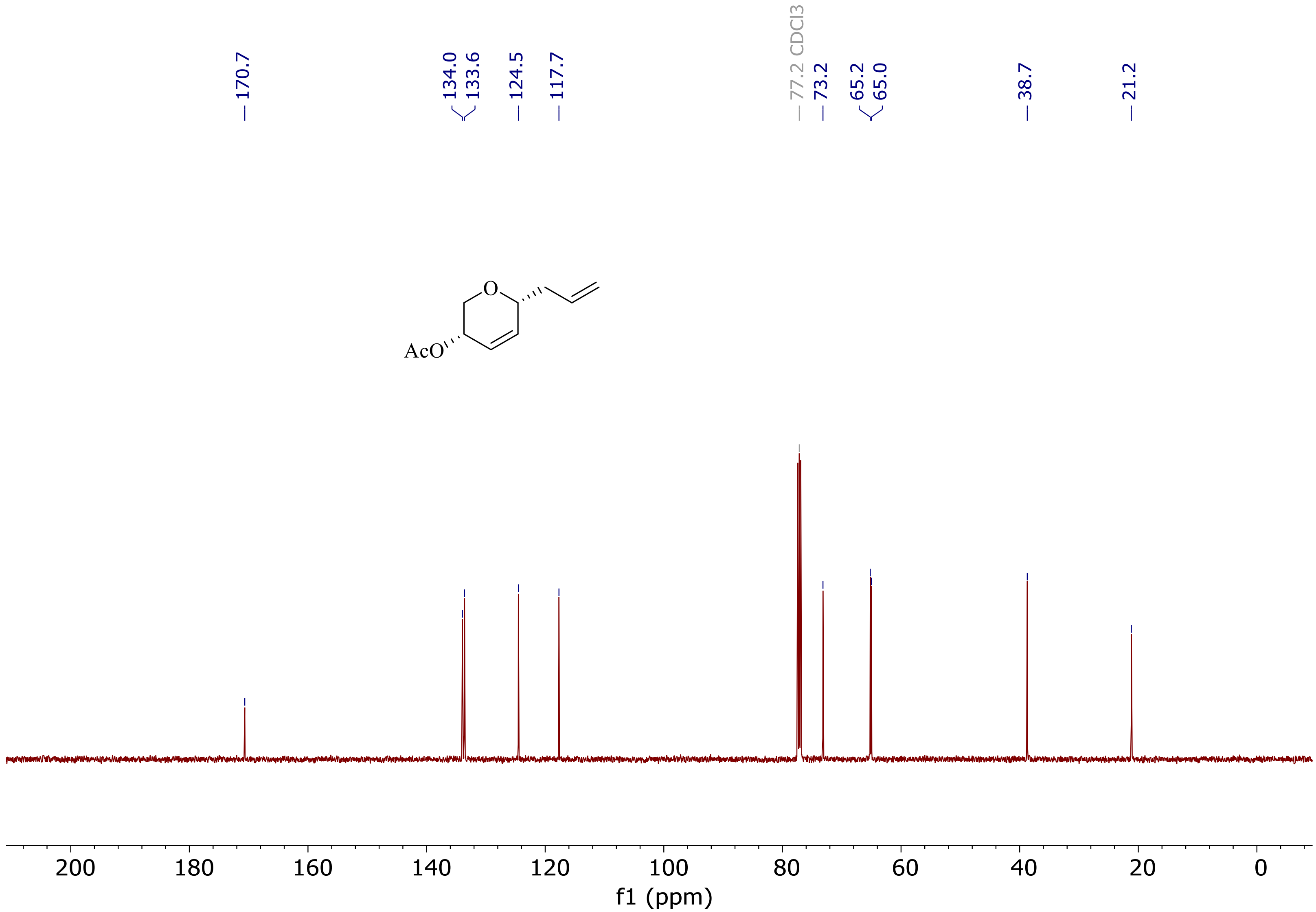

Figure S32. $^{13}C\{^{1}H\}$ NMR spectrum of α-**16** (125 MHz, $CDCl_3$).

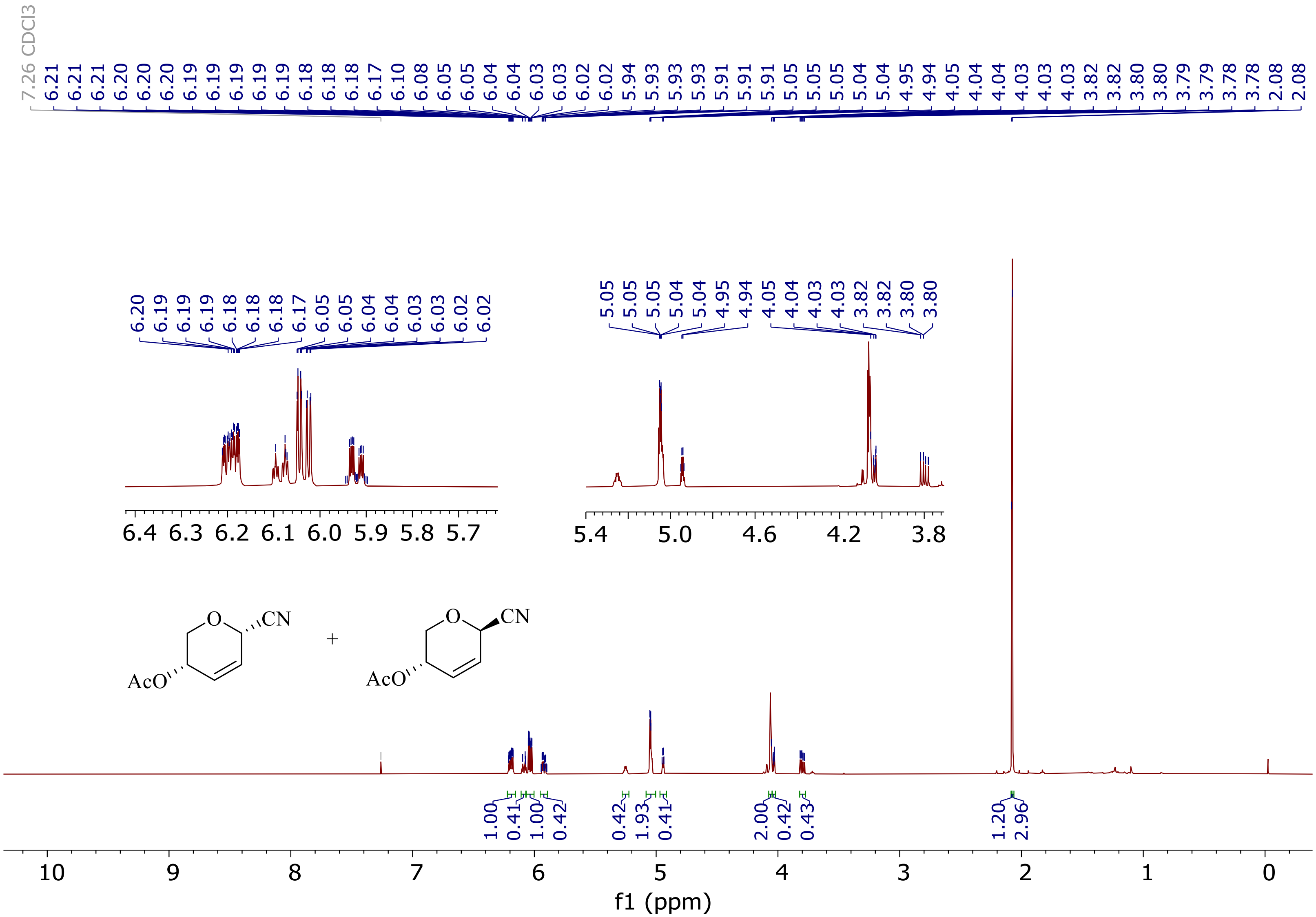

Figure S33. ^{1}H NMR spectrum of mixture α-**17** + β-**17** (500 MHz, $CDCl_3$).

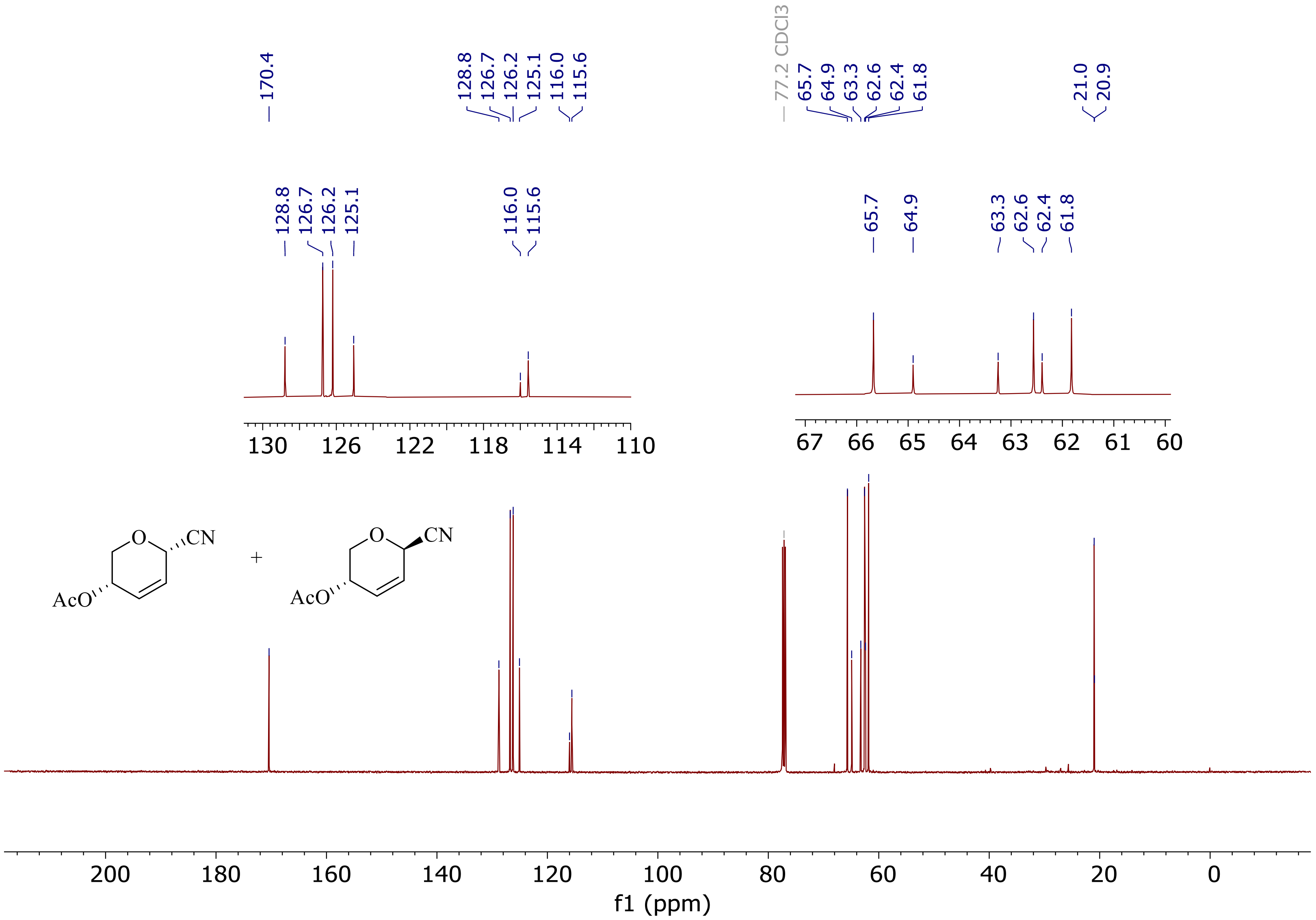

Figure S34. $^{13}C\{^{1}H\}$ NMR spectrum of mixture α-**17** + β-**17** (125 MHz, $CDCl_3$).

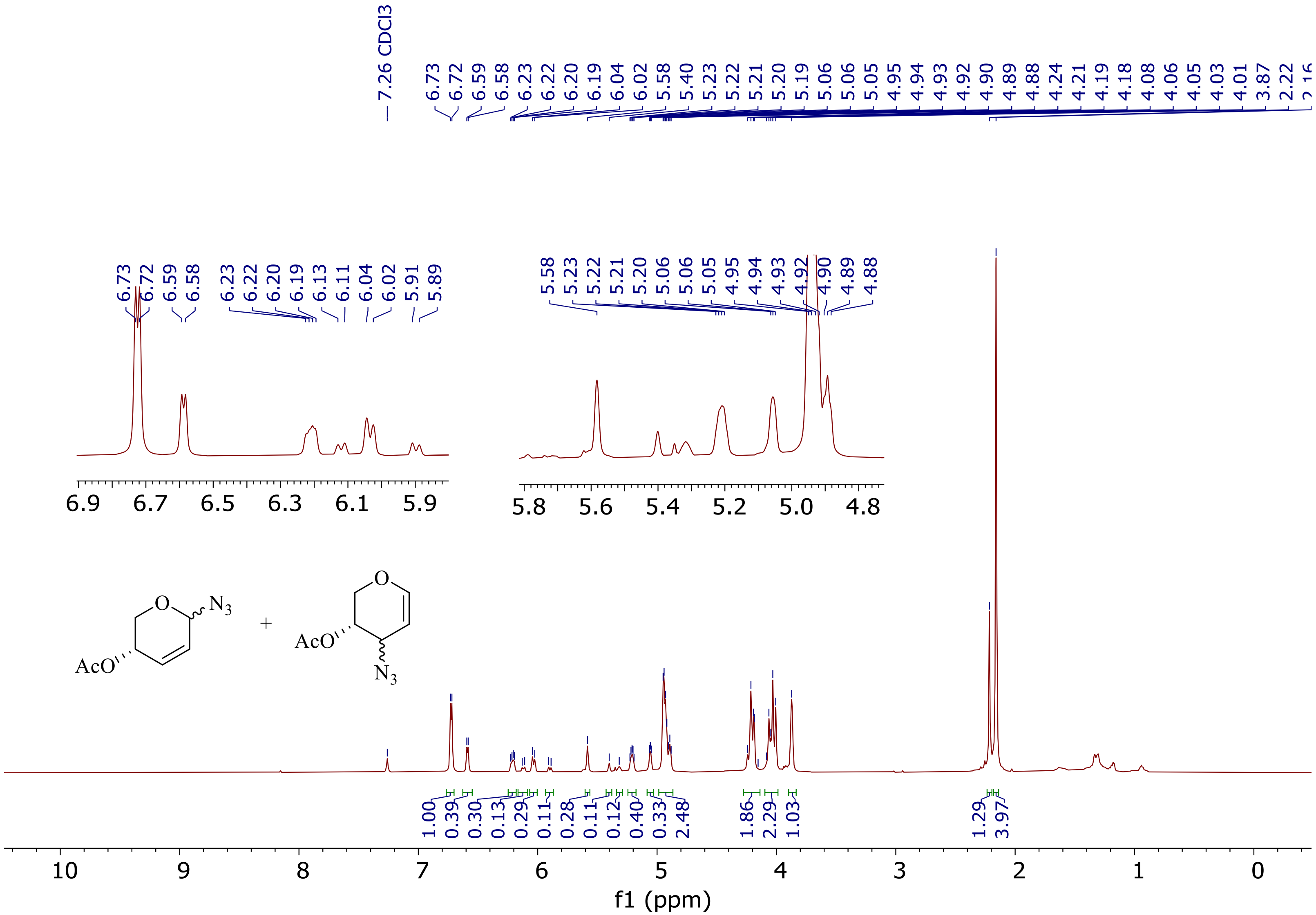

Figure S35. ^{1}H NMR spectrum of mixture **18a** + **18b** (500 MHz, $CDCl_3$).

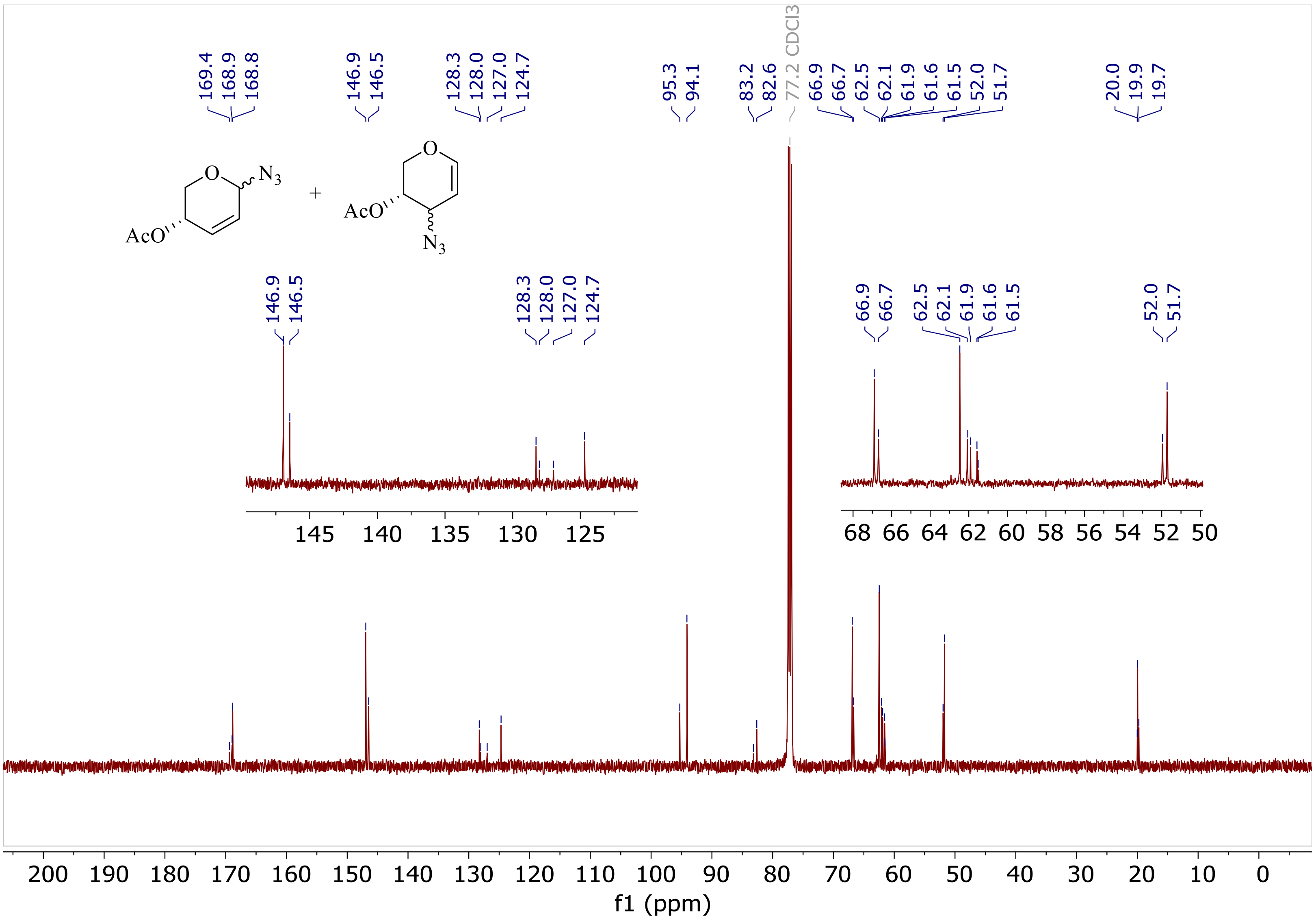

Figure S36. $^{13}C\{^{1}H\}$ NMR spectrum of 1**8a** + 1**8b** (125 MHz, $CDCl_3$).

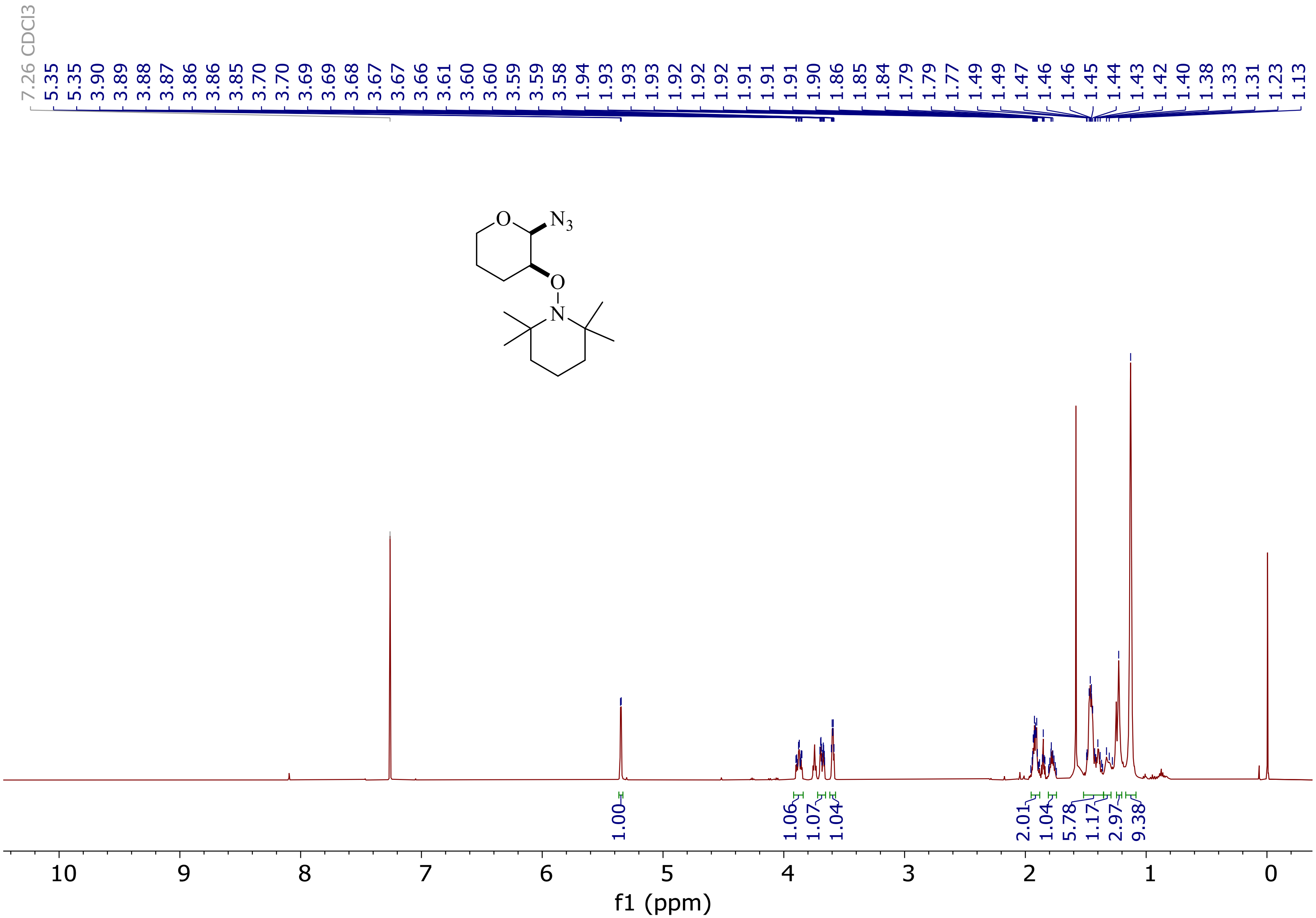

Figure S37. ^{1}H NMR spectrum of compound *cis*-**21** (500 MHz, $CDCl_3$).

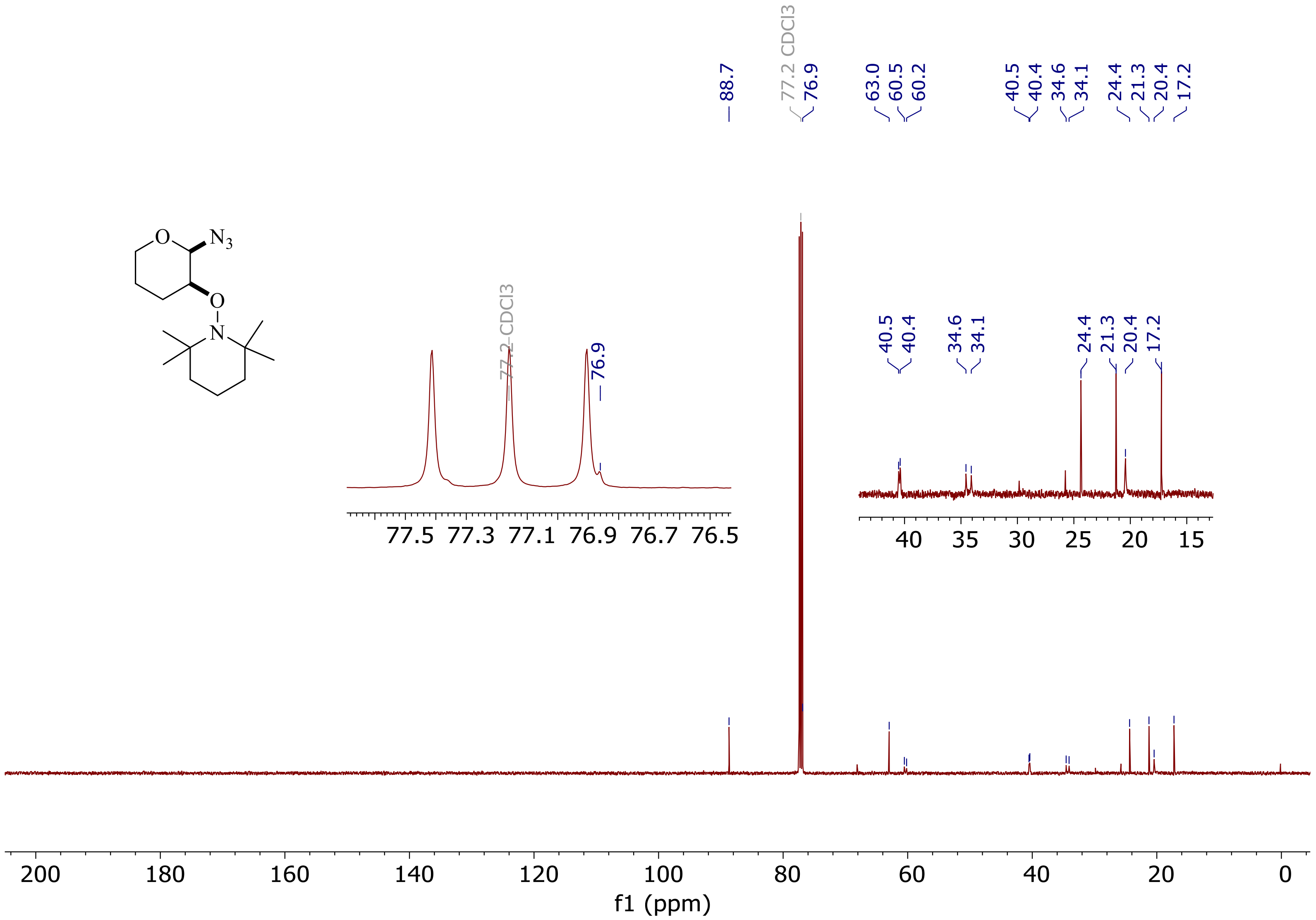

Figure S38. $^{13}C\{^{1}H\}$ NMR of compound *cis*-**21** (125 MHz, $CDCl_3$).

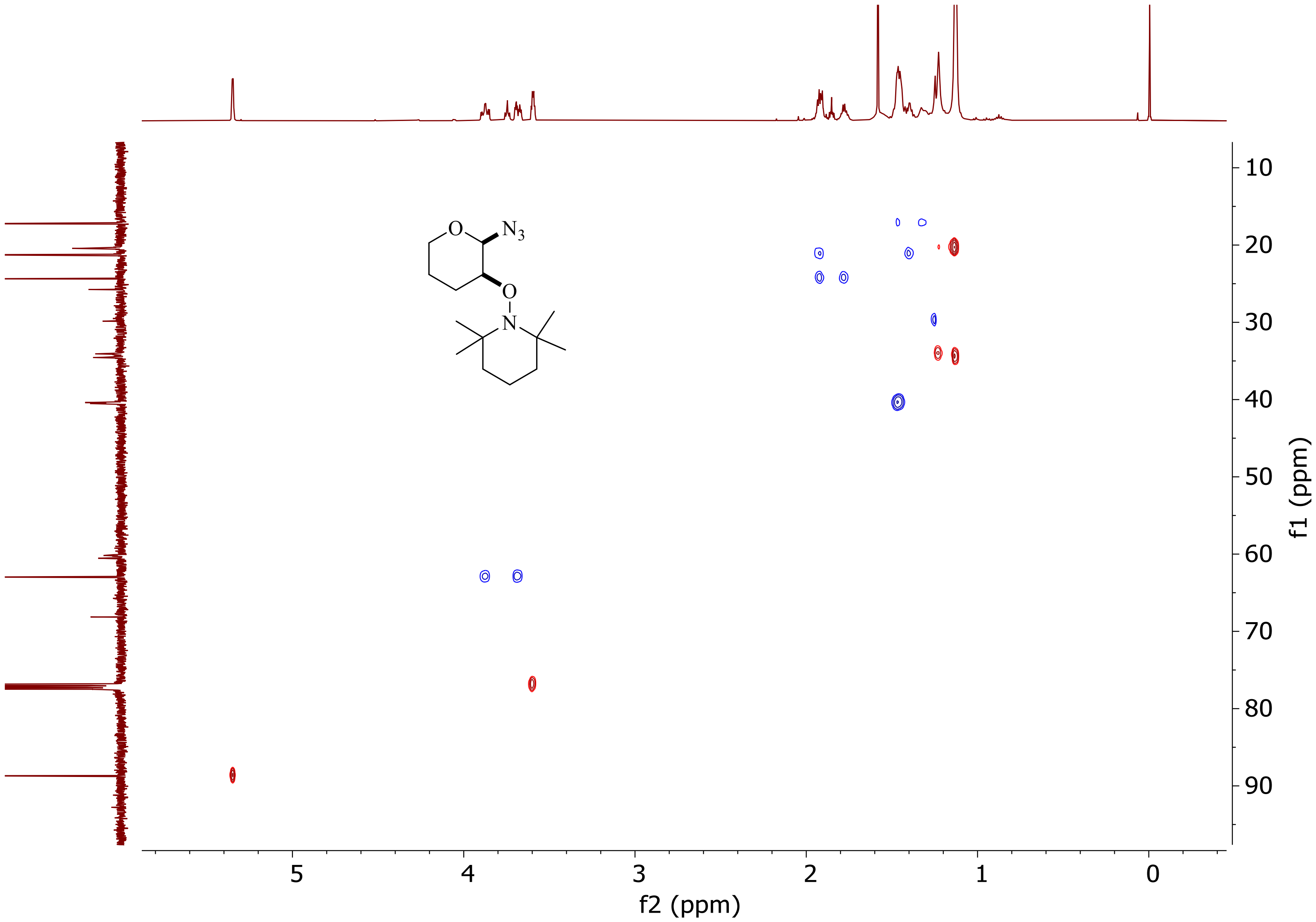

Figure S39. HSQC NMR of compound *cis*-**21** (500 MHz, $CDCl_3$).

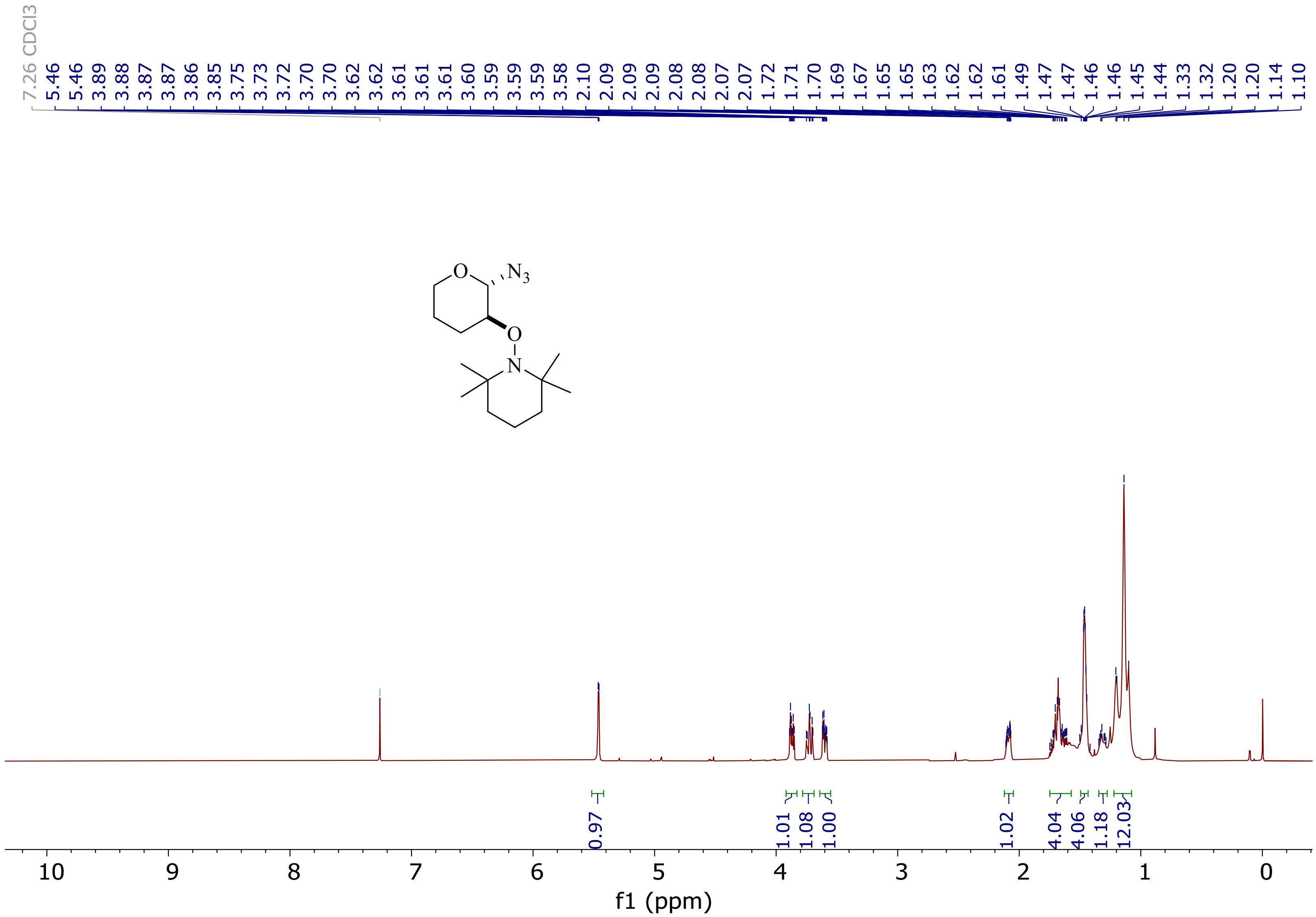

Figure S40. ^{1}H NMR spectrum of compound *trans*-**21** (500 MHz, $CDCl_3$).

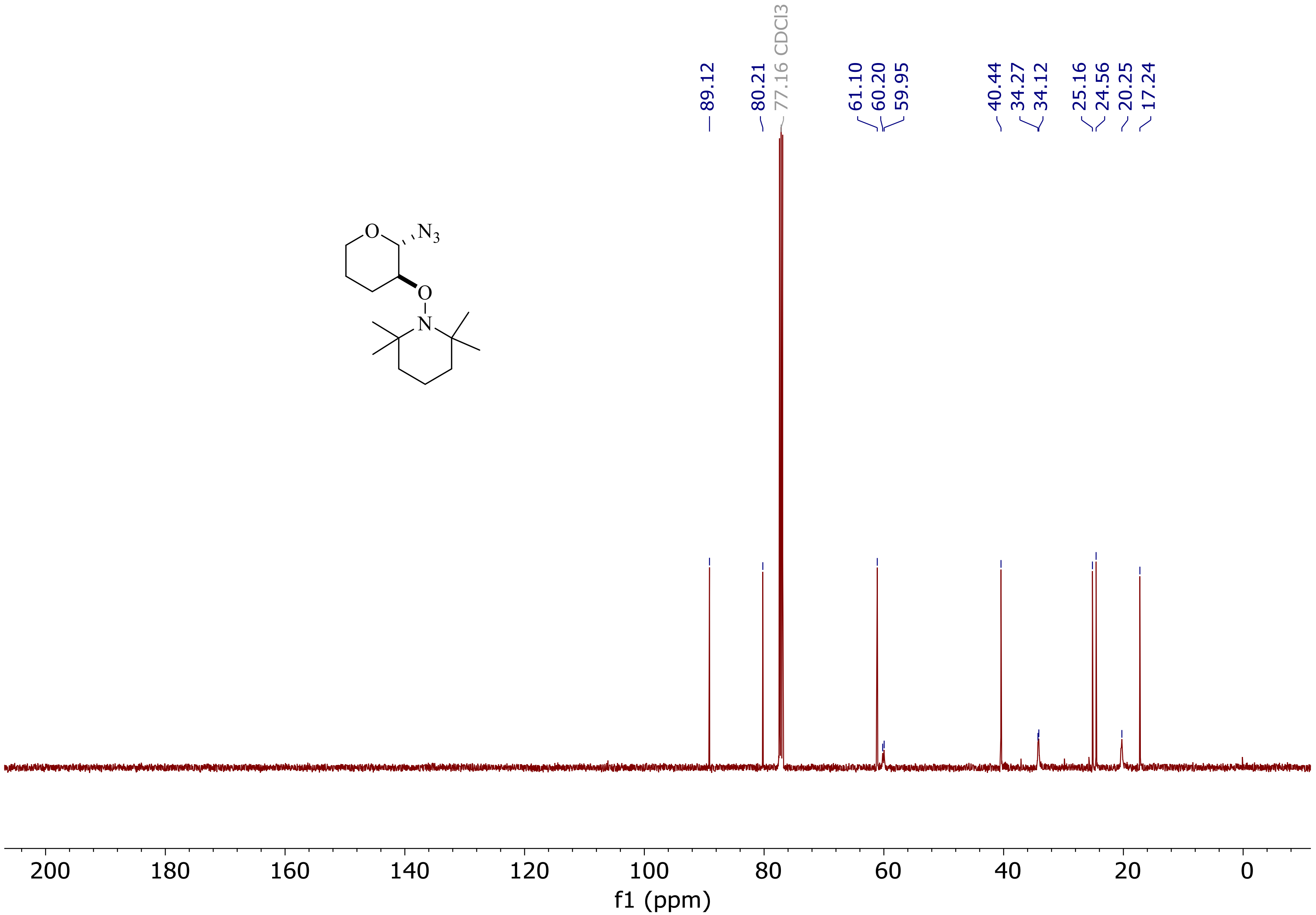

Figure S41. $^{13}C\{^1H\}$ NMR spectrum of compound *trans*-**21** (125 MHz, $CDCl_3$).

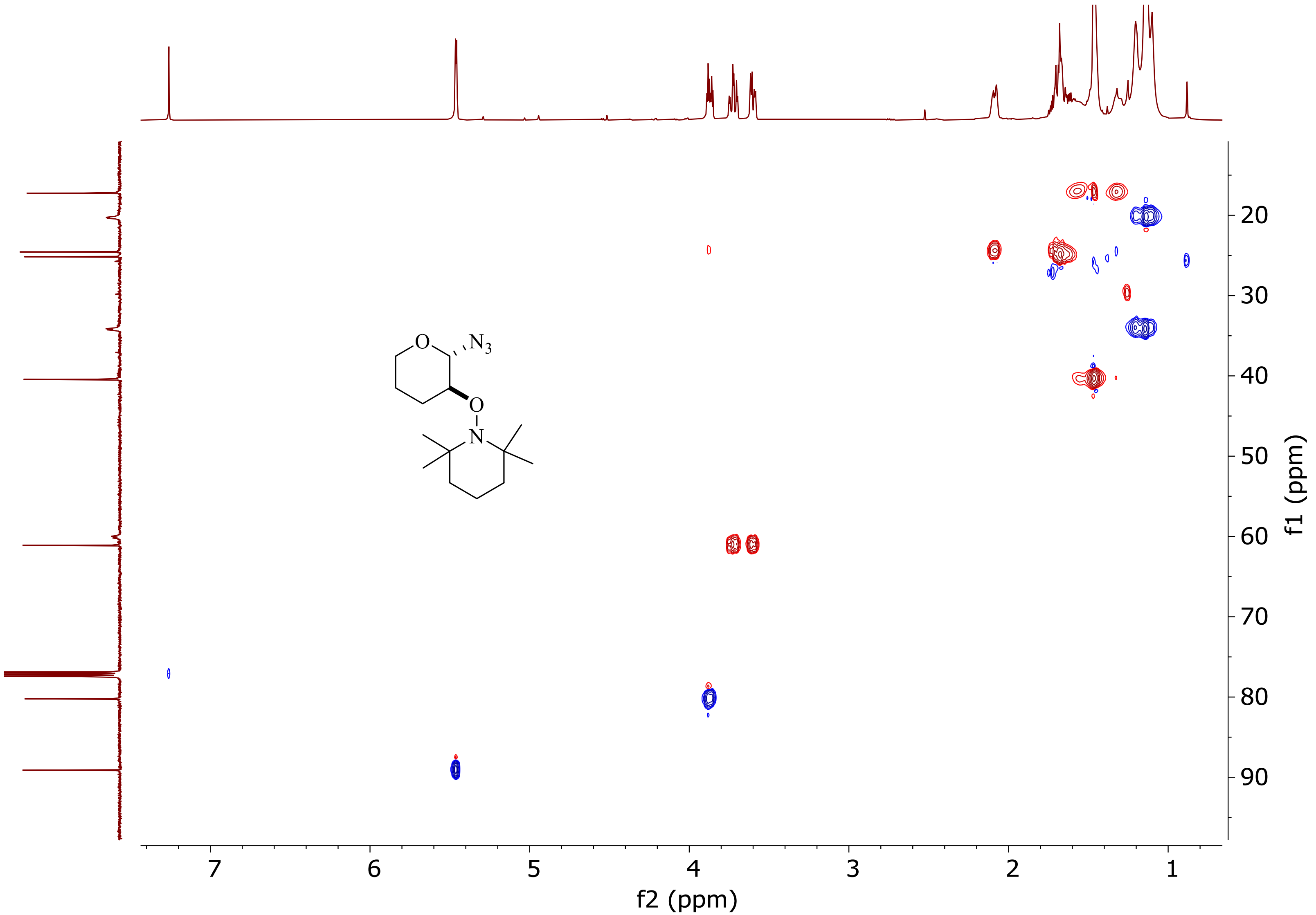

Figure S42. HSQC NMR of compound *trans*-**21** (500 MHz, $CDCl_3$).

4. Optimized Geometries and Complexation Energies

Table S1. XYZ coordinates of structure **4-^{4}H$_{5}$** in vacuum.
As obtained at the M06-2X/6-311+G** level of theory.

Energy + ZPE : -993.124402 ua

Free energy : -993.051515 ua

Atom	X	Y	Z
8	-0.881757000	2.860274000	0.012797000
8	-0.031285000	-0.498346000	-1.050506000
8	2.361157000	0.514271000	0.615747000
8	-2.604171000	0.518485000	0.483434000
8	0.225637000	-1.836501000	0.734755000
8	2.874919000	-1.020258000	-0.947330000
8	-2.861950000	-1.504168000	-0.472673000
6	0.101806000	0.663336000	-0.238218000
6	-0.902994000	1.675814000	-0.776099000
6	1.515000000	1.226410000	-0.304096000
6	-2.318281000	1.129452000	-0.775879000
6	1.492494000	2.658014000	0.141061000
6	0.356795000	3.333742000	0.299654000
6	0.068812000	-1.707544000	-0.451322000
6	2.937164000	-0.628629000	0.187015000
6	-2.811734000	-0.817314000	0.511797000
6	-0.035646000	-2.805478000	-1.459169000
6	3.615291000	-1.339186000	1.319743000
6	-2.922245000	-1.312374000	1.922721000
1	-0.134235000	0.429727000	0.801917000
1	-0.632234000	1.945801000	-1.806532000

1	1.918122000	1.100625000	-1.312699000
1	-2.437986000	0.399551000	-1.574739000
1	-3.020045000	1.953611000	-0.904526000
1	2.432531000	3.130913000	0.390291000
1	0.317447000	4.346730000	0.679239000
1	0.794540000	-2.707452000	-2.161013000
1	-0.975903000	-2.698417000	-2.001062000
1	0.005033000	-3.765779000	-0.952580000
1	2.834607000	-1.849783000	1.889329000
1	4.115012000	-0.631510000	1.979247000
1	4.314333000	-2.071097000	0.923204000
1	-3.412750000	-0.576962000	2.557647000
1	-1.904257000	-1.472446000	2.288433000
1	-3.453375000	-2.261035000	1.930804000

Table S2. XYZ coordinates of structure **4-^{5}H$_4$** in vacuum.
As obtained at the M06-2X/6-311+G** level of theory.

Energy + ZPE : -993.119817 ua

Free energy : -993.169753 ua

Atom	X	Y	Z
8	1.416974000	-0.661852000	2.123976000
8	2.082145000	-0.189691000	-0.638752000
8	-1.109914000	-1.870001000	-0.290160000
8	-1.694821000	0.742098000	0.827312000
8	1.794008000	1.999126000	-1.042533000
8	-1.318170000	-0.898816000	-2.304043000

8	-0.989859000	2.798989000	1.382997000
6	0.727871000	-0.274838000	-0.189847000
6	0.640966000	0.173860000	1.265485000
6	0.327226000	-1.740305000	-0.353741000
6	-0.800189000	0.180342000	1.791674000
6	0.912908000	-2.558079000	0.749829000
6	1.411741000	-1.985311000	1.845529000
6	2.499044000	1.028934000	-1.050831000
6	-1.821983000	-1.375253000	-1.322470000
6	-1.653375000	2.090365000	0.681845000
6	3.930088000	0.979706000	-1.501088000
6	-3.295083000	-1.494247000	-1.059799000
6	-2.521791000	2.523824000	-0.462404000
1	0.105611000	0.364494000	-0.811806000
1	1.069860000	1.171489000	1.355566000
1	0.662301000	-2.080493000	-1.335470000
1	-0.844454000	0.748133000	2.720806000
1	-1.146740000	-0.838878000	1.951722000
1	0.965992000	-3.633171000	0.649340000
1	1.892509000	-2.549500000	2.635199000
1	4.032892000	0.256617000	-2.310977000
1	4.558037000	0.645517000	-0.674177000
1	4.236399000	1.967760000	-1.833450000
1	-3.541768000	-0.846170000	-0.216671000
1	-3.545567000	-2.518995000	-0.784893000
1	-3.846310000	-1.189324000	-1.945629000
1	-3.537362000	2.151538000	-0.321158000
1	-2.130378000	2.089929000	-1.385570000
1	-2.518474000	3.608245000	-0.528294000

Table S3. XYZ coordinates of structure **MC1 (4-$^4H_5$-1)** in vacuum.
As obtained at the M06-2X/6-311+G** level of theory.

Energy + ZPE : -1476.252525 ua

Free energy : -1476.316056 ua

Atom	X	Y	Z
8	-3.566786000	0.240197000	-2.227911000
8	-1.675747000	0.052005000	0.880185000
8	-1.101269000	2.815485000	-0.250870000
8	-3.378131000	-2.072575000	0.577738000
8	-2.215023000	1.734452000	2.271780000
8	0.710722000	1.933152000	0.736303000
8	-1.562161000	-2.877546000	-0.461056000
6	-2.325860000	0.709834000	-0.211961000
6	-2.820256000	-0.366512000	-1.177434000
6	-1.348228000	1.600300000	-0.974549000
6	-3.800166000	-1.390801000	-0.605669000
6	-1.938566000	1.970989000	-2.301032000
6	-2.983609000	1.319694000	-2.804722000
6	-1.743232000	0.647575000	2.098464000
6	-0.082444000	2.843910000	0.612164000
6	-2.266069000	-2.809363000	0.517176000
6	-1.181067000	-0.241610000	3.167185000
6	-0.072950000	4.111916000	1.404303000
6	-1.993630000	-3.535061000	1.804496000
1	-3.158882000	1.313985000	0.159133000
1	-1.959837000	-0.877311000	-1.618441000
1	-0.394536000	1.076700000	-1.079068000
1	-4.012701000	-2.121881000	-1.388579000

1	-4.723248000	-0.884689000	-0.326514000
1	-1.525614000	2.819212000	-2.829093000
1	-3.474515000	1.594409000	-3.729204000
1	-1.024182000	0.338023000	4.073139000
1	-0.249727000	-0.701848000	2.836929000
1	-1.904907000	-1.035136000	3.367175000
1	-0.322948000	4.962139000	0.771574000
1	0.893770000	4.246265000	1.882921000
1	-0.851982000	4.016573000	2.165539000
1	-1.048996000	-3.172308000	2.215744000
1	-1.873753000	-4.596216000	1.584030000
1	-2.792617000	-3.392125000	2.527764000
7	2.668036000	0.024289000	-0.759892000
8	2.128702000	0.851558000	-1.396792000
6	3.919067000	0.414870000	0.029985000
6	2.079619000	-1.387856000	-0.756289000
6	3.243977000	-2.385720000	-0.874067000
1	2.815153000	-3.373274000	-0.686405000
1	3.597624000	-2.384579000	-1.910606000
6	4.955945000	-0.699398000	-0.192681000
1	5.793879000	-0.472682000	0.471220000
1	5.333867000	-0.630382000	-1.218449000
6	4.412129000	-2.100470000	0.061099000
1	4.120344000	-2.225684000	1.106365000
1	5.202208000	-2.830458000	-0.123109000
6	1.133006000	-1.499518000	-1.941681000
1	0.768226000	-2.527366000	-1.967878000
1	0.267250000	-0.848763000	-1.828540000
1	1.634671000	-1.273114000	-2.884325000
6	1.290496000	-1.518681000	0.557796000

1	1.927900000	-1.737802000	1.411644000
1	0.703286000	-0.615565000	0.743694000
1	0.592580000	-2.345515000	0.407613000
6	3.517397000	0.560786000	1.509135000
1	4.345619000	1.080433000	1.994944000
1	2.615688000	1.165732000	1.611018000
1	3.383238000	-0.394499000	2.008863000
6	4.393905000	1.753905000	-0.514401000
1	4.565883000	1.716045000	-1.591511000
1	3.681733000	2.549089000	-0.293518000
1	5.340037000	1.986013000	-0.023728000

Table S4. XYZ coordinates of structure **MC2 (4-$^{5}H_{4}$-1)** in vacuum.
As obtained at the M06-2X/6-311+G** level of theory.

Energy + ZPE : -1476.255306 ua

Free energy : -1476.322323 ua

Atom	X	Y	Z
8	4.799573000	0.367007000	-0.447938000
8	2.648862000	1.991257000	0.302965000
8	1.667234000	-0.960588000	-1.675762000
8	2.447288000	-2.163451000	0.746477000
8	0.458263000	2.353005000	0.595283000
8	-0.295453000	-0.723999000	-0.616827000
8	2.861857000	-1.462277000	2.833648000
6	2.424165000	0.598978000	0.059124000
6	3.744150000	-0.076628000	0.402466000

6	2.062755000	0.414965000	-1.420383000
6	3.696451000	-1.608954000	0.310511000
6	3.265433000	0.687480000	-2.258834000
6	4.490255000	0.650288000	-1.730150000
6	1.589782000	2.769038000	0.576911000
6	0.506083000	-1.411487000	-1.214681000
6	2.149488000	-2.034825000	2.058649000
6	2.014162000	4.180583000	0.848378000
6	0.321094000	-2.864632000	-1.541286000
6	0.821373000	-2.663486000	2.368542000
1	1.619630000	0.242523000	0.702421000
1	4.032085000	0.230913000	1.405842000
1	1.216158000	1.059847000	-1.665937000
1	4.509585000	-2.029030000	0.902966000
1	3.793365000	-1.917544000	-0.728918000
1	3.151249000	0.925749000	-3.306633000
1	5.381857000	0.873247000	-2.302756000
1	1.140348000	4.794063000	1.049637000
1	2.561918000	4.567360000	-0.011979000
1	2.692253000	4.196007000	1.702768000
1	0.354330000	-3.005517000	-2.622804000
1	-0.627955000	-3.219397000	-1.144382000
1	1.144987000	-3.428365000	-1.102047000
1	0.047499000	-2.144059000	1.796945000
1	0.623289000	-2.582124000	3.433895000
1	0.824755000	-3.710644000	2.061843000
7	-3.114856000	0.234614000	-0.698663000
8	-2.534590000	0.320348000	-1.715954000
6	-3.894952000	-1.050761000	-0.414204000
6	-3.091521000	1.424460000	0.259079000

6	-4.549663000	1.677799000	0.687301000
1	-4.504556000	2.427879000	1.480566000
1	-5.085330000	2.132400000	-0.152936000
6	-5.300596000	-0.618995000	0.043951000
1	-5.811054000	-1.530667000	0.364468000
1	-5.847517000	-0.233980000	-0.823415000
6	-5.291106000	0.429383000	1.150578000
1	-4.852648000	0.029124000	2.067483000
1	-6.320318000	0.696064000	1.397065000
6	-2.518291000	2.605616000	-0.507554000
1	-2.585710000	3.475309000	0.147575000
1	-1.468496000	2.447336000	-0.748736000
1	-3.087711000	2.809696000	-1.416230000
6	-2.174281000	1.053545000	1.438631000
1	-2.670264000	0.428903000	2.177139000
1	-1.254628000	0.585862000	1.085905000
1	-1.893042000	1.995382000	1.911535000
6	-3.137113000	-1.846790000	0.661164000
1	-3.523971000	-2.866587000	0.618696000
1	-2.068609000	-1.863177000	0.443629000
1	-3.299983000	-1.467981000	1.666267000
6	-3.944946000	-1.843612000	-1.711650000
1	-4.374545000	-1.261560000	-2.528419000
1	-2.953394000	-2.188327000	-2.007143000
1	-4.580180000	-2.713214000	-1.538060000

Table S5. XYZ coordinates of structure **4-^{4}H$_5$** in acetonitrile.
As obtained at the M06-2X/6-311+G** level of theory.

Energy + ZPE : -993.147583 ua

Free energy : -993.198073 ua

Atom	X	Y	Z
8	-0.945288000	2.799297000	-0.050185000
8	0.010356000	-0.554255000	-1.056532000
8	2.348669000	0.545775000	0.641755000
8	-2.673589000	0.517720000	0.411175000
8	0.271894000	-1.874443000	0.741209000
8	2.956489000	-0.982472000	-0.890040000
8	-2.818432000	-1.582653000	-0.381520000
6	0.093021000	0.623630000	-0.256369000
6	-0.919401000	1.608088000	-0.836091000
6	1.492025000	1.223669000	-0.300204000
6	-2.322893000	1.042040000	-0.875487000
6	1.427958000	2.658003000	0.131215000
6	0.274120000	3.310554000	0.256464000
6	0.117214000	-1.753202000	-0.447574000
6	3.011711000	-0.552124000	0.234557000
6	-2.863371000	-0.809512000	0.542020000
6	0.039295000	-2.865235000	-1.442406000
6	3.807155000	-1.142924000	1.357143000
6	-3.140786000	-1.170161000	1.968383000
1	-0.164350000	0.395344000	0.780228000
1	-0.625913000	1.876521000	-1.859299000
1	1.915526000	1.108103000	-1.300765000
1	-2.390429000	0.262034000	-1.631067000

1	-3.029706000	1.840019000	-1.103136000
1	2.352684000	3.161275000	0.382606000
1	0.205301000	4.329398000	0.618641000
1	0.954835000	-2.857092000	-2.039256000
1	-0.807210000	-2.707649000	-2.110795000
1	-0.052845000	-3.816848000	-0.924008000
1	3.123977000	-1.462725000	2.146738000
1	4.474200000	-0.387113000	1.775077000
1	4.380126000	-1.992796000	0.994098000
1	-4.069237000	-0.690709000	2.285924000
1	-2.336058000	-0.796757000	2.604063000
1	-3.227980000	-2.249716000	2.064144000

Table S6. XYZ coordinates of structure **4-$^{5}H_{4}$** in acetonitrile.
As obtained at the M06-2X/6-311+G** level of theory.

Energy + ZPE : -993.144612 ua

Free energy : -993.194356 ua

Atom	X	Y	Z
8	1.446309000	-0.504426000	2.149934000
8	2.114091000	-0.152842000	-0.608038000
8	-1.054159000	-1.866809000	-0.227681000
8	-1.734282000	0.715263000	0.824974000
8	1.828425000	1.999261000	-1.169276000
8	-1.240790000	-1.060822000	-2.312091000
8	-1.114436000	2.838532000	1.185081000
6	0.751165000	-0.234785000	-0.175931000
6	0.645723000	0.274802000	1.258077000

6	0.381697000	-1.715131000	-0.277839000
6	-0.789593000	0.258620000	1.798320000
6	0.968478000	-2.471246000	0.868747000
6	1.456549000	-1.843341000	1.939426000
6	2.542530000	1.033298000	-1.087131000
6	-1.759540000	-1.469538000	-1.302676000
6	-1.798967000	2.041524000	0.596402000
6	3.983698000	0.965996000	-1.483563000
6	-3.231093000	-1.619830000	-1.072286000
6	-2.813346000	2.351989000	-0.459723000
1	0.125989000	0.369191000	-0.831017000
1	1.052500000	1.284272000	1.307200000
1	0.738568000	-2.101137000	-1.234202000
1	-0.847435000	0.874613000	2.695825000
1	-1.090675000	-0.761129000	2.031842000
1	1.036856000	-3.549899000	0.817372000
1	1.939535000	-2.365502000	2.757124000
1	4.123937000	0.172604000	-2.219621000
1	4.586448000	0.722376000	-0.606407000
1	4.295246000	1.922436000	-1.896238000
1	-3.510541000	-1.088974000	-0.161059000
1	-3.467382000	-2.677408000	-0.935384000
1	-3.778422000	-1.226054000	-1.925737000
1	-3.795344000	2.004384000	-0.132442000
1	-2.556189000	1.817614000	-1.377079000
1	-2.838188000	3.423204000	-0.644333000

Table S7. XYZ coordinates of structure **MC1 (4-$^4H_5$-1)** in acetonitrile.
As obtained at the M06-2X/6-311+G** level of theory.

Energy + ZPE : -1476.345974 ua

Free energy : -1476.410497 ua

Atom	X	Y	Z
8	-3.563465000	-0.217407000	-2.008230000
8	-1.499027000	0.193035000	0.955147000
8	-1.292213000	2.844742000	-0.557177000
8	-2.977399000	-2.105355000	1.031617000
8	-2.118758000	1.914902000	2.256048000
8	0.580489000	2.283638000	0.548518000
8	-1.883850000	-3.479498000	-0.359218000
6	-2.284822000	0.659073000	-0.142641000
6	-2.755214000	-0.586228000	-0.891821000
6	-1.429963000	1.508973000	-1.076361000
6	-3.654922000	-1.507548000	-0.073843000
6	-2.093159000	1.612559000	-2.414671000
6	-3.085453000	0.800336000	-2.769651000
6	-1.490894000	0.898722000	2.102288000
6	-0.258245000	3.104364000	0.260113000
6	-2.073417000	-3.058340000	0.755792000
6	-0.632700000	0.233504000	3.131750000
6	-0.299391000	4.515152000	0.753670000
6	-1.345669000	-3.490360000	1.990300000
1	-3.139922000	1.237403000	0.217132000
1	-1.877494000	-1.131054000	-1.261164000
1	-0.433377000	1.067598000	-1.148255000
1	-4.053336000	-2.280266000	-0.733198000

1	-4.476270000	-0.936709000	0.358951000
1	-1.764096000	2.384989000	-3.097642000
1	-3.611705000	0.877804000	-3.713346000
1	-0.370739000	0.949698000	3.907893000
1	0.263448000	-0.185507000	2.672915000
1	-1.206534000	-0.584692000	3.576465000
1	-0.389986000	5.202600000	-0.088553000
1	0.598710000	4.731465000	1.327218000
1	-1.182833000	4.635816000	1.384844000
1	-0.711879000	-2.663700000	2.323580000
1	-0.731143000	-4.360824000	1.771879000
1	-2.054724000	-3.712149000	2.788954000
7	2.547765000	0.068781000	-0.769278000
8	2.035954000	0.926019000	-1.391506000
6	3.668590000	0.446727000	0.197551000
6	2.073154000	-1.367734000	-0.986421000
6	3.328110000	-2.249814000	-1.100419000
1	2.965347000	-3.280373000	-1.103504000
1	3.793226000	-2.057892000	-2.072493000
6	4.807056000	-0.567625000	-0.003024000
1	5.524394000	-0.369364000	0.796884000
1	5.300039000	-0.345981000	-0.954741000
6	4.346405000	-2.019092000	0.007865000
1	3.933986000	-2.293852000	0.981769000
1	5.208902000	-2.665773000	-0.163982000
6	1.259573000	-1.393991000	-2.271045000
1	1.029800000	-2.441567000	-2.473352000
1	0.318423000	-0.849772000	-2.173809000
1	1.826181000	-0.993307000	-3.113530000
6	1.181857000	-1.747187000	0.207117000

1	1.748270000	-1.976506000	1.106430000
1	0.447146000	-0.964669000	0.413539000
1	0.648277000	-2.649286000	-0.100524000
6	3.084042000	0.412922000	1.620168000
1	3.810906000	0.932302000	2.248078000
1	2.138372000	0.954433000	1.664317000
1	2.960988000	-0.594574000	2.008129000
6	4.105920000	1.860565000	-0.149019000
1	4.403420000	1.941784000	-1.196179000
1	3.319636000	2.585194000	0.065859000
1	4.971204000	2.084798000	0.476609000

Table S8. XYZ coordinates of structure **MC2 (4-$^5H_4$-1)** in acetonitrile.
As obtained at the M06-2X/6-311+G** level of theory.

Energy + ZPE : -1476.345176 ua

Free energy : -1476.410497 ua

Atom	X	Y	Z
8	4.882658000	0.007096000	-0.066087000
8	2.846354000	1.911202000	0.221474000
8	1.806149000	-1.054134000	-1.695808000
8	2.082973000	-2.128930000	0.899933000
8	0.683708000	2.490254000	0.367996000
8	-0.208182000	-0.493231000	-0.888249000
8	2.364172000	-1.562398000	3.048574000
6	2.498418000	0.532327000	0.048156000
6	3.661977000	-0.256226000	0.631757000

6	2.333798000	0.271919000	-1.454353000
6	3.441974000	-1.774952000	0.614912000
6	3.674589000	0.330052000	-2.111805000
6	4.797834000	0.203190000	-1.403585000
6	1.845586000	2.804270000	0.366726000
6	0.536575000	-1.320565000	-1.358802000
6	1.645488000	-1.942881000	2.159401000
6	2.384575000	4.191668000	0.524300000
6	0.175725000	-2.737673000	-1.683310000
6	0.177772000	-2.214606000	2.268654000
1	1.575535000	0.317723000	0.585782000
1	3.832579000	0.089230000	1.650514000
1	1.636979000	1.002791000	-1.870641000
1	4.110409000	-2.249791000	1.333477000
1	3.633795000	-2.168564000	-0.381694000
1	3.743194000	0.486038000	-3.180187000
1	5.782818000	0.270436000	-1.850576000
1	1.563289000	4.890950000	0.660711000
1	2.960436000	4.458104000	-0.364131000
1	3.056780000	4.226295000	1.383478000
1	-0.020229000	-2.803661000	-2.757361000
1	-0.720946000	-3.030086000	-1.140458000
1	1.003854000	-3.404192000	-1.444789000
1	-0.347920000	-1.327010000	1.902093000
1	-0.090656000	-2.384924000	3.309207000
1	-0.109764000	-3.063330000	1.647892000
7	-3.132803000	0.356589000	-0.729127000
8	-2.590601000	0.541044000	-1.755214000
6	-3.844910000	-0.978292000	-0.511995000
6	-3.120843000	1.472868000	0.310216000

6	-4.561803000	1.618519000	0.831300000
1	-4.503928000	2.311088000	1.674343000
1	-5.158094000	2.097749000	0.048322000
6	-5.239450000	-0.654334000	0.050655000
1	-5.679724000	-1.616642000	0.323131000
1	-5.844438000	-0.227884000	-0.755799000
6	-5.214785000	0.303441000	1.234885000
1	-4.701599000	-0.141740000	2.090403000
1	-6.240815000	0.500920000	1.551331000
6	-2.651690000	2.739225000	-0.386272000
1	-2.752084000	3.550161000	0.337311000
1	-1.604850000	2.668972000	-0.681638000
1	-3.270763000	2.969778000	-1.255311000
6	-2.115498000	1.072785000	1.402438000
1	-2.486696000	0.290226000	2.059153000
1	-1.161998000	0.781741000	0.957512000
1	-1.954408000	1.973570000	1.998006000
6	-2.992419000	-1.817509000	0.455222000
1	-3.289523000	-2.856096000	0.296866000
1	-1.932710000	-1.717385000	0.222213000
1	-3.166076000	-1.568888000	1.499380000
6	-3.929600000	-1.667313000	-1.863558000
1	-4.420213000	-1.037004000	-2.607324000
1	-2.942718000	-1.958812000	-2.227186000
1	-4.527635000	-2.568590000	-1.720749000

Table S9. Complexation Energy and BSSE Corrected Complexation Energy.
As obtained at the M06-2X/6-311+G** level of theory.

Parameter	Vacuum		Acetonitrile	
	MC1	MC2	MC1	MC2
Complexation electronic energy with ZPE, kcal/mol	-19.66	-19.74	-5.00	-4.43
Complexation energy, kcal/mol	-20.20	-20.44	-16.20	-18.96
BSSE-corrected complexation energy, kcal/mol	-18.44	-19.03	-14.38	-17.61
